# The unusual gene architecture of polyubiquitin is created by dual-specific splice sites

**DOI:** 10.1186/s13059-023-03157-8

**Published:** 2024-01-24

**Authors:** Chaorui Duan, Truman Mooney, Luke Buerer, Cory Bowers, Stephen Rong, Seong Won Kim, Alger M. Fredericks, Sean F. Monaghan, William G. Fairbrother

**Affiliations:** 1 Department of Molecular Biology, Cell Biology, and Biochemistry, Brown University, Providence, RI 02903 USA; 2 Center for Computational Molecular Biology, Brown University, Providence, RI 02903 USA; 3 Department of Surgery, The Miriam Hospital, Providence, RI 02906 USA; 4 Division of Surgical Research, Department of Surgery, Alpert Medical School of Brown University/Rhode Island Hospital, Providence, RI 02903 USA

**Keywords:** Dual-specific splice sites, Synergy, RNA splicing, *UBC*

## Abstract

**Background:**

The removal of introns occurs through the splicing of a 5′ splice site (5′ss) with a 3′ splice site (3′ss). These two elements are recognized by distinct components of the spliceosome. However, introns in higher eukaryotes contain many matches to the 5′ and 3′ splice-site motifs that are presumed not to be used.

**Results:**

Here, we find that many of these sites can be used. We also find occurrences of the AGGT motif that can function as either a 5′ss or a 3′ss—previously referred to as dual-specific splice sites (DSSs)—within introns. Analysis of the Sequence Read Archive reveals a 3.1-fold enrichment of DSSs relative to expectation, implying synergy between the ability to function as a 5′ss and 3′ss. Despite this suggested mechanistic advantage, DSSs are 2.7- and 4.7-fold underrepresented in annotated 5′ and 3′ splice sites. A curious exception is the polyubiquitin gene *UBC*, which contains a tandem array of DSSs that precisely delimit the boundary of each ubiquitin monomer. The resulting isoforms splice stochastically to include a variable number of ubiquitin monomers. We found no evidence of tissue-specific or feedback regulation but note the 8.4-fold enrichment of DSS-spliced introns in tandem repeat genes suggests a driving role in the evolution of genes like *UBC*.

**Conclusions:**

We find an excess of unannotated splice sites and the utilization of DSSs in tandem repeats supports the role of splicing in gene evolution. These findings enhance our understanding of the diverse and complex nature of the splicing process.

**Supplementary Information:**

The online version contains supplementary material available at 10.1186/s13059-023-03157-8.

## Background

Pre-mRNA splicing, a critical step in the processing of eukaryotic genes, involves the removal of introns and the joining of adjacent exons. The process begins with the identification of the 5′ and 3′ splice sites located at the exon–intron boundaries, with the 5′ss recognized by U1 small nuclear ribonucleoprotein (snRNP) through base-pairing with the 5′ end of U1 small nuclear RNA (snRNA) [1–3]. The typical consensus motif for the 5′ss is AG|GTRAGT [4]. The recognition of the 3′ss involves three components: the branch point (BP), the polypyrimidine tract (PPT), and the conserved 3′ss with a consensus sequence of Y_10_NCAG|G [5–7]. Proteins such as SF1, U2AF65, and U2AF35 recognize the BP sequence, the PPT, and the 3′ss, respectively [8–10]. The U2 snRNP is then recruited to the BP sequence by U2AF, joining with U1 snRNP and other splicing factors to strengthen the recognition of both the 5′ and 3′ splice sites. Higher-order models of splice site recognition describe the coordinated recognition of the 5′ss and 3′ss either across the exon in the exon definition model [11–13] or across the intron in the intron definition model [12, 14].

Research suggests the spliceosome can be assembled through either a U1-first pathway or a U2-first pathway [15]. The reversibility of the spliceosome assembly both increases flexibility and makes observation of splice site selection and the interpretation of discovered intermediates like lariat introns more challenging [16, 17]. While these and similar observations suggest an increasingly complex model of recognition, the prevailing belief has been that the removal of introns occurs as a complete entity, facilitated by the catalytic pathway that paired the 5′ss at the beginning of the intron to the 3′ss at the end of the intron. However, recent evidence suggests that intron removal can occur in sections, either through recursive splicing (i.e., sequential splices to AG|GT motifs that reconstitute splice sites for an additional splice) or introns-within-introns [18–20]. Similarly, dual-specific splice sites (DSSs) can function as either 5′ or 3′ splice sites (for differences between recursive splice sites and DSSs, see Additional file 1: Fig. S1) [21, 22]. While these discoveries suggest additional layers of splicing activity that is not apparent from the final annotation, we do not know the extent to which splicing outside of the annotated sites occurs in typical intron removal.

In this study, we assay the ability of each position in a full-length pre-mRNA to serve as a splice site using minigene splicing reporters and compare the results to events detected in vivo across more than 20 thousand RNA-seq experiments in the Sequence Read Archive (SRA) [23]. Among the excess of observed splicing events, we characterized the case where a single element can function as two different types of splice sites (i.e., DSSs). We detailed how the alternative splicing of the human *UBC* gene occurs through a series of DSSs and how tandem repeats that contain DSSs can drive gene evolution.

## Results

### Splicing occurs more frequently than suggested by annotation

**Fig. 1:**
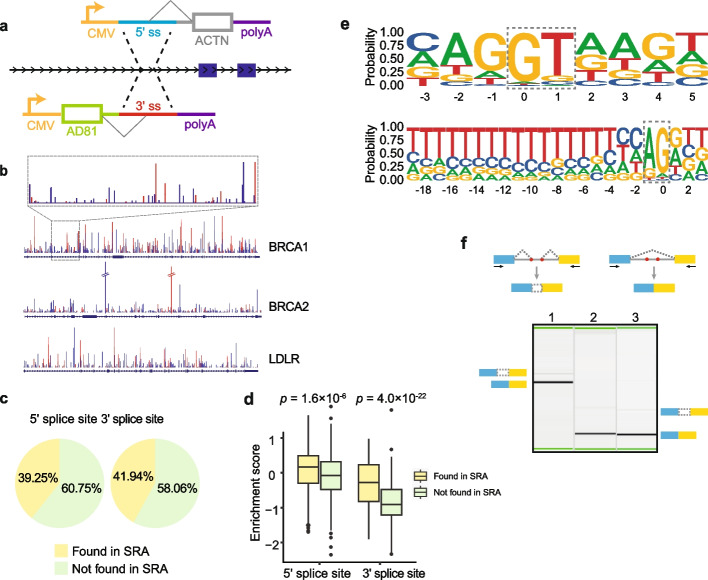
Massively parallel reporter assay (MPRA) reveals a high frequency of cryptic splice sites. **a** Schematic of MPRA employed for cryptic splice site identification. **b** Splice sites identified in the MPRA on *BRCA1*, *BRCA2*, and *LDLR*. The blue and red bars represent 5′ splice sites (5′ss) and 3′ splice sites (3′ss), respectively. The height of the bars corresponds to the original enrichment score (not log10 transformed), with taller bars indicating higher scores. The plot within the dashed box provides a zoomed-in representation of the splice sites identified in *BRCA1* intron 3. **c** The proportion of splice sites identified in the MPRA that were found in the Sequence Read Archive (SRA). **d** Comparison of enrichment scores for splice sites found in SRA and those not found in SRA (Mann–Whitney test). **e** Sequence logos depicting the consensus sequence for cryptic 5′ and 3′ splice sites identified in the MPRA. The dotted boxes represent the dinucleotides present in the 5′ and 3′ splice sites. **f** RT-PCR validation of pseudo-exons created by cryptic sites in *BRCA2* using TaqMan® Control Total RNA. Each lane represents one of three pseudo-exons created by cryptic sites identified in our MPRA. The blue and yellow boxes represent exons and the gray lines denote intron, the red dots indicate cryptic splice sites and arrows represent the primers used for RT-PCR

The prevailing model of pre-mRNA splicing describes the removal of annotated introns by a two-step process catalyzed by the spliceosome. However, recent findings suggest the spliceosome can act multiple times on individual introns [24]. To examine the pervasiveness of this mode of splicing, we analyzed a database of splicing events derived from splice junctions observed in 21,504 RNA-seq experiments from the SRA [23]. We found an excess of splicing events in these sequencing samples (42,882,032 observed splicing events vs 288,518 annotated introns) with many splice junctions indicating the presence of smaller novel introns within annotated introns. To confirm this excess of unannotated splice sites, we designed two massively parallel reporter assays (MPRAs) to test the ability of every sequence within 3 genes of interest (*BRCA1*, *BRCA2*, and *LDLR*) to function as a 5′ or 3′ splice site (Fig. 1a). For each gene, we extracted all successive 150-nucleotide windows tiled by 20-nucleotide increments across the gene of interest. This procedure yields a total of 6290 DNA oligonucleotides (oligos) for *BRCA1*, 4251 for *BRCA2*, and 2215 for *LDLR*. In the 5′ss-testing library, each tile was paired with a common 3′ss in order to test for the ability of sequences within that window to function as a 5′ss. In the 3′ss-testing library, each tile was paired with a common 5′ss in order to test for the ability of sequences within that window to function as a 3′ss. Due to the tile length and increment size, each position within the transcript was tested in the 6 or 7 different tiling registers. Splicing efficiencies were measured as enrichments, with log10 ratios of the relative representation of spliced product in the output normalized by the relative representation of parent species in the input library. Comparing the results to splice site usage observed in the reference annotation, we found that almost all the canonical splice sites of the three genes were identified in our MPRA. In comparison to canonical sites, we observed approximately ten-fold as many cryptic sites (Fig. 1b and Table 1). A large proportion of the detected splice sites (39.25% of 5′ss; 41.94% of 3′ss) were supported by observed usage in the SRA (Fig. 1c). The enrichment scores for 5′ and 3′ splice sites present in the SRA were significantly higher than those not identified in the SRA (*p* < 10^−5^, *p* < 10^−21^, respectively, see Fig. 1d). Moreover, the sequence motifs of the cryptic 5′ and 3′ splice sites identified in our assay resembled the motifs of the canonical 5′ and 3′ splice sites (whereas the cryptic 3′ motif shows less preference at the upstream adjacent position (i.e., “C” in “CAG”)), implying that the splicing machinery in the cell has the ability to recognize these cryptic sites in a similar manner to canonical sites (Fig. 1e). This recognition of cryptic splice sites raises the possibility of detecting alternative transcript isoforms using these sites in the human genome. To confirm this, we performed RT-PCR on the *BRCA2* gene and were able to detect low levels of transcript isoforms that used the unannotated splice sites identified by our MPRA (Fig. 1f).

**Table 1:** The number of splice sites identified in our MPRA

Genes	Canonical splice sites		Cryptic splice sites	
	5′ss	3′ss	5′ss	3′ss
*BRCA1*	21	19 (2)^a^	433	266
*BRCA2*	24 (2)^a^	22 (4)^a^	319	167
*LDLR*	17	17	167	148

^a^The numbers in parentheses represent splice sites that were not identified in the MPRA

### Identification and characterization of dual-specific splice sites in vitro and in vivo

**Fig. 2:**
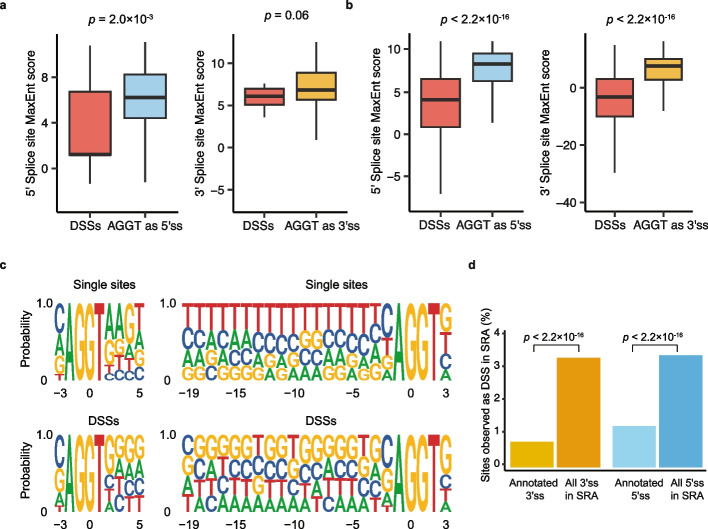
Dual-specific splice sites (DSSs) are used as both 5′ss and 3′ss and are underrepresented in annotated splice sites. **a** The distribution of 5′ss (left) and 3′ss (right) MaxEnt scores for splice sites with an AGGT motif that function as either DSSs or single splice sites from our MPRA (Mann–Whitney test). **b** The distribution of 5′ss (left) and 3′ss (right) MaxEnt scores for splice sites with an AGGT motif that function as either DSSs or single splice sites from SRA splice junction data (Mann–Whitney test). **c** The 5′ss (left) and 3′ss (right) motifs of single splice sites with an AGGT motif (top) compared to DSSs (bottom) from the SRA data. **d** The percentage of annotated 5′ss and 3′ss compared to all 5′ss and 3′ss in the SRA junction reads (minimal threshold > 10 reads) which exhibited DSS activity (> 10 reads supporting 5′ss usage and > 10 supporting 3′ss usage). *p*-values were calculated from the chi-square test of independence

In our MPRA, we discovered 24 loci containing a core AGGT sequence which can be used as either 5′ or 3′ splice sites, also known as DSSs. Given the overlap of the two types of sites within each DSS, it is possible that there is competition between their use as a 5′ss and their use as a 3′ss. Alternatively, it is also possible that factors associated with the recognition of each site could recruit factors to the other site as occurs during exon definition and formation of the A complex [13]. Interestingly, we found that the DSSs have a lower agreement to 5′ss and 3′ss consensus motifs compared to occurrences of AGGT that are only used as a 5′ss or 3′ss (MaxEnt score, Fig. 2a). To determine whether this is true in vivo, we analyzed instances of AGGT splicing events in an expansive subset of samples from the SRA [23]. In agreement with the DSSs recovered from our MPRA, the DSSs present in the SRA data tend to have weaker splice sites both quantitatively (Fig. 2b) and qualitatively (Fig. 2c) than AGGTs which function as only one type of splice site. Given the weaker splice sites of DSSs, these sites may simply be less active than single splice sites. In order to control for this, we used junction reads counts from the SRA as a proxy for splicing activity and found that even at the same level of read support DSSs have lower MaxEnt scores than single sites (Additional file 1: Fig. S2). The definition of an enhancer is the ability of a cis element to compensate for a suboptimal site [25]. Following this definition, the SRA read analysis suggests that either site within a DSS can function as an enhancer for the other.

To further test the synergy between 5′ss and 3′ss, we tested if sites observed to function as a 5′ss or 3′ss would be more, less, or equally likely to also function as a 3′ss or 5′ss, respectively. Dependency between 5′ss recognition and 3′ss recognition in AGGT motifs was measured at various supporting read count thresholds for calling splicing activity (e.g., > 10 reads, > 100 reads). For example, 6.9% of AGGTs were observed to function as a 5′ss and 3.5% as a 3′ss when requiring over 10 supporting reads (Table 2). We found significant positive dependency between 5′ss and 3′ss usage (Fisher’s exact test, *p* < 2.2 × 10^−16^ and chi-square test of independence, *p* < 2.2 × 10^−16^). These sites function as DSSs 3.1-fold higher than expected (> 10 reads), confirming the synergy between 5′ss recognition and 3′ss recognition. This estimate is stable across different thresholds and conservative as instances of recursive splicing undercount splicing events.

**Table 2:** The number of dual-specific splice sites identified in the Sequence Read Archive

	AGGT as 5′ss^a^	AGGT as 3′ss	DSSs
Reads > 10	871,690	441,107	105,875
Reads > 50	434,604	172,456	29,048
Reads > 100	333,691	122,639	16,897

^a^The total number of AGGT in human transcripts (based on the GENCODE v32 annotation) is 12,703,961

Taken together, the data suggest DSSs confer a recognition advantage in addition to the greater versatility of being able to function as either a donor or acceptor site. This greater versatility could also be a liability for a constitutive splice site as an additional splicing event would disrupt the coding region. To test whether DSSs are avoided in annotated junctions, the SRA was used to detect DSSs in annotated sites. Using a threshold of > 10 5′ss junction reads and > 10 3′ss junction reads to call a DSS, we find that 3199 of 253,711 (1.26%) annotated 5′ splice sites function as DSSs, while 1971 of 276,517 (0.713%) annotated 3′ splice sites function as DSSs. In contrast, out of 3,087,201 5′ splice sites and 3,156,210 3′ splice sites in the SRA with > 10 junction reads, 105,875 sites function as DSSs (3.43% of 5′ss and 3.35% of 3′ss; Fig. 2d). Thus, the prevalence of DSSs in annotated 5′ splice sites and 3′ splice sites is 2.7-fold and 4.7-fold lower than observed in unannotated 5′ splice sites and 3′ splice sites, respectively (chi-square test of independence, *p* < 2.2 × 10^−16^).

To provide additional evidence that these unannotated splice sites are utilized, we looked for lariat reads that originated from splicing events involving these DSSs. We modified our lariat-seq mapping pipeline to detect lariats containing 5′ splice sites from the DSSs supported by > 10 junction reads. We then applied this pipeline to RNA-seq data collected from a HEK293T-derived DBR1 knockout cell line as these cells have particularly elevated lariat levels. This method recovered 55 unannotated DSSs from the SRA for which we found lariats utilizing their 5′ splice sites (Additional file 1: Table S1). These lariat reads provide a further indication of the usage of unannotated DSSs beyond the splice junction data present in the SRA.

### *UBC* is processed via a tandem array of DSSs

**Fig. 3:**
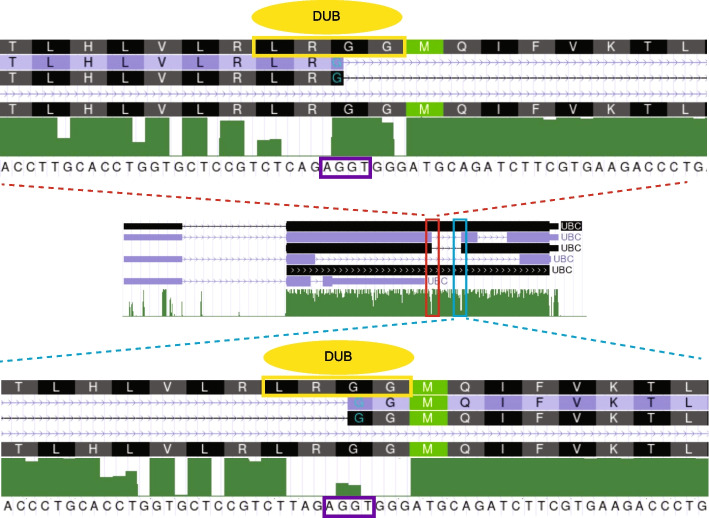
The UCSC annotation (based on the hg19 genome) of *UBC* shows multiple isoforms with DSSs functioning as 5′ss, 3′ss, or neither. DSSs (highlighted in the purple box) are located between the second and third amino acids upstream of each monomer coding region. The “M” inside the green box represents the first amino acid in the ubiquitin monomers. The red and blue boxes indicate the DSSs functioning as 5′ and 3′ splice sites within some but not all transcripts. The green bars represent the phastCons score, indicating conservation among 100 vertebrates. The yellow box represents the LRGG motif recognized by deubiquitinating enzymes (DUBs) (yellow oval)

**Fig. 4:**
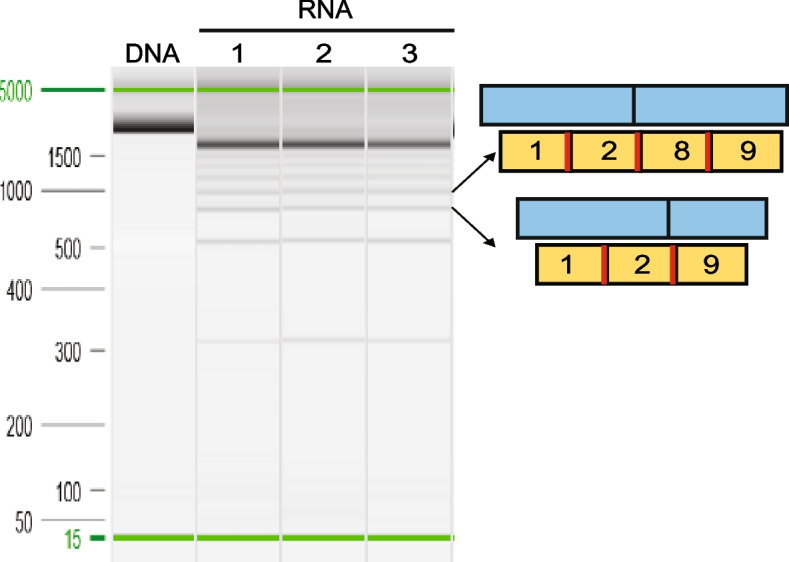
Detection of transcripts processed by DSSs in *UBC*. RT-PCR amplification of *UBC* from HEK293 cells reveals a ladder of bands. The experiment was performed in three biological replicates. The schematic on the right side displays the composition of the transcript isoforms, which were sequenced through Sanger sequencing. The yellow boxes denote the ubiquitin units, the red bar indicates the AGGT motif, and the numbers in the yellow boxes represent the order of ubiquitin monomers. The blue boxes represent exons within which ubiquitin units are located

We identified a high density of DSSs in the unusual gene architecture of *UBC*, the human polyubiquitin gene. *UBC* contains 9 copies of ubiquitin monomers separated by DSSs which are located between the second and third amino acids (i.e., arginine (R) and glycine (G)) upstream of each monomer coding region (Fig. 3). These two amino acids are part of the LRGG binding motif, a cleavage site recognized by deubiquitinating enzymes (DUBs) [26–28]. DUBs are responsible for both removing polyubiquitin chains from substrate proteins and generating free ubiquitin monomers, playing a crucial role in the regulation of the ubiquitin–proteasome system. The UCSC annotation (based on the hg19 genome) [29] indicated multiple isoforms where each DSS functioned as a 5′ss, a 3′ss, or neither (Fig. 3). Interestingly, the DSSs exhibited a low level of conservation suggesting relaxed selection across species on the precise splicing patterns. As alignments of tandem repeats can lead to artifacts, we performed RT-PCR on total RNA from HEK293 cells to confirm the annotated processing pattern of the *UBC* gene. We also tested genomic DNA to confirm that the laddered pattern was not originating from ubiquitin pseudogenes in the genome or an artifact of PCR. Consistent with the annotation, the *UBC* RT-PCR and capillary electrophoresis returned a ladder of bands whose difference in size corresponded to a single ubiquitin unit (Fig. 4). While ubiquitin’s amino acid sequence is highly conserved, variations in the monomers’ DNA sequences allow for manual validation of the alignments to infer which splice sites were used. Sanger sequencing identified one isoform consisting of 4 tandem ubiquitin units, while another one contains 3 tandem units, with the difference explained by the precise removal of the monomer by the spliceosome (Fig. 4 and Additional file 1: Table S2). Ensembl [30] contains additional examples of differences in ubiquitin copy number generated by DSSs splicing (e.g., ENST00000538617.5).

Interestingly, the DSSs are located precisely at 228-nucleotide intervals that fall between the boundaries of each ubiquitin coding region so each ubiquitin monomer exists as a discrete exon (or intron). The consequence of interspersed DSSs is a ubiquitin coding region that can be removed as an intron or retained as an exon without changing the reading frame of the transcript, resulting in different amounts of ubiquitin in the final mRNA transcript. This unusual architecture suggests the amount of ubiquitin in a cell could be controlled by splicing. There have been reports of *UBC* expression regulated by negative feedback from the ligatable monomer [31]. If this regulation were achieved by splicing, we would anticipate a reduction of splicing when overexpressing the ubiquitin gene. To test this, we transfected different levels of an HA-tagged ubiquitin expression vector into HEK293 cells and examined the effect on *UBC* splicing. However, despite demonstration of elevated levels of ligatable monomers (Additional file 1: Fig. S3a), no notable variations in the splicing pattern were observed (Additional file 1: Fig. S3b). We also did not observe a difference in *UBC* splicing in different tissues in mice (Additional file 1: Fig. S4), but this may not be surprising as Mouse Genome Informatics [32] data shows relatively uniform ubiquitin expression across tissues. We speculate on potential roles of DSSs in the evolution and gene expansion of repeat genes (like ubiquitin) below.

### The gene structure of polyubiquitin evolves rapidly

**Fig. 5:**
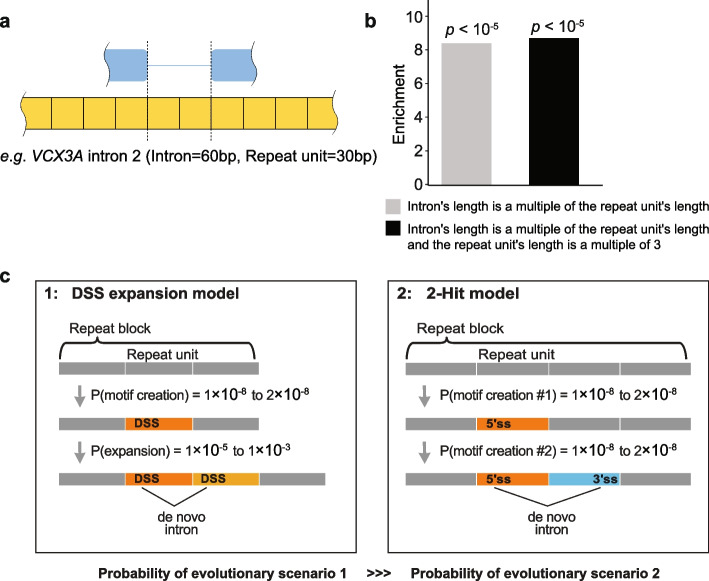
Introns in tandem repeats could suggest an evolutionary mechanism for de novo intron creation. **a** Illustrated example of an intron (blue line) in a tandem repeat (yellow) whose length is a multiple of the repeat unit’s length. **b** Enrichment of introns that splice via DSSs (permutation test, see Methods). **c** Comparison between two models of de novo intron creation in a tandem repeat: the DSS expansion model (1) and the 2-hit model (2). Arrows indicate evolutionary change. Probabilities of motif creation and repeat expansion taken from Conrad et al. [37] and Fan and Chu [38]

Considerable variation in gene architecture and gene family size has been observed in ubiquitin genes across species [33–36]. To better understand introns like *UBC*, we retrieved all introns located in tandem repeat blocks in the human genome. This analysis returned 792 introns embedded in 262 tandem repeats (see Methods, Fig. 5). We did not consider the 603 cases where the repeat was very large relative to the intron (e.g., introns in a gene that maps to a segmental duplication). Instead, we considered all cases where an intron was embedded in tandem repeats of repeat unit length equal to or smaller than the intron. *UBC* is the special case where the intron is a multiple of the repeat unit’s length. We regard these cases as DSS spliced as the same sequence is used as a 5′ss and 3′ss (e.g., Fig. 5a). We were surprised to find an 8.4-fold enrichment for this special case of DSS spliced introns whose length is a multiple of repeat unit length (*n* = 77; *p* < 10^−5^, permutation test, 100,000 trials) (Fig. 5b). Within these special cases, 45/77 had a length that was a multiple of 3 and so their failure to splice would not disrupt the reading frame, representing an 8.7-fold enrichment (*p* < 10^−5^, permutation test, 100,000 trials) (Fig. 5b). As repeat expansions occur 1000 times more frequently than other types of mutations [37–39], we considered the evolutionary scenario of de novo intron formation via expansions of DSS-containing repeat units (Fig. 5c). Most repeat expansions in the coding region of a gene are deleterious; however, in these special cases, loss of fitness could be ameliorated by splicing out insertions in the RNA. In the final section below, we discuss our discovery of polymorphic introns in repeat genes as potential evolutionary intermediates of de novo intron creation.

## Discussion

This study reports an excess of splicing events in the human genome by analyzing the SRA [23] and direct testing in an MPRA. We further confirmed the prevalence of unannotated splice sites by MPRA in *BRCA1*, *BRCA2*, and *LDLR*, revealing 10 times more unannotated splice sites than what was annotated. These findings suggested that splicing occurs more frequently than what was initially indicated by the final annotation. According to recent findings by Wan et al., the splicing of many human introns occurs through a multi-step and recursive process, rather than being removed as a single unit [24]. This may be accomplished by the formation of the spliceosome complex at multiple potential splice sites within each intron, where the appropriate splice site is selected in a stochastic fashion. As a result, the spliceosome makes multiple cuts within introns and the intermediates, eventually leading to the creation of a final spliced mRNA. Many events cannot be placed in a temporal sequence so it is also possible that splicing could continue after the annotated event on the excised lariat.

The accurate recognition of both 5′ and 3′ splice sites is a critical step in the splicing process. However, when both sites are present as in a DSS, the ambiguity in identifying the correct splicing outcome poses a challenge for the spliceosome. In this study, we found evidence of coordination between the 5′ and 3′ splice site recognition processes in DSSs. The synergy provided by combining the two types of sites is seen in DSSs recovered from both MPRAs and SRA data, which can perform the same level of splicing activity with weaker motifs than single splice sites.

Our observations were particularly noteworthy in the *UBC* gene, as shown in Fig. 4, where the DSS on the second subunit serving as the 5′ss had a strong MaxEnt score of 4.3, while the paired DSS on the eighth subunit serving as the 3′ss had a weaker score of − 0.21. Despite the weaker score, the presence of the strong 5′ss may enhance the recognition of the weak 3′ss. This enhancement could occur because U1 snRNP not only binds to the 5′ss but also recruits U2 snRNP and other splicing factors to the adjacent 3′ss [13, 40]. This collaboration between trans-acting factors could compensate for suboptimal cis-acting elements such as weak splice sites. The first study to characterize DSSs suggested that there was a competition between spliceosomal components in recognizing a DSS as a 5′ versus 3′ splice site [22]. This analysis was based on the degree of agreement to 5′ss or 3′ss position weight matrices (PWMs). However, PWMs are built from annotated sites which are underrepresented for DSSs (Fig. 2b), and so, it is possible interactions outside the motif windows enhance spliceosome recruitment.

Analysis of observed events in the Sequence Read Archive revealed a 3.1-fold enrichment of DSSs relative to expectation based on the frequency of single-use AGGT sites, suggesting synergy in recognition of intronic locations. In addition to the splice junctions from the SRA, we found support for the usage of these DSS by mapping lariat reads present in RNA-seq data (Additional file 1: Table S1). Interestingly, these dual-use sites are underrepresented in the annotation, which are the sites required to make the final mRNA. It has been noted that if the spliceosome engages in stochastic splicing it is no longer hard to understand how splice sites are selected [24]. Instead, the question shifts to how splice site selection stops. Here, we report the annotated 5′ and 3′ splice junctions are 2.7- and 4.7-fold underrepresented for DSSs (Fig. 2d). We believe the depletion of DSSs in annotated sites may be one way to stop splicing.

Perhaps the more interesting cases of DSS usage were found in the human *UBC* gene which has 9 ubiquitin-coding units that are linked head-to-tail, separated by DSSs. The intron/exon boundaries precisely align with the protein-coding boundaries. This unique structure allows for the possibility of regulating the amount of ubiquitin by either removing or retaining the coding region through alternative splicing at the DSSs without affecting the reading frame of the transcript. A previous study suggested that increasing cellular ubiquitin levels resulted in a higher amount of intron-retaining *UBC* transcripts in the nucleus, subsequently leading to lower *UBC* gene expression [31]. As we did not find evidence supporting the idea that ubiquitin exerts a negative feedback on itself via transcript isoform regulation, we focused on the potential roles of DSSs in ubiquitin evolution and gene expansion.

The proximity of each DSS to the boundaries of the encoded ubiquitin peptide is reminiscent of the “intron first” evolutionary theory of the origins of splicing [41–43]. The intron first theory suggests the diverse repertoire of modular proteins evolved from exons that were used to encode protein domains. This idea requires an alignment between splice sites and the boundaries of protein domains. In the proteome, evidence for this alignment is controversial. However, in *UBC*, there is a precise alignment between the peptide recognition element of the factor (i.e., DUB) that cleaves polyubiquitin into ubiquitin monomers and the DSS which cleaves the pre-mRNA (Fig. 3). It is relevant that there is a great diversity of repeat numbers in *UBC* genes across species. We found that the degree of expansion ubiquitin subunits in *UBC* homologs varies considerably among organisms–from as few as 3 in Eurasian Red Squirrels to as many as 46 in Eastern Happy fish–with no obvious phylogenetic pattern (Additional file 1: Fig. S5 and Additional file 1: Table S3). We suggest this indicates relaxed selection on the repeat number and/or a high mutation rate. It is likely that both explanations are valid. The repeat unit is a multiple of three, so an expansion will either be removed by splicing or processed to create an extra monomer. The mutation rate for repeat expansions and contractions is known to be high because uneven recombination, gene conversion, or replication slippage occur at frequencies at least 1000-fold higher than substitutions [37–39]. Consistent with expansion being a frequent event, we observed a potential evolutionary intermediate: an additional copy of intron 3 of *the VCX3A* gene appears as an insert polymorphism in the Genome Aggregation Database [44] (ENST00000398729; Additional file 1: Fig. S6, Fig. 5a). To our knowledge, this is the only example of an evolutionary intermediate of de novo intron creation that involves a spliceosomal intron. *VCX3A* is analogous to the *UBC* gene in that it contains 8 tandem repeats with a DSS within the repeat unit. A global search for similar cases returned an 8.4-fold excess of introns in the human genome whose length was a multiple of a surrounding repeat’s length (*n* = 77, *p* < 10^−5^) and therefore have a dual-specific 5′ss and a dual-specific 3′ss. There was also an 8.7-fold excess of introns with the additional constraint that the repeat unit’s length was a multiple of 3 (*n* = 45, *p* < 10^−5^), but this is likely due to selective pressure on repeat expansions to not disrupt protein-coding sequences since there was only a small increase in enrichment (Fig. 5b). It is likely that a DSS-containing repeat expansion is less deleterious as the expanded region can be spliced out and therefore have minimal impact on fitness. Expansions of DSS-containing repeats at nongenic loci could result in de novo gene creation since the mechanism of creation (repeat expansion of a single DSS) is more probable than two separate splice-site creation events, and introns increase gene expression and pol II elongation [45]. Similarly, DSS-containing repeats that are incompletely spliced may give rise to processed pseudogenes that contain introns. It is possible that cDNA insert via retrotransposition and because of the presence of functional introns in *UBC* (or other) mRNA, the retrotransposed element is more likely to be expressed at its new loci. This idea is supported by the great diversity of ubiquitin-containing genes found in many genomes (Additional file 1: Fig. S5).

## Conclusions

Our study reveals an abundance of unannotated splice sites, suggesting increased chances of stochastic splicing at various potential sites within introns. By identifying DSSs and their extensive usage in the SRA, we have expanded our understanding of the mechanisms involved in splicing regulation. Additionally, our investigation of the polyubiquitin (*UBC*) gene, where DSSs delineate tandem repeats of ubiquitin coding monomers, highlights the intricate nature of splicing and its impact on generating variable numbers of ubiquitin monomers. Furthermore, our genome-wide analysis suggests that DSSs embedded within tandem repeats serve as a mechanism for gene evolution, potentially driving the diversification and adaptation of genes over time.

## Methods

### Sequence read archive data

We used the exon-exon junction dataset “intropolis” as a representative sample of the full population of human introns. This dataset was produced from the sequencing data of 21,504 human RNA-seq samples that were publicly available in the Sequence Read Archive (SRA) [23]. We extracted the 4-bp sequences for the 5′ splice sites (2 bp upstream of the 5′ss and the 5′ss itself) and 3′ splice sites (the 3′ss itself and 2 bp downstream of the 3′ss) listed in intropolis. Our focus was solely on dual-specific splice sites (DSSs) with an AGGT motif. A site that includes the AGGT motif and demonstrates the capability to function as both a 5′ss and a 3′ss, each with a minimum read count of 10, is identified as a DSS.

### Oligonucleotide library design and synthesis

Three large genes *BRCA1*, *BRCA2*, and *LDLR* were used in the massively parallel reporter assay (MPRA). DNA oligonucleotides (oligos) were designed such that each gene was tiled in 150 nucleotide windows with a step-size of 20 nucleotides. The gene sequences were based on the GENCODE v32 reference genome and basic gene annotation [46]. This resulted in 6290 oligos for *BRCA1*, 4251 for *BRCA2*, and 2215 for *LDLR* (12,756 oligos in total). Each oligo was flanked by forward and reverse common primer sequences, producing a 230-nucleotide oligo. Finally, the three oligo libraries for these three genes were synthesized by Agilent Technologies.

### Minigene construction

The three oligo libraries were first amplified by 20 cycles of PCR (Q5 Hot Start High-Fidelity DNA Polymerase, NEB), and then separately incorporated into two types of minigene report constructs: a 5′ss-testing minigene and a 3′ss-testing minigene. The former consists of, in order from 5′ to 3′, a cytomegalovirus (CMV) promoter, a 230-mer sequence from one of three oligo libraries, exon 16 of the *ACTN1* gene with parts of its upstream intron 15, and a bGH polyA sequence. The *ACTN1* 3′ss tests for potential 5′ splice sites. The 3′ss-testing minigene consists of a CMV promoter, an adenovirus pHMS81 exon (AD81 exon) with part of its downstream intron, a 230-mer sequence from one of three oligo libraries, and a bGH polyA sequence. The AD81 exon’s 5′ss tests for potential 3′ splice sites. The minigene fragments upstream and downstream of the oligo libraries were extended to include primer sequence overlap, and full minigene libraries were subsequently assembled by overlapping PCR. The minigene libraries were then pooled together in equimolar amounts, resulting in an input library with all minigene reporter constructs.

### Minigene library transfection and input and output library sequencing

The resulting minigene input libraries were transfected into HEK293 cells obtained from the American Type Culture Collection (ATCC CRL-316) in three cell culture replicates using Lipofectamine 3000 (Invitrogen) in a 6-well plate. Twenty-four hours after transfection, RAN was extracted by RNeasy Mini Kit (Qiagen), followed by DNase treatment (Invitrogen). Random 9-mers were used to generate cDNA with SuperScript IV Reverse Transcriptase (Invitrogen) followed by PCR (GoTaq, Promega), resulting in output libraries of transcripts with all potential 5′ or 3′ splice sites. Input and output libraries were sequenced using Illumina HiSeq 2 × 150 bp. Cultured cells were authenticated using short-tandem-repeat profiling and were periodically tested for mycoplasma contamination.

### Enrichment score calculation

Input and output library reads were trimmed to endogenous sequence using SeqKit amplicon [47], and then the endogenous sequence was aligned to the gene sequence using STAR [48] in unspliced, end-to-end, unique alignment mode. Each aligned input read corresponded to an oligo tiling the gene of interest. Each aligned output read corresponded to a pair of splice junctions and tiling oligo. The enrichment score was the read coverage in the output library normalized by the read coverage in the input library, and the resulting value was log10-transformed. Each splice junction had to have at least 20 reads in each replicate’s output library to be included.

### Lariat mapping of unannotated dual splice sites

Our custom lariat mapping pipeline was implemented based on the method described in Pineda and Bradley 2018 [49]. First, reads are filtered out if they contain > 5% ambiguous characters. Then, reads are mapped to the genome, and aligned reads are discarded. A mapping index is then created based on the unaligned reads, and a Fasta file containing the sequence of the first 20 nt of each annotated intron in the transcriptome is mapped to the unaligned reads. In order to capture splicing events from unannotated sites, we also added the 20 nt 5′ss sequences from unannotated DSSs with > 10 supporting junction reads in the Sequence Read Archive. Reads are then identified where only one 5′ss maps to them and the alignment has no mismatches or indels. These reads are then trimmed of the sequence from the start of the 5′ss alignment to the end of the read, and reads with a trimmed length of < 20 nt are filtered out. The remaining trimmed reads are mapped to an index built from the last 250 nt of every annotated intron. The trimmed read alignments are then filtered to only consider those with <  = 5 mismatches, <  = 10% mismatch rate, and no more than one indel of <  = 3 nt. Then, for each trimmed read, the highest scoring alignment was chosen after restricting to alignments in the same gene as the 5′ss alignment and those with the expected inverted mapping order of the 5′ and 3′ segments. The end of this highest scoring alignment is then taken to be the branchpoint of the lariat the read is derived from.

Previously, we generated a DBR1 knockout cell line from HEK293T cells via CRISPR (under review at Nature Communications). Due to the high level of lariats in this cell line, we processed RNA-seq samples from it with the pipeline above in order to test for the presence of lariat reads originating from unannotated dual splice sites. Table S1 (Additional File 1) contains information about the lariat reads that were recovered from these DSSs.

### Plasmid transfections and PCR amplification

HEK293 cells were seeded the day before transfection into a 6-well plate in order to reach ~ 60–70% confluence at the time of transfection, and each well was transfected with 0.5 μg, 2 μg, or 5 μg of pRK5-HA-ubiquitin-WT plasmid (Addgene plasmid # 17,608) [50] using Lipofectamine 3000 (Invitrogen) transfection reagent. After 48 h, cells were harvested for RNA and protein analyses. PCR amplification was performed using GoTaq Master Mix (Promega). The primer sequences for *UBC* amplification were as follows: forward (5′-3′): TGGGTCGCAGTTCTTGTTTG; reverse (5′-3′): GTGCAATGAAATTTGTTGAAACCTTAAAAGGGG. Validation of PCR products was done using the QIAxcel ScreenGel Software.

### Western blot

Cell lysates were prepared with cOmplete Lysis M buffer, EDTA-free according to the manufacturer’s protocol (Roche). Protein samples were separated on 4–20% Mini-PROTEAN gels (Bio-Rad) and transferred to a polyvinylidene difluoride (PVDF) membrane. The blot was probed with rabbit monoclonal HA-Tag (C29F4) antibody (Cell Signaling Technology, #3724) and mouse monoclonal beta-actin antibody (Abcam, ab8226) and then was imaged by the LiCor Odyssey System.

### Ubiquitin annotation

The 9 ubiquitin subunits in *UBC* and 3 ubiquitin subunits in *UBB* were aligned in SnapGene using MUSCLE (v3.8.1551), and the resulting consensus sequence was ATGCAGATCTTCGTGAAGACCCTGACTGGTAAGACCATCACCCTCGAGGTGGAGCCCAGTGACACCATCGAGAATGTCAAGGCAAAGATCCAAGANAAGGAAGGCATCCCTCCTGACCAGCAGAGGTTGATCTTTGCNGGNAAACAGCTGGAAGATGGNCGCACCCTGTCTGACTACAACATCCAGAAAGAGTCCACCCTGCACCTGGTGCTCCGTCTNAGAGGTGGG. This sequence was used to identify ubiquitin subunits in *UBC* orthologs identified from Ensembl release 109, assembly GRCh38.p13 [30], excluding orthologs with Target %id < 50% or Query %id < 50%.

Each gene’s coding sequence was aligned to the consensus sequence in pairwise local alignments using the EMBOSS matcher application (v6.6.0.0) [51]. We ran matcher with the default scoring matrix, open gap penalty, and gap extension penalty for DNA. For each gene, the number of alternative alignments was set equal to the length of the coding sequence divided by the length of the consensus sequence (228 bp), rounded up. Alignments with identity < 50%, similarity < 50%, or length < 171 bp were discarded. No overlapping alignments remained after filtering.

A phylogenetic tree of the orthologs was built using the R package rotl, which queries the Open Tree of Life taxonomy v3.3 draft 1 [52, 53].

### Mapping gnomAD inserts to annotated introns

Variant Call Format (VCF) files covering variants in chromosomes 1–22 and X from Genome Aggregation Database (gnomAD) v2.1.1 were obtained from Google Cloud Public Datasets at gs://gcp-public-data–gnomad. VCF files covering chromosomes 1–22, X, and Y from gnomAD v3.1.1 were obtained from the same public dataset [44]. Insert variants that passed all gnomAD filters and were at least 50 bp long were extracted from the gnomAD v2.1.1 and v3.1.1 VCF files.

Annotations for hg19 and hg38 introns were obtained from the University of California Santa Cruz (UCSC) Genome Browser Database tables wgEncodeGencodeCompV19 and wgEncodeGencodeCompV41 tables, respectively, using Table Browser tool [54]. The hg19 and hg38 introns’ DNA sequences were obtained from the primary assemblies of GENCODE releases 19 and 41, respectively [46]. gnomAD v2.1.1 and v3.1.1 inserts were mapped to hg19 and hg38 introns, respectively, using bowtie2 in end-to-end mode with the “–very-sensitive” argument [55]. Successful alignments were then filtered, retaining alignments wherein the intron’s length was within 5 bp of the mapped insert’s length.

We identified an insert from gnomAD v3.1.1 in *VCX3A* with a sequence that exactly matched *VCX3A*’s third intron (Additional file 1: Fig. S6). The ubiquitin consensus sequence (see the “Ubiquitin annotation” section) was also mapped to hg19 and hg38 introns, but none of the resulting alignments passed filtering.

### Intron-tandem repeat intersection analysis

Annotations for introns and tandem repeat blocks in hg38 were obtained from the UCSC Genome Browser Database tables knownGene and simpleRepeats, respectively, using the Table Browser tool [54]. Introns which were in different genes or transcripts but had the same genomic coordinates were collapsed into one intron. Tandem repeats with the same genomic coordinates were also collapsed. The consensus length reported for each repeat was treated as the length of its repeat unit length, using the smallest consensus length among identical repeats.

We analyzed these annotations and identified 792 introns within tandem repeat in the human genome (Additional file 1: Table S4). Due to overlapping annotations, some of these introns were matched with multiple repeat blocks. There were 77 introns whose length was a multiple of the repeat unit’s length in at least one of the tandem repeats they fell within, and for 45 of those introns, the repeat unit’s length was a multiple of 3 (Additional file 1: Fig. S7). We tested the statistical significance of these subsets in a permutation test of 100,000 trials by randomizing the intron-repeat pairings, counting the number of introns that fell into each subset, and then comparing the counts to our results. We excluded intron-repeat pairings where the intron was shorter than the repeat unit length from these trials since we were only interested in cases where the intron was at least as long as the repeat unit. The mean count over all trials for each subset was 9.1786 (8.4-fold enrichment) and 5.17901 (8.7-fold enrichment), respectively. In both subsets, no trials produced a count equal to or greater than the count we observed.   

### Supplementary Information

**Additional file 1: Figure S1.** Differences between recursive splice sites (RSSs) and dual-specific splice sites (DSSs). **Figure S2.** Dual-specific splice sites support splicing activity with weaker substrate sequences. **Figure S3.** The effect of overexpressing HA-tagged ubiquitin in HEK293 cells. **Figure S4.** RT-PCR analysis of the splicing pattern of mouse *UBC* genes from brain, liver, and muscle tissues. **Figure S5.** Evolution of polyubiquitin gene family. **Figure S6.** Sequence of insert variant X-6533768-T-TCTCGCTCTCCTGACTCAGTGGTTCCTCCACCTGGCTCTCCTGACTCAGTGGTTCTTCCAC in *VCX3A*, which matches intron 3 of ENST00000398729. **Figure S7.** Distribution of splice site sequences of introns whose length is a multiple of a surrounding tandem repeat’s unit length. **Table S1.** Lariat reads recovered from the 5′ splice sites of unannotated dual splice sites. **Table S2.** Two isoform sequences in Fig. 4. **Table S3.** Ubiquitin subunit counts of *UBC* orthologs. **Table S4.** Introns in tandem repeats.

**Additional file 2:** Review history.

## Data Availability

The code and data used in this study are available on Zenodo: https://zenodo.org/doi/https://doi.org/10.5281/zenodo.10359773 [56] and https://zenodo.org/doi/https://doi.org/10.5281/zenodo.8101789 [57]. The custom UCSC track for our MRRA is available at https://genome.ucsc.edu/s/stephenrongbrown/All3ss_BRCA1_BRCA2_LDLR_experiments_v2.1. The raw RNA-seq data, collected from a HEK293T-derived DBR1 knockout cell line and utilized for the identification of unannotated DSSs, is available on the Gene Expression Omnibus (GEO) under accession number GSE195586: https://www.ncbi.nlm.nih.gov/geo/query/acc.cgi?acc=GSE195586 [58].
