# Supplementary material for: The unusual gene architecture of polyubiquitin is created by dual-specific splice sites
Source: Genome Biol. 2024 Jan 24;25:33. doi: 10.1186/s13059-023-03157-8 (PMC10809524; doi:10.1186/s13059-023-03157-8)
Supplement: Supplementary file 1 — Additional file 1: Figure S1. Differences between recursive splice sites (RSSs) and dual-specific splice sites (DSSs). Figure S2. Dual-specific splice sites support splicing activity with weaker substrate sequences. Figure S3. The effect of overexpressing HA-tagged ubiquitin in HEK293 cells. Figure S4. RT-PCR analysis of the splicing pattern of mouse UBC genes from brain, liver, and muscle tissues. Figure S5. Evolution of polyubiquitin gene family. Figure S6. Sequence of insert variant X-6533768-T-TCTCGCTCTCCTGACTCAGTGGTTCCTCCACCTGGCTCTCCTGACTCAGTGGTTCTTCCAC in VCX3A, which matches intron 3 of ENST00000398729. Figure S7. Distribution of splice site sequences of introns whose length is a multiple of a surrounding tandem repeat’s unit length. Table S1. Lariat reads recovered from the 5′ splice sites of unannotated dual splice sites. Table S2. Two isoform sequences in Fig. 4. Table S3. Ubiquitin subunit counts of UBC orthologs. Table S4. Introns in tandem repeats. [file 13059_2023_3157_MOESM1_ESM.docx]

**Figure S1**. **Differences between recursive splice sites (RSSs) and dual-specific splice sites (DSSs).** RSSs function as a 3'ss AND THEN a 5'ss or in reverse order (i.e. a 5'ss AND THEN a 3'ss), and DSSs function as a 5'ss OR a 3'ss. While a DSS is a sequence that can function as a 5'ss or a 3'ss, this is not necessarily true for an RSS. The sequence of a recursive site changes between the first and second splice so it is possible for a recursive splice located in an intron to only function as a 3'ss, initially, but then gain 5'ss function after the introduction of new upstream context.

**Figure S2**. **Dual-specific splice sites support splicing activity with weaker substrate sequences.** Splice sites present in SRA data were stratified based on their level of junction read support and their status as a DSS or single splice site. At each threshold of splicing activity, the distribution of MaxEnt scores is shown for 5' splice sites (top) and 3' splice sites (bottom).

**Figure S3.** **The effect of overexpressing HA-tagged ubiquitin in HEK293 cells.** (**a**) Cells were transfected with increasing amounts of the ubiquitin expression vector, and the levels of HA-tagged ubiquitin were detected by Western blot analysis. (**b**) RT-PCR analysis of the effect of exogenous ubiquitin on the splicing pattern of *UBC*.

**Figure S4.** **RT-PCR analysis of the splicing pattern of mouse *UBC* genes from brain, liver, and muscle tissues.**

**Figure S5.** **Evolution of polyubiquitin gene family**. (**a**) Phylogenetic tree of *UBC* orthologs. (**b**) Histogram of *UBC* ortholog subunit counts.

**Figure S6.** **Sequence of insert variant X-6533768-T-TCTCGCTCTCCTGACTCAGTGGTTCCTCCACCTGGCTCTCCTGACTCAGTGGTTCTTCCAC in *VCX3A*, which matches intron 3 of ENST00000398729.** The AGGTs in repeats 2 and 4 are used as a 5' and 3' splice sites, respectively, in ENST00000398729. Yellow box = 30-base-pair tandem repeat; Red bar = AGGT motif.

**Figure S7.** **Distribution of splice site sequences of introns whose length is a multiple of a surrounding tandem repeat’s unit length.**

**Table S1. Lariat reads recovered from the 5' splice sites of unannotated dual splice sites.** RNA-seq data was processed using a custom lariat mapping pipeline in order to detect evidence of splicing from the 5' splice sites of DSSs supported by >10 reads in the Sequence Read Archive.

| gene | chrom | strand | fivep_pos | bp_pos | genomic_bp_context | read_count |
| --- | --- | --- | --- | --- | --- | --- |
| SLC35D1 | chr1 | - | 67053289 | 67053020 | GTTCTGATCT | 1 |
| ENSG00000260246 | chr1 | - | 109693562 | 109693523 | GGGAGGGGAT | 1 |
| TIMM23B-AGAP6 | chr10 | + | 49973078 | 49987139 | GATCTGATGG | 1 |
| MBL1P | chr10 | + | 79920317 | 79920925 | CTGGGTCATC | 1 |
| DDB1 | chr11 | - | 61310333 | 61309869 | ATTGTGGGCA | 1 |
| B3GAT3 | chr11 | - | 62617511 | 62617371 | TTTCCTTGTG | 1 |
| PHB2 | chr12 | - | 6967392 | 6967295 | GACAGGGCCA | 1 |
| RPL41 | chr12 | + | 56116829 | 56117133 | CTTCATCCCT | 1 |
| HERC2 | chr15 | - | 28214531 | 28214370 | GAAGAGCGGC | 1 |
| MAN2C1 | chr15 | - | 75368094 | 75367654 | GCCTATCACA | 1 |
| JMJD7-PLA2G4B | chr15 | + | 41837374 | 41840131 | TAGCAGGTCT | 1 |
| ANKS3 | chr16 | - | 4707021 | 4705286 | ACTGAACACA | 2 |
| NPIPB4 | chr16 | - | 21857657 | 21857454 | TTTTCCACTC | 1 |
| MVP-DT | chr16 | - | 29810444 | 29810292 | GGGAGAGGAG | 1 |
| PDXDC2P-NPIPB14P | chr16 | - | 69996467 | 69996258 | ACTCATTCAA | 1 |
| URAHP | chr16 | - | 90044056 | 90044004 | TCCTGGCCTC | 1 |
| PIGQ | chr16 | + | 580565 | 580725 | GCTCAGGGTG | 1 |
| SRRM2 | chr16 | + | 2770227 | 2770327 | TGCTATTGGG | 2 |
| RNF40 | chr16 | + | 30762051 | 30762447 | TTCCCACATC | 1 |
| MYO1C | chr17 | - | 1471991 | 1471359 | TGGGAGCCGC | 1 |
| RPL26 | chr17 | - | 8377781 | 8377725 | TTAAAGCTTG | 1 |
| SLC25A39 | chr17 | - | 44321292 | 44320749 | ATCAGTGCAT | 1 |
| P4HB | chr17 | - | 81859298 | 81855537 | ACGGGCCCGG | 1 |
| NARF | chr17 | + | 82484349 | 82484776 | CAGCATTCAT | 1 |
| FN3KRP | chr17 | + | 82719102 | 82722774 | ACTAATTTCC | 1 |
| PIP5K1C | chr19 | - | 3651820 | 3648733 | GCTGATGGTA | 1 |
| MICOS13 | chr19 | - | 5679531 | 5679466 | TCCTCCTCCC | 1 |
| ENSG00000267512 | chr19 | - | 13140181 | 13140061 | CTGGTCCCAT | 1 |
| SMIM7 | chr19 | - | 16652063 | 16647290 | CCTCACAGAC | 1 |
| ENSG00000269292 | chr19 | - | 46609555 | 46609452 | GGCAGAGGCT | 1 |
| THOP1 | chr19 | + | 2807584 | 2808223 | CCTGACGCTG | 2 |
| PIN1 | chr19 | + | 9849089 | 9849357 | ATTGGGGGCC | 1 |
| UXS1 | chr2 | - | 106094229 | 106094208 | CGATGCCCTT | 1 |
| EMILIN1 | chr2 | + | 27084999 | 27085073 | TCGGGCAGCC | 2 |
| ZNG1B | chr2 | + | 113437845 | 113444934 | ATTAACTGTA | 1 |
| RPL3 | chr22 | - | 39314277 | 39314276 | CAGGTCAGCA | 18 |
| RRP7A | chr22 | - | 42515258 | 42514806 | AAGCCCCGTT | 4 |
| TUBGCP6 | chr22 | - | 50221273 | 50220039 | TGCCCTCGTG | 3 |
| PLXNB2 | chr22 | - | 50286446 | 50286313 | ACCTTGACAC | 2 |
| ACO2 | chr22 | + | 41507606 | 41507767 | GCCACCCTTC | 2 |
| TMEM44 | chr3 | - | 194629645 | 194628546 | ACTCACAGTT | 1 |
| SEMA3F | chr3 | + | 50184795 | 50185419 | ACTGAGGCCC | 1 |
| H1-10-AS1 | chr3 | + | 129315642 | 129315715 | CCGTTGCGCT | 1 |
| ENSG00000228028 | chr3 | + | 196250662 | 196251237 | CTGAGGCTGA | 1 |
| RPS14 | chr5 | - | 150447672 | 150446985 | GAAATGACCC | 2 |
| DUSP1 | chr5 | - | 172770151 | 172769828 | GAGCAAGCTT | 4 |
| RACK1 | chr5 | - | 181237223 | 181237071 | AGCCTGACCT | 1 |
| MATR3 | chr5 | + | 139325340 | 139325422 | GATGGTTTGG | 1 |
| HSPA1A | chr6 | + | 31816068 | 31816127 | TGCTGACCAA | 1 |
| KMT2C | chr7 | - | 152251970 | 152250989 | GCTAAAGAAT | 2 |
| SUN1 | chr7 | + | 882380 | 886033 | GGCTCACGGT | 1 |
| CARD9 | chr9 | - | 136367752 | 136367289 | CCTCACAGCC | 2 |
| ANAPC2 | chr9 | - | 137176001 | 137175853 | GCTGACCCTG | 1 |
| RABL6 | chr9 | + | 136837980 | 136839287 | CCTGGCCAGG | 1 |
| GDI1 | chrX | + | 154438564 | 154440352 | AATCAGGGGT | 1 |

**Table S2. Two isoform sequences in Figure 4.** Ubiquitin unit 1 is highlighted in green, ubiquitin unit 2 in yellow, ubiquitin unit 8 in blue and ubiquitin unit 9 in purple.

| Isoform | Sequence |
| --- | --- |
| Upper isoform in Figure 4 | TGGGTCGCAGTTCTTGTTTGTGGATCGCTGTGATCGTCACTTGACAATGCAGATCTTCGTGAAGACTCTGACTGGTAAGACCATCACCCTCGAGGTTGAGCCCAGTGACACCATCGAGAATGTCAAGGCAAAGATCCAAGATAAGGAAGGCATCCCTCCTGATCAGCAGAGGCTGATCTTTGCTGGAAAACAGCTGGAAGATGGGCGCACCCTGTCTGACTACAACATCCAGAAAGAGTCCACCCTGCACCTGGTGCTCCGTCTCAGAGGTGGGATGCAAATCTTCGTGAAGACACTCACTGGCAAGACCATCACCCTTGAGGTGGAGCCCAGTGACACCATCGAGAACGTCAAAGCAAAGATCCAGGACAAGGAAGGCATTCCTCCTGACCAGCAGAGGTTGATCTTTGCTGGGAAACAGCTGGAAGATGGACGCACCCTGTCTGACTACAACATCCAGAAAGAGTCCACCCTGCACCTGGTGCTCCGTCTTAGAGGTGGGATGCAGATCTTCGTGAAGACCCTGACTGGTAAGACCATCACTCTCGAAGTGGAGCCGAGTGACACCATTGAGAATGTCAAGACAAAGATCCAAGACAAGGAAGGCATCCCTCCTGACCAGCAGAGGTTGATCTTTGCTGGGAAACAGCTGGAAGATGGACGCACCCTGTCTGACTACAACATCCAGAAAGAGTCCACCCTGCACCTGGTGCTCCGTCTCAGAGGTGGGATGCAGATCTTCGTGAAGACCCTGACTGGTAAGACCATCACCCTCGAGGTGGAGCCCAGTGACACCATCGAGAATGTCAAGGCAAAGATCCAAGATAAGGAAGGCATCCCTCCTGATCAGCAGAGGTTGATCTTTGCTGGGAAACAGCTGGAAGATGGACGCACCCTGTCCGACTACAACATCCAGAAAGAGTCCACTCTGCACTTGGTCCTGCGCTTGAGGGGGGGTGTCTAAGTTTCCCCTTTTAAGGTTTCAACAAATTTCATTGCAC |
| Lower isoform in Figure 4 | TGGGTCGCAGTTCTTGTTTGTGGATCGCTGTGATCGTCACTTGACAATGCAGATCTTCGTGAAGACTCTGACTGGTAAGACCATCACCCTCGAGGTTGAGCCCAGTGACACCATCGAGAATGTCAAGGCAAAGATCCAAGATAAGGAAGGCATCCCTCCTGATCAGCAGAGGCTGATCTTTGCTGGAAAACAGCTGGAAGATGGGCGCACCCTGTCTGACTACAACATCCAGAAAGAGTCCACCCTGCACCTGGTGCTCCGTCTCAGAGGTGGGATGCAAATCTTCGTGAAGACACTCACTGGCAAGACCATCACCCTTGAGGTGGAGCCCAGTGACACCATCGAGAACGTCAAAGCAAAGATCCAGGACAAGGAAGGCATTCCTCCTGACCAGCAGAGGTTGATCTTTGCTGGGAAACAGCTGGAAGATGGACGCACCCTGTCTGACTACAACATCCAGAAAGAGTCCACCCTGCACCTGGTGCTCCGTCTTAGAGGTGGGATGCAGATCTTCGTGAAGACCCTGACTGGTAAGACCATCACCCTCGAGGTGGAGCCCAGTGACACCATCGAGAATGTCAAGGCAAAGATCCAAGATAAGGAAGGCATCCCTCCTGATCAGCAGAGGTTGATCTTTGCTGGGAAACAGCTGGAAGATGGACGCACCCTGTCCGACTACAACATCCAGAAAGAGTCCACTCTGCACTTGGTCCTGCGCTTGAGGGGGGGTGTCTAAGTTTCCCCTTTTAAGGTTTCAACAAATTTCATTGCAC |

**Table S3. Ubiquitin subunit counts of *UBC* orthologs.**

| **Species, Common Name** | **Species, Scientific Name** | **Protein ID** | **Ubiquitin Subunits** |
| --- | --- | --- | --- |
| C. elegans | *Caenorhabditis elegans* | F25B5.4.2 | 11 |
| Fruit fly | *Drosophila melanogaster* | FBpp0073035 | 10 |
| Solitary sea squirt | *Ciona savignyi* | ENSCSAVP00000012392 | 9 |
| Atlantic cod | *Gadus morhua* | ENSGMOP00000050001 | 10 |
| Pinecone soldierfish | *Myripristis murdjan* | ENSMMDP00005044403 | 9 |
| Tiger tail seahorse | *Hippocampus comes* | ENSHCOP00000009639 | 13 |
| Pike-perch | *Sander lucioperca* | ENSSLUP00000021246 | 8 |
| Stickleback | *Gasterosteus aculeatus* | ENSGACP00000026768 | 8 |
| Lumpfish | *Cyclopterus lumpus* | ENSCLMP00005010920 | 10 |
| Ballan wrasse | *Labrus bergylta* | ENSLBEP00000021236 | 10 |
| Gilthead seabream | *Sparus aurata* | ENSSAUP00010062031 | 11 |
| Fugu | *Takifugu rubripes* | ENSTRUP00000057853 | 4 |
| European seabass | *Dicentrarchus labrax* | ENSDLAP00005067172 | 7 |
| Zig-zag eel | *Mastacembelus armatus* | ENSMAMP00000011057 | 9 |
| Climbing perch | *Anabas testudineus* | ENSATEP00000008835 | 9 |
| Siamese fighting fish | *Betta splendens* | ENSBSLP00000037601 | 9 |
| Greater amberjack | *Seriola dumerili* | ENSSDUP00000023286 | 9 |
| Yellowtail amberjack | *Seriola lalandi dorsalis* | ENSSLDP00000024700 | 8 |
| Turbot | *Scophthalmus maximus* | ENSSMAP00000000429 | 11 |
| Japanese medaka HdrR | *Oryzias latipes* | ENSORLP00000015065 | 10 |
| Platyfish | *Xiphophorus maculatus* | ENSXMAP00000023177 | 11 |
| Eastern happy | *Astatotilapia calliptera* | ENSACLP00000009605 | 9 |
| Eastern happy | *Astatotilapia calliptera* | ENSACLP00000014166 | 46 |
| Atlantic salmon | *Salmo salar* | ENSSSAP00000008699 | 7 |
| Rainbow trout | *Oncorhynchus mykiss* | ENSOMYP00000115309 | 10 |
| Zebrafish | *Danio Rerio* | ENSDARP00000139647 | 7 |
| Golden-line barbel | *Sinocyclocheilus grahami* | ENSSGRP00000030473 | 9 |
| Goldfish | *Carassius auratus* | ENSCARP00000108608 | 8 |
| Tropical clawed frog | *Xenopus tropicalis* | ENSXETP00000104973 | 8 |
| Green anole | *Anolis carolinensis* | ENSACAP00000041232 | 8 |
| Goodes thornscrub tortoise | *Gopherus evgoodei* | ENSGEVP00005012653 | 10 |
| Opossum | *Monodelphis domestica* | ENSMODP00000020484 | 10 |
| Koala | *Phascolarctos cinereus* | ENSPCIP00000043051 | 9 |
| Hyrax | *Procavia capensis* | ENSPCAP00000008325 | 6 |
| Greater horseshoe bat | *Rhinolophus ferrumequinum* | ENSRFEP00010028825 | 8 |
| Pig | *Sus scrofa* | ENSSSCP00000062692 | 11 |
| Cow | *Bos taurus* | ENSBTAP00000053003 | 9 |
| Sheep | *Ovis aries* | ENSOARP00020005495 | 10 |
| Goat | *Capra hircus* | ENSCHIP00000014262 | 8 |
| Sumatran orangutan | *Pongo abelii* | ENSPPYP00000005811 | 8 |
| Chimpanzee | *Pan troglodytes* | ENSPTRP00000050925 | 9 |
| Human | *Homo sapiens* | ENSP00000344818.5 | 9 |
| Eurasian red squirrel | *Sciurus vulgaris* | ENSSVLP00005023449 | 3 |
| Upper Galilee mountains blind mole rat | *Nannospalax galili* | ENSNGAP00000021386 | 6 |
| Rat | *Rattus norvegicus* | ENSRNOP00000074688 | 11 |

**Table S4. Introns in tandem repeats**. “Intron size” refers to whether the intron is larger than, smaller than, or equal in size to the tandem repeat’s repeat unit.

| **Introns in Tandem Repeats** | | | | | | | | |
| --- | --- | --- | --- | --- | --- | --- | --- | --- |
| **Chromosome** | **Gene** | **Intron**  **start** | **Intron**  **end** | **Intron**  **length** | **Repeat**  **start** | **Repeat**  **end** | **Repeat unit length** | **Intron size** |
| 1 | SLC16A1-AS1 | 112,996,604 | 112,996,840 | 237 | 112,996,331 | 112,996,946 | 328 | Smaller |
| 1 | NBPF26 | 120,824,147 | 120,824,865 | 719 | 120,823,471 | 120,828,299 | 1,540 | Smaller |
| 1 | NBPF15 | 144,423,976 | 144,424,689 | 714 | 144,423,965 | 144,428,501 | 1,552 | Smaller |
| 1 | NBPF15 | 144,424,863 | 144,425,516 | 654 | 144,423,965 | 144,428,501 | 1,552 | Smaller |
| 1 | NBPF15 | 144,425,569 | 144,426,277 | 709 | 144,423,965 | 144,428,501 | 1,552 | Smaller |
| 1 | NBPF15 | 144,426,451 | 144,427,046 | 596 | 144,423,965 | 144,428,501 | 1,552 | Smaller |
| 1 | NBPF15 | 144,427,099 | 144,427,817 | 719 | 144,423,965 | 144,428,501 | 1,552 | Smaller |
| 1 | NBPF20 | 145,292,490 | 145,293,203 | 714 | 145,292,479 | 145,393,437 | 1,545 | Smaller |
| 1 | NBPF20 | 145,293,377 | 145,294,020 | 644 | 145,292,479 | 145,393,437 | 1,545 | Smaller |
| 1 | NBPF20 | 145,294,073 | 145,294,781 | 709 | 145,292,479 | 145,393,437 | 1,545 | Smaller |
| 1 | NBPF20 | 145,294,955 | 145,295,566 | 612 | 145,292,479 | 145,393,437 | 1,545 | Smaller |
| 1 | NBPF20 | 145,295,619 | 145,296,337 | 719 | 145,292,479 | 145,393,437 | 1,545 | Smaller |
| 1 | NBPF20 | 145,296,511 | 145,297,138 | 628 | 145,292,479 | 145,393,437 | 1,545 | Smaller |
| 1 | NBPF20 | 145,297,248 | 145,297,961 | 714 | 145,292,479 | 145,393,437 | 1,545 | Smaller |
| 1 | NBPF20 | 145,298,135 | 145,298,786 | 652 | 145,292,479 | 145,393,437 | 1,545 | Smaller |
| 1 | NBPF20 | 145,298,839 | 145,299,547 | 709 | 145,292,479 | 145,393,437 | 1,545 | Smaller |
| 1 | NBPF20 | 145,299,721 | 145,300,328 | 608 | 145,292,479 | 145,393,437 | 1,545 | Smaller |
| 1 | NBPF20 | 145,300,381 | 145,301,099 | 719 | 145,292,479 | 145,393,437 | 1,545 | Smaller |
| 1 | NBPF20 | 145,301,273 | 145,301,900 | 628 | 145,292,479 | 145,393,437 | 1,545 | Smaller |
| 1 | NBPF20 | 145,302,010 | 145,302,723 | 714 | 145,292,479 | 145,393,437 | 1,545 | Smaller |
| 1 | NBPF20 | 145,302,897 | 145,303,532 | 636 | 145,292,479 | 145,393,437 | 1,545 | Smaller |
| 1 | NBPF20 | 145,303,585 | 145,304,293 | 709 | 145,292,479 | 145,393,437 | 1,545 | Smaller |
| 1 | NBPF20 | 145,304,467 | 145,305,069 | 603 | 145,292,479 | 145,393,437 | 1,545 | Smaller |
| 1 | NBPF20 | 145,305,179 | 145,305,892 | 714 | 145,292,479 | 145,393,437 | 1,545 | Smaller |
| 1 | NBPF20 | 145,306,066 | 145,306,715 | 650 | 145,292,479 | 145,393,437 | 1,545 | Smaller |
| 1 | NBPF20 | 145,306,768 | 145,307,476 | 709 | 145,292,479 | 145,393,437 | 1,545 | Smaller |
| 1 | NBPF20 | 145,307,650 | 145,308,252 | 603 | 145,292,479 | 145,393,437 | 1,545 | Smaller |
| 1 | NBPF20 | 145,308,362 | 145,309,075 | 714 | 145,292,479 | 145,393,437 | 1,545 | Smaller |
| 1 | NBPF20 | 145,309,249 | 145,309,896 | 648 | 145,292,479 | 145,393,437 | 1,545 | Smaller |
| 1 | NBPF20 | 145,309,949 | 145,310,657 | 709 | 145,292,479 | 145,393,437 | 1,545 | Smaller |
| 1 | NBPF20 | 145,310,831 | 145,311,436 | 606 | 145,292,479 | 145,393,437 | 1,545 | Smaller |
| 1 | NBPF20 | 145,311,489 | 145,312,207 | 719 | 145,292,479 | 145,393,437 | 1,545 | Smaller |
| 1 | NBPF20 | 145,312,381 | 145,313,008 | 628 | 145,292,479 | 145,393,437 | 1,545 | Smaller |
| 1 | NBPF20 | 145,313,118 | 145,313,831 | 714 | 145,292,479 | 145,393,437 | 1,545 | Smaller |
| 1 | NBPF20 | 145,314,005 | 145,314,664 | 660 | 145,292,479 | 145,393,437 | 1,545 | Smaller |
| 1 | NBPF20 | 145,314,717 | 145,315,425 | 709 | 145,292,479 | 145,393,437 | 1,545 | Smaller |
| 1 | NBPF20 | 145,315,599 | 145,316,206 | 608 | 145,292,479 | 145,393,437 | 1,545 | Smaller |
| 1 | NBPF20 | 145,316,259 | 145,316,977 | 719 | 145,292,479 | 145,393,437 | 1,545 | Smaller |
| 1 | NBPF20 | 145,317,151 | 145,317,780 | 630 | 145,292,479 | 145,393,437 | 1,545 | Smaller |
| 1 | NBPF20 | 145,317,890 | 145,318,603 | 714 | 145,292,479 | 145,393,437 | 1,545 | Smaller |
| 1 | NBPF20 | 145,318,777 | 145,319,434 | 658 | 145,292,479 | 145,393,437 | 1,545 | Smaller |
| 1 | NBPF20 | 145,319,487 | 145,320,195 | 709 | 145,292,479 | 145,393,437 | 1,545 | Smaller |
| 1 | NBPF20 | 145,320,369 | 145,320,968 | 600 | 145,292,479 | 145,393,437 | 1,545 | Smaller |
| 1 | NBPF20 | 145,321,021 | 145,321,739 | 719 | 145,292,479 | 145,393,437 | 1,545 | Smaller |
| 1 | NBPF20 | 145,321,913 | 145,322,542 | 630 | 145,292,479 | 145,393,437 | 1,545 | Smaller |
| 1 | NBPF20 | 145,322,652 | 145,323,365 | 714 | 145,292,479 | 145,393,437 | 1,545 | Smaller |
| 1 | NBPF20 | 145,323,539 | 145,324,184 | 646 | 145,292,479 | 145,393,437 | 1,545 | Smaller |
| 1 | NBPF20 | 145,324,237 | 145,324,945 | 709 | 145,292,479 | 145,393,437 | 1,545 | Smaller |
| 1 | NBPF20 | 145,325,119 | 145,325,718 | 600 | 145,292,479 | 145,393,437 | 1,545 | Smaller |
| 1 | NBPF20 | 145,325,771 | 145,326,489 | 719 | 145,292,479 | 145,393,437 | 1,545 | Smaller |
| 1 | NBPF20 | 145,326,663 | 145,327,290 | 628 | 145,292,479 | 145,393,437 | 1,545 | Smaller |
| 1 | NBPF20 | 145,327,400 | 145,328,113 | 714 | 145,292,479 | 145,393,437 | 1,545 | Smaller |
| 1 | NBPF20 | 145,328,287 | 145,328,944 | 658 | 145,292,479 | 145,393,437 | 1,545 | Smaller |
| 1 | NBPF20 | 145,328,997 | 145,329,705 | 709 | 145,292,479 | 145,393,437 | 1,545 | Smaller |
| 1 | NBPF20 | 145,329,879 | 145,330,485 | 607 | 145,292,479 | 145,393,437 | 1,545 | Smaller |
| 1 | NBPF20 | 145,330,538 | 145,331,256 | 719 | 145,292,479 | 145,393,437 | 1,545 | Smaller |
| 1 | NBPF20 | 145,331,430 | 145,332,057 | 628 | 145,292,479 | 145,393,437 | 1,545 | Smaller |
| 1 | NBPF20 | 145,332,167 | 145,332,880 | 714 | 145,292,479 | 145,393,437 | 1,545 | Smaller |
| 1 | NBPF20 | 145,333,054 | 145,333,697 | 644 | 145,292,479 | 145,393,437 | 1,545 | Smaller |
| 1 | NBPF20 | 145,333,750 | 145,334,458 | 709 | 145,292,479 | 145,393,437 | 1,545 | Smaller |
| 1 | NBPF20 | 145,334,632 | 145,335,241 | 610 | 145,292,479 | 145,393,437 | 1,545 | Smaller |
| 1 | NBPF20 | 145,335,294 | 145,336,012 | 719 | 145,292,479 | 145,393,437 | 1,545 | Smaller |
| 1 | NBPF20 | 145,336,186 | 145,336,813 | 628 | 145,292,479 | 145,393,437 | 1,545 | Smaller |
| 1 | NBPF20 | 145,336,923 | 145,337,636 | 714 | 145,292,479 | 145,393,437 | 1,545 | Smaller |
| 1 | NBPF20 | 145,337,810 | 145,338,453 | 644 | 145,292,479 | 145,393,437 | 1,545 | Smaller |
| 1 | NBPF20 | 145,338,506 | 145,339,214 | 709 | 145,292,479 | 145,393,437 | 1,545 | Smaller |
| 1 | NBPF20 | 145,339,388 | 145,339,995 | 608 | 145,292,479 | 145,393,437 | 1,545 | Smaller |
| 1 | NBPF20 | 145,340,048 | 145,340,766 | 719 | 145,292,479 | 145,393,437 | 1,545 | Smaller |
| 1 | NBPF20 | 145,340,940 | 145,341,563 | 624 | 145,292,479 | 145,393,437 | 1,545 | Smaller |
| 1 | NBPF20 | 145,341,673 | 145,342,386 | 714 | 145,292,479 | 145,393,437 | 1,545 | Smaller |
| 1 | NBPF20 | 145,342,560 | 145,343,207 | 648 | 145,292,479 | 145,393,437 | 1,545 | Smaller |
| 1 | NBPF20 | 145,343,260 | 145,343,968 | 709 | 145,292,479 | 145,393,437 | 1,545 | Smaller |
| 1 | NBPF20 | 145,344,142 | 145,344,749 | 608 | 145,292,479 | 145,393,437 | 1,545 | Smaller |
| 1 | NBPF20 | 145,344,802 | 145,345,520 | 719 | 145,292,479 | 145,393,437 | 1,545 | Smaller |
| 1 | NBPF20 | 145,345,694 | 145,346,321 | 628 | 145,292,479 | 145,393,437 | 1,545 | Smaller |
| 1 | NBPF20 | 145,346,431 | 145,347,144 | 714 | 145,292,479 | 145,393,437 | 1,545 | Smaller |
| 1 | NBPF20 | 145,347,318 | 145,347,977 | 660 | 145,292,479 | 145,393,437 | 1,545 | Smaller |
| 1 | NBPF20 | 145,348,030 | 145,348,738 | 709 | 145,292,479 | 145,393,437 | 1,545 | Smaller |
| 1 | NBPF20 | 145,348,912 | 145,349,519 | 608 | 145,292,479 | 145,393,437 | 1,545 | Smaller |
| 1 | NBPF20 | 145,349,572 | 145,350,290 | 719 | 145,292,479 | 145,393,437 | 1,545 | Smaller |
| 1 | NBPF20 | 145,350,464 | 145,351,093 | 630 | 145,292,479 | 145,393,437 | 1,545 | Smaller |
| 1 | NBPF20 | 145,351,203 | 145,351,916 | 714 | 145,292,479 | 145,393,437 | 1,545 | Smaller |
| 1 | NBPF20 | 145,352,090 | 145,352,741 | 652 | 145,292,479 | 145,393,437 | 1,545 | Smaller |
| 1 | NBPF20 | 145,352,794 | 145,353,502 | 709 | 145,292,479 | 145,393,437 | 1,545 | Smaller |
| 1 | NBPF20 | 145,353,676 | 145,354,283 | 608 | 145,292,479 | 145,393,437 | 1,545 | Smaller |
| 1 | NBPF20 | 145,354,336 | 145,355,054 | 719 | 145,292,479 | 145,393,437 | 1,545 | Smaller |
| 1 | NBPF20 | 145,355,228 | 145,355,855 | 628 | 145,292,479 | 145,393,437 | 1,545 | Smaller |
| 1 | NBPF20 | 145,355,965 | 145,356,678 | 714 | 145,292,479 | 145,393,437 | 1,545 | Smaller |
| 1 | NBPF20 | 145,356,852 | 145,357,489 | 638 | 145,292,479 | 145,393,437 | 1,545 | Smaller |
| 1 | NBPF20 | 145,357,542 | 145,358,250 | 709 | 145,292,479 | 145,393,437 | 1,545 | Smaller |
| 1 | NBPF20 | 145,358,424 | 145,359,030 | 607 | 145,292,479 | 145,393,437 | 1,545 | Smaller |
| 1 | NBPF20 | 145,359,083 | 145,359,801 | 719 | 145,292,479 | 145,393,437 | 1,545 | Smaller |
| 1 | NBPF20 | 145,359,975 | 145,360,602 | 628 | 145,292,479 | 145,393,437 | 1,545 | Smaller |
| 1 | NBPF20 | 145,360,712 | 145,361,425 | 714 | 145,292,479 | 145,393,437 | 1,545 | Smaller |
| 1 | NBPF20 | 145,361,599 | 145,362,242 | 644 | 145,292,479 | 145,393,437 | 1,545 | Smaller |
| 1 | NBPF20 | 145,362,295 | 145,363,003 | 709 | 145,292,479 | 145,393,437 | 1,545 | Smaller |
| 1 | NBPF20 | 145,363,177 | 145,363,784 | 608 | 145,292,479 | 145,393,437 | 1,545 | Smaller |
| 1 | NBPF20 | 145,363,837 | 145,364,555 | 719 | 145,292,479 | 145,393,437 | 1,545 | Smaller |
| 1 | NBPF20 | 145,364,729 | 145,365,356 | 628 | 145,292,479 | 145,393,437 | 1,545 | Smaller |
| 1 | NBPF20 | 145,365,466 | 145,366,179 | 714 | 145,292,479 | 145,393,437 | 1,545 | Smaller |
| 1 | NBPF20 | 145,366,353 | 145,367,002 | 650 | 145,292,479 | 145,393,437 | 1,545 | Smaller |
| 1 | NBPF20 | 145,367,055 | 145,367,763 | 709 | 145,292,479 | 145,393,437 | 1,545 | Smaller |
| 1 | NBPF20 | 145,367,937 | 145,368,544 | 608 | 145,292,479 | 145,393,437 | 1,545 | Smaller |
| 1 | NBPF20 | 145,368,597 | 145,369,315 | 719 | 145,292,479 | 145,393,437 | 1,545 | Smaller |
| 1 | NBPF20 | 145,369,489 | 145,370,116 | 628 | 145,292,479 | 145,393,437 | 1,545 | Smaller |
| 1 | NBPF20 | 145,370,226 | 145,370,939 | 714 | 145,292,479 | 145,393,437 | 1,545 | Smaller |
| 1 | NBPF20 | 145,371,113 | 145,371,750 | 638 | 145,292,479 | 145,393,437 | 1,545 | Smaller |
| 1 | NBPF20 | 145,371,803 | 145,372,511 | 709 | 145,292,479 | 145,393,437 | 1,545 | Smaller |
| 1 | NBPF20 | 145,372,685 | 145,373,292 | 608 | 145,292,479 | 145,393,437 | 1,545 | Smaller |
| 1 | NBPF20 | 145,373,345 | 145,374,063 | 719 | 145,292,479 | 145,393,437 | 1,545 | Smaller |
| 1 | NBPF20 | 145,374,237 | 145,374,864 | 628 | 145,292,479 | 145,393,437 | 1,545 | Smaller |
| 1 | NBPF20 | 145,374,974 | 145,375,687 | 714 | 145,292,479 | 145,393,437 | 1,545 | Smaller |
| 1 | NBPF20 | 145,375,861 | 145,376,504 | 644 | 145,292,479 | 145,393,437 | 1,545 | Smaller |
| 1 | NBPF20 | 145,376,557 | 145,377,265 | 709 | 145,292,479 | 145,393,437 | 1,545 | Smaller |
| 1 | NBPF20 | 145,377,439 | 145,378,042 | 604 | 145,292,479 | 145,393,437 | 1,545 | Smaller |
| 1 | NBPF20 | 145,378,095 | 145,378,813 | 719 | 145,292,479 | 145,393,437 | 1,545 | Smaller |
| 1 | NBPF20 | 145,378,987 | 145,379,616 | 630 | 145,292,479 | 145,393,437 | 1,545 | Smaller |
| 1 | NBPF20 | 145,379,726 | 145,380,439 | 714 | 145,292,479 | 145,393,437 | 1,545 | Smaller |
| 1 | NBPF20 | 145,380,613 | 145,381,256 | 644 | 145,292,479 | 145,393,437 | 1,545 | Smaller |
| 1 | NBPF20 | 145,381,309 | 145,382,017 | 709 | 145,292,479 | 145,393,437 | 1,545 | Smaller |
| 1 | NBPF20 | 145,382,191 | 145,382,798 | 608 | 145,292,479 | 145,393,437 | 1,545 | Smaller |
| 1 | NBPF20 | 145,382,851 | 145,383,569 | 719 | 145,292,479 | 145,393,437 | 1,545 | Smaller |
| 1 | NBPF20 | 145,383,743 | 145,384,370 | 628 | 145,292,479 | 145,393,437 | 1,545 | Smaller |
| 1 | NBPF20 | 145,384,480 | 145,385,193 | 714 | 145,292,479 | 145,393,437 | 1,545 | Smaller |
| 1 | NBPF20 | 145,385,367 | 145,386,012 | 646 | 145,292,479 | 145,393,437 | 1,545 | Smaller |
| 1 | NBPF20 | 145,386,065 | 145,386,773 | 709 | 145,292,479 | 145,393,437 | 1,545 | Smaller |
| 1 | NBPF20 | 145,386,947 | 145,387,546 | 600 | 145,292,479 | 145,393,437 | 1,545 | Smaller |
| 1 | NBPF20 | 145,387,599 | 145,388,317 | 719 | 145,292,479 | 145,393,437 | 1,545 | Smaller |
| 1 | NBPF20 | 145,388,491 | 145,389,120 | 630 | 145,292,479 | 145,393,437 | 1,545 | Smaller |
| 1 | NBPF20 | 145,389,230 | 145,389,943 | 714 | 145,292,479 | 145,393,437 | 1,545 | Smaller |
| 1 | NBPF20 | 145,390,117 | 145,390,768 | 652 | 145,292,479 | 145,393,437 | 1,545 | Smaller |
| 1 | NBPF20 | 145,390,821 | 145,391,529 | 709 | 145,292,479 | 145,393,437 | 1,545 | Smaller |
| 1 | NBPF20 | 145,391,703 | 145,392,302 | 600 | 145,292,479 | 145,393,437 | 1,545 | Smaller |
| 1 | NBPF20 | 145,392,355 | 145,393,073 | 719 | 145,292,479 | 145,393,437 | 1,545 | Smaller |
| 1 | NBPF25P | 145,574,933 | 145,575,646 | 714 | 145,574,922 | 145,579,485 | 1,555 | Smaller |
| 1 | NBPF25P | 145,575,820 | 145,576,459 | 640 | 145,574,922 | 145,579,485 | 1,555 | Smaller |
| 1 | NBPF25P | 145,576,512 | 145,577,220 | 709 | 145,574,922 | 145,579,485 | 1,555 | Smaller |
| 1 | NBPF25P | 145,577,394 | 145,578,011 | 618 | 145,574,922 | 145,579,485 | 1,555 | Smaller |
| 1 | NBPF25P | 145,578,064 | 145,578,782 | 719 | 145,574,922 | 145,579,485 | 1,555 | Smaller |
| 1 | NBPF10 | 146,067,289 | 146,068,002 | 714 | 146,067,278 | 146,126,918 | 1,540 | Smaller |
| 1 | NBPF10 | 146,068,176 | 146,068,771 | 596 | 146,067,278 | 146,126,918 | 1,540 | Smaller |
| 1 | NBPF10 | 146,068,824 | 146,069,542 | 719 | 146,067,278 | 146,126,918 | 1,540 | Smaller |
| 1 | NBPF10 | 146,069,716 | 146,070,323 | 608 | 146,067,278 | 146,126,918 | 1,540 | Smaller |
| 1 | NBPF10 | 146,070,433 | 146,071,146 | 714 | 146,067,278 | 146,126,918 | 1,540 | Smaller |
| 1 | NBPF10 | 146,071,320 | 146,071,967 | 648 | 146,067,278 | 146,126,918 | 1,540 | Smaller |
| 1 | NBPF10 | 146,072,020 | 146,072,728 | 709 | 146,067,278 | 146,126,918 | 1,540 | Smaller |
| 1 | NBPF10 | 146,072,902 | 146,073,499 | 598 | 146,067,278 | 146,126,918 | 1,540 | Smaller |
| 1 | NBPF10 | 146,073,552 | 146,074,270 | 719 | 146,067,278 | 146,126,918 | 1,540 | Smaller |
| 1 | NBPF10 | 146,074,444 | 146,075,045 | 602 | 146,067,278 | 146,126,918 | 1,540 | Smaller |
| 1 | NBPF10 | 146,074,505 | 146,074,571 | 67 | 146,067,278 | 146,126,918 | 1,540 | Smaller |
| 1 | NBPF10 | 146,074,718 | 146,075,045 | 328 | 146,067,278 | 146,126,918 | 1,540 | Smaller |
| 1 | NBPF10 | 146,075,155 | 146,075,868 | 714 | 146,067,278 | 146,126,918 | 1,540 | Smaller |
| 1 | NBPF10 | 146,076,042 | 146,076,705 | 664 | 146,067,278 | 146,126,918 | 1,540 | Smaller |
| 1 | NBPF10 | 146,076,758 | 146,077,466 | 709 | 146,067,278 | 146,126,918 | 1,540 | Smaller |
| 1 | NBPF10 | 146,077,640 | 146,078,235 | 596 | 146,067,278 | 146,126,918 | 1,540 | Smaller |
| 1 | NBPF10 | 146,078,288 | 146,079,006 | 719 | 146,067,278 | 146,126,918 | 1,540 | Smaller |
| 1 | NBPF10 | 146,079,180 | 146,079,781 | 602 | 146,067,278 | 146,126,918 | 1,540 | Smaller |
| 1 | NBPF10 | 146,079,891 | 146,080,604 | 714 | 146,067,278 | 146,126,918 | 1,540 | Smaller |
| 1 | NBPF10 | 146,080,778 | 146,081,435 | 658 | 146,067,278 | 146,126,918 | 1,540 | Smaller |
| 1 | NBPF10 | 146,081,488 | 146,082,196 | 709 | 146,067,278 | 146,126,918 | 1,540 | Smaller |
| 1 | NBPF10 | 146,082,370 | 146,082,965 | 596 | 146,067,278 | 146,126,918 | 1,540 | Smaller |
| 1 | NBPF10 | 146,083,018 | 146,083,736 | 719 | 146,067,278 | 146,126,918 | 1,540 | Smaller |
| 1 | NBPF10 | 146,083,910 | 146,084,517 | 608 | 146,067,278 | 146,126,918 | 1,540 | Smaller |
| 1 | NBPF10 | 146,084,627 | 146,085,340 | 714 | 146,067,278 | 146,126,918 | 1,540 | Smaller |
| 1 | NBPF10 | 146,085,514 | 146,086,163 | 650 | 146,067,278 | 146,126,918 | 1,540 | Smaller |
| 1 | NBPF10 | 146,086,216 | 146,086,923 | 708 | 146,067,278 | 146,126,918 | 1,540 | Smaller |
| 1 | NBPF10 | 146,087,097 | 146,087,692 | 596 | 146,067,278 | 146,126,918 | 1,540 | Smaller |
| 1 | NBPF10 | 146,087,745 | 146,088,463 | 719 | 146,067,278 | 146,126,918 | 1,540 | Smaller |
| 1 | NBPF10 | 146,088,637 | 146,089,238 | 602 | 146,067,278 | 146,126,918 | 1,540 | Smaller |
| 1 | NBPF10 | 146,089,348 | 146,090,061 | 714 | 146,067,278 | 146,126,918 | 1,540 | Smaller |
| 1 | NBPF10 | 146,090,235 | 146,090,880 | 646 | 146,067,278 | 146,126,918 | 1,540 | Smaller |
| 1 | NBPF10 | 146,090,933 | 146,091,641 | 709 | 146,067,278 | 146,126,918 | 1,540 | Smaller |
| 1 | NBPF10 | 146,091,815 | 146,092,410 | 596 | 146,067,278 | 146,126,918 | 1,540 | Smaller |
| 1 | NBPF10 | 146,092,463 | 146,093,181 | 719 | 146,067,278 | 146,126,918 | 1,540 | Smaller |
| 1 | NBPF10 | 146,093,355 | 146,093,956 | 602 | 146,067,278 | 146,126,918 | 1,540 | Smaller |
| 1 | NBPF10 | 146,094,066 | 146,094,779 | 714 | 146,067,278 | 146,126,918 | 1,540 | Smaller |
| 1 | NBPF10 | 146,094,953 | 146,095,602 | 650 | 146,067,278 | 146,126,918 | 1,540 | Smaller |
| 1 | NBPF10 | 146,095,655 | 146,096,363 | 709 | 146,067,278 | 146,126,918 | 1,540 | Smaller |
| 1 | NBPF10 | 146,096,537 | 146,097,132 | 596 | 146,067,278 | 146,126,918 | 1,540 | Smaller |
| 1 | NBPF10 | 146,097,185 | 146,097,903 | 719 | 146,067,278 | 146,126,918 | 1,540 | Smaller |
| 1 | NBPF10 | 146,098,077 | 146,098,678 | 602 | 146,067,278 | 146,126,918 | 1,540 | Smaller |
| 1 | NBPF10 | 146,098,788 | 146,099,501 | 714 | 146,067,278 | 146,126,918 | 1,540 | Smaller |
| 1 | NBPF10 | 146,099,675 | 146,100,324 | 650 | 146,067,278 | 146,126,918 | 1,540 | Smaller |
| 1 | NBPF10 | 146,100,377 | 146,101,085 | 709 | 146,067,278 | 146,126,918 | 1,540 | Smaller |
| 1 | NBPF10 | 146,101,259 | 146,101,854 | 596 | 146,067,278 | 146,126,918 | 1,540 | Smaller |
| 1 | NBPF10 | 146,101,907 | 146,102,625 | 719 | 146,067,278 | 146,126,918 | 1,540 | Smaller |
| 1 | NBPF10 | 146,102,799 | 146,103,400 | 602 | 146,067,278 | 146,126,918 | 1,540 | Smaller |
| 1 | NBPF10 | 146,103,510 | 146,104,223 | 714 | 146,067,278 | 146,126,918 | 1,540 | Smaller |
| 1 | NBPF10 | 146,104,397 | 146,105,042 | 646 | 146,067,278 | 146,126,918 | 1,540 | Smaller |
| 1 | NBPF10 | 146,105,095 | 146,105,803 | 709 | 146,067,278 | 146,126,918 | 1,540 | Smaller |
| 1 | NBPF10 | 146,105,977 | 146,106,572 | 596 | 146,067,278 | 146,126,918 | 1,540 | Smaller |
| 1 | NBPF10 | 146,106,625 | 146,107,343 | 719 | 146,067,278 | 146,126,918 | 1,540 | Smaller |
| 1 | NBPF10 | 146,107,517 | 146,108,118 | 602 | 146,067,278 | 146,126,918 | 1,540 | Smaller |
| 1 | NBPF10 | 146,108,228 | 146,108,941 | 714 | 146,067,278 | 146,126,918 | 1,540 | Smaller |
| 1 | NBPF10 | 146,109,115 | 146,109,750 | 636 | 146,067,278 | 146,126,918 | 1,540 | Smaller |
| 1 | NBPF10 | 146,109,803 | 146,110,511 | 709 | 146,067,278 | 146,126,918 | 1,540 | Smaller |
| 1 | NBPF10 | 146,110,685 | 146,111,280 | 596 | 146,067,278 | 146,126,918 | 1,540 | Smaller |
| 1 | NBPF10 | 146,111,333 | 146,112,053 | 721 | 146,067,278 | 146,126,918 | 1,540 | Smaller |
| 1 | NBPF10 | 146,112,227 | 146,112,828 | 602 | 146,067,278 | 146,126,918 | 1,540 | Smaller |
| 1 | NBPF10 | 146,112,938 | 146,113,651 | 714 | 146,067,278 | 146,126,918 | 1,540 | Smaller |
| 1 | NBPF10 | 146,113,825 | 146,114,468 | 644 | 146,067,278 | 146,126,918 | 1,540 | Smaller |
| 1 | NBPF10 | 146,114,521 | 146,115,229 | 709 | 146,067,278 | 146,126,918 | 1,540 | Smaller |
| 1 | NBPF10 | 146,114,521 | 146,121,497 | 6,977 | 146,067,278 | 146,126,918 | 1,540 | Larger |
| 1 | NBPF10 | 146,115,403 | 146,115,998 | 596 | 146,067,278 | 146,126,918 | 1,540 | Smaller |
| 1 | NBPF10 | 146,116,051 | 146,116,771 | 721 | 146,067,278 | 146,126,918 | 1,540 | Smaller |
| 1 | NBPF10 | 146,116,945 | 146,117,546 | 602 | 146,067,278 | 146,126,918 | 1,540 | Smaller |
| 1 | NBPF10 | 146,117,656 | 146,118,369 | 714 | 146,067,278 | 146,126,918 | 1,540 | Smaller |
| 1 | NBPF10 | 146,118,543 | 146,119,194 | 652 | 146,067,278 | 146,126,918 | 1,540 | Smaller |
| 1 | NBPF10 | 146,119,247 | 146,119,955 | 709 | 146,067,278 | 146,126,918 | 1,540 | Smaller |
| 1 | NBPF10 | 146,120,129 | 146,120,724 | 596 | 146,067,278 | 146,126,918 | 1,540 | Smaller |
| 1 | NBPF10 | 146,120,777 | 146,121,497 | 721 | 146,067,278 | 146,126,918 | 1,540 | Smaller |
| 1 | NBPF10 | 146,121,671 | 146,122,282 | 612 | 146,067,278 | 146,126,918 | 1,540 | Smaller |
| 1 | NBPF10 | 146,122,392 | 146,123,105 | 714 | 146,067,278 | 146,126,918 | 1,540 | Smaller |
| 1 | NBPF10 | 146,123,279 | 146,123,926 | 648 | 146,067,278 | 146,126,918 | 1,540 | Smaller |
| 1 | NBPF10 | 146,123,979 | 146,124,687 | 709 | 146,067,278 | 146,126,918 | 1,540 | Smaller |
| 1 | NBPF10 | 146,124,861 | 146,125,464 | 604 | 146,067,278 | 146,126,918 | 1,540 | Smaller |
| 1 | NBPF10 | 146,125,517 | 146,126,235 | 719 | 146,067,278 | 146,126,918 | 1,540 | Smaller |
| 1 | NBPF11 | 148,106,233 | 148,106,941 | 709 | 148,105,777 | 148,109,173 | 1,532 | Smaller |
| 1 | NBPF11 | 148,107,115 | 148,107,710 | 596 | 148,105,777 | 148,109,173 | 1,532 | Smaller |
| 1 | NBPF11 | 148,107,763 | 148,108,481 | 719 | 148,105,777 | 148,109,173 | 1,532 | Smaller |
| 1 | NBPF14 | 148,533,970 | 148,534,683 | 714 | 148,533,955 | 148,577,873 | 1,561 | Smaller |
| 1 | NBPF14 | 148,534,857 | 148,535,452 | 596 | 148,533,955 | 148,577,873 | 1,561 | Smaller |
| 1 | NBPF14 | 148,535,505 | 148,536,223 | 719 | 148,533,955 | 148,577,873 | 1,561 | Smaller |
| 1 | NBPF14 | 148,535,505 | 148,551,975 | 16,471 | 148,533,955 | 148,577,873 | 1,561 | Larger |
| 1 | NBPF14 | 148,536,397 | 148,537,010 | 614 | 148,533,955 | 148,577,873 | 1,561 | Smaller |
| 1 | NBPF14 | 148,537,120 | 148,537,833 | 714 | 148,533,955 | 148,577,873 | 1,561 | Smaller |
| 1 | NBPF14 | 148,538,007 | 148,538,648 | 642 | 148,533,955 | 148,577,873 | 1,561 | Smaller |
| 1 | NBPF14 | 148,538,701 | 148,539,409 | 709 | 148,533,955 | 148,577,873 | 1,561 | Smaller |
| 1 | NBPF14 | 148,539,583 | 148,540,178 | 596 | 148,533,955 | 148,577,873 | 1,561 | Smaller |
| 1 | NBPF14 | 148,540,231 | 148,540,949 | 719 | 148,533,955 | 148,577,873 | 1,561 | Smaller |
| 1 | NBPF14 | 148,541,123 | 148,541,730 | 608 | 148,533,955 | 148,577,873 | 1,561 | Smaller |
| 1 | NBPF14 | 148,541,840 | 148,542,553 | 714 | 148,533,955 | 148,577,873 | 1,561 | Smaller |
| 1 | NBPF14 | 148,542,727 | 148,543,362 | 636 | 148,533,955 | 148,577,873 | 1,561 | Smaller |
| 1 | NBPF14 | 148,543,415 | 148,544,123 | 709 | 148,533,955 | 148,577,873 | 1,561 | Smaller |
| 1 | NBPF14 | 148,544,297 | 148,544,892 | 596 | 148,533,955 | 148,577,873 | 1,561 | Smaller |
| 1 | NBPF14 | 148,544,945 | 148,545,663 | 719 | 148,533,955 | 148,577,873 | 1,561 | Smaller |
| 1 | NBPF14 | 148,545,837 | 148,546,444 | 608 | 148,533,955 | 148,577,873 | 1,561 | Smaller |
| 1 | NBPF14 | 148,546,554 | 148,547,267 | 714 | 148,533,955 | 148,577,873 | 1,561 | Smaller |
| 1 | NBPF14 | 148,547,441 | 148,548,070 | 630 | 148,533,955 | 148,577,873 | 1,561 | Smaller |
| 1 | NBPF14 | 148,548,123 | 148,548,831 | 709 | 148,533,955 | 148,577,873 | 1,561 | Smaller |
| 1 | NBPF14 | 148,549,005 | 148,549,600 | 596 | 148,533,955 | 148,577,873 | 1,561 | Smaller |
| 1 | NBPF14 | 148,549,653 | 148,550,371 | 719 | 148,533,955 | 148,577,873 | 1,561 | Smaller |
| 1 | NBPF14 | 148,550,545 | 148,551,152 | 608 | 148,533,955 | 148,577,873 | 1,561 | Smaller |
| 1 | NBPF14 | 148,551,262 | 148,551,975 | 714 | 148,533,955 | 148,577,873 | 1,561 | Smaller |
| 1 | NBPF14 | 148,552,149 | 148,552,778 | 630 | 148,533,955 | 148,577,873 | 1,561 | Smaller |
| 1 | NBPF14 | 148,552,831 | 148,553,539 | 709 | 148,533,955 | 148,577,873 | 1,561 | Smaller |
| 1 | NBPF14 | 148,552,831 | 148,562,996 | 10,166 | 148,533,955 | 148,577,873 | 1,561 | Larger |
| 1 | NBPF14 | 148,552,831 | 148,575,638 | 22,808 | 148,533,955 | 148,577,873 | 1,561 | Larger |
| 1 | NBPF14 | 148,553,713 | 148,554,308 | 596 | 148,533,955 | 148,577,873 | 1,561 | Smaller |
| 1 | NBPF14 | 148,554,361 | 148,555,079 | 719 | 148,533,955 | 148,577,873 | 1,561 | Smaller |
| 1 | NBPF14 | 148,555,253 | 148,555,866 | 614 | 148,533,955 | 148,577,873 | 1,561 | Smaller |
| 1 | NBPF14 | 148,555,976 | 148,556,689 | 714 | 148,533,955 | 148,577,873 | 1,561 | Smaller |
| 1 | NBPF14 | 148,556,863 | 148,557,490 | 628 | 148,533,955 | 148,577,873 | 1,561 | Smaller |
| 1 | NBPF14 | 148,557,543 | 148,558,251 | 709 | 148,533,955 | 148,577,873 | 1,561 | Smaller |
| 1 | NBPF14 | 148,558,425 | 148,559,020 | 596 | 148,533,955 | 148,577,873 | 1,561 | Smaller |
| 1 | NBPF14 | 148,559,073 | 148,559,792 | 720 | 148,533,955 | 148,577,873 | 1,561 | Smaller |
| 1 | NBPF14 | 148,559,966 | 148,560,583 | 618 | 148,533,955 | 148,577,873 | 1,561 | Smaller |
| 1 | NBPF14 | 148,560,693 | 148,561,406 | 714 | 148,533,955 | 148,577,873 | 1,561 | Smaller |
| 1 | NBPF14 | 148,561,580 | 148,562,235 | 656 | 148,533,955 | 148,577,873 | 1,561 | Smaller |
| 1 | NBPF14 | 148,562,288 | 148,562,996 | 709 | 148,533,955 | 148,577,873 | 1,561 | Smaller |
| 1 | NBPF14 | 148,563,170 | 148,563,765 | 596 | 148,533,955 | 148,577,873 | 1,561 | Smaller |
| 1 | NBPF14 | 148,563,818 | 148,564,538 | 721 | 148,533,955 | 148,577,873 | 1,561 | Smaller |
| 1 | NBPF14 | 148,564,712 | 148,565,319 | 608 | 148,533,955 | 148,577,873 | 1,561 | Smaller |
| 1 | NBPF14 | 148,564,712 | 148,568,481 | 3,770 | 148,533,955 | 148,577,873 | 1,561 | Larger |
| 1 | NBPF14 | 148,565,429 | 148,566,142 | 714 | 148,533,955 | 148,577,873 | 1,561 | Smaller |
| 1 | NBPF14 | 148,566,316 | 148,566,951 | 636 | 148,533,955 | 148,577,873 | 1,561 | Smaller |
| 1 | NBPF14 | 148,567,004 | 148,567,712 | 709 | 148,533,955 | 148,577,873 | 1,561 | Smaller |
| 1 | NBPF14 | 148,567,886 | 148,568,481 | 596 | 148,533,955 | 148,577,873 | 1,561 | Smaller |
| 1 | NBPF14 | 148,568,534 | 148,569,254 | 721 | 148,533,955 | 148,577,873 | 1,561 | Smaller |
| 1 | NBPF14 | 148,569,428 | 148,570,041 | 614 | 148,533,955 | 148,577,873 | 1,561 | Smaller |
| 1 | NBPF14 | 148,569,428 | 148,573,229 | 3,802 | 148,533,955 | 148,577,873 | 1,561 | Larger |
| 1 | NBPF14 | 148,570,151 | 148,570,864 | 714 | 148,533,955 | 148,577,873 | 1,561 | Smaller |
| 1 | NBPF14 | 148,571,038 | 148,571,681 | 644 | 148,533,955 | 148,577,873 | 1,561 | Smaller |
| 1 | NBPF14 | 148,571,038 | 148,574,877 | 3,840 | 148,533,955 | 148,577,873 | 1,561 | Larger |
| 1 | NBPF14 | 148,571,734 | 148,572,442 | 709 | 148,533,955 | 148,577,873 | 1,561 | Smaller |
| 1 | NBPF14 | 148,572,616 | 148,573,229 | 614 | 148,533,955 | 148,577,873 | 1,561 | Smaller |
| 1 | NBPF14 | 148,573,339 | 148,574,052 | 714 | 148,533,955 | 148,577,873 | 1,561 | Smaller |
| 1 | NBPF14 | 148,574,226 | 148,574,877 | 652 | 148,533,955 | 148,577,873 | 1,561 | Smaller |
| 1 | NBPF14 | 148,574,930 | 148,575,638 | 709 | 148,533,955 | 148,577,873 | 1,561 | Smaller |
| 1 | NBPF14 | 148,575,812 | 148,576,409 | 598 | 148,533,955 | 148,577,873 | 1,561 | Smaller |
| 1 | NBPF14 | 148,576,462 | 148,577,182 | 721 | 148,533,955 | 148,577,873 | 1,561 | Smaller |
| 1 | NBPF9 | 149,056,621 | 149,057,334 | 714 | 149,056,610 | 149,064,320 | 1,536 | Smaller |
| 1 | NBPF9 | 149,056,621 | 149,060,522 | 3,902 | 149,056,610 | 149,064,320 | 1,536 | Larger |
| 1 | NBPF9 | 149,057,508 | 149,058,163 | 656 | 149,056,610 | 149,064,320 | 1,536 | Smaller |
| 1 | NBPF9 | 149,058,216 | 149,058,924 | 709 | 149,056,610 | 149,064,320 | 1,536 | Smaller |
| 1 | NBPF9 | 149,058,216 | 149,062,092 | 3,877 | 149,056,610 | 149,064,320 | 1,536 | Larger |
| 1 | NBPF9 | 149,059,098 | 149,059,699 | 602 | 149,056,610 | 149,064,320 | 1,536 | Smaller |
| 1 | NBPF9 | 149,059,098 | 149,062,861 | 3,764 | 149,056,610 | 149,064,320 | 1,536 | Larger |
| 1 | NBPF9 | 149,059,809 | 149,060,522 | 714 | 149,056,610 | 149,064,320 | 1,536 | Smaller |
| 1 | NBPF9 | 149,060,696 | 149,061,331 | 636 | 149,056,610 | 149,064,320 | 1,536 | Smaller |
| 1 | NBPF9 | 149,061,384 | 149,062,092 | 709 | 149,056,610 | 149,064,320 | 1,536 | Smaller |
| 1 | NBPF9 | 149,062,266 | 149,062,861 | 596 | 149,056,610 | 149,064,320 | 1,536 | Smaller |
| 1 | NBPF9 | 149,062,914 | 149,063,632 | 719 | 149,056,610 | 149,064,320 | 1,536 | Smaller |
| 1 | ADAR | 154,601,889 | 154,602,035 | 147 | 154,601,766 | 154,602,040 | 147 | Equal |
| 1 | ENSG00000224691 | 186,176,909 | 186,177,044 | 136 | 186,176,895 | 186,177,204 | 136 | Equal |
| 1 | ENSG00000285945 | 2,768,092 | 2,777,298 | 9,207 | 2,746,298 | 2,777,443 | 40 | Larger |
| 1 | ENSG00000283259 | 2,773,865 | 2,773,946 | 82 | 2,746,298 | 2,777,443 | 40 | Larger |
| 1 | ENSG00000283259 | 2,774,067 | 2,774,229 | 163 | 2,746,298 | 2,777,443 | 40 | Larger |
| 1 | ENSG00000283259 | 2,774,710 | 2,774,789 | 80 | 2,746,298 | 2,777,443 | 40 | Larger |
| 1 | ENSG00000283259 | 2,774,911 | 2,774,992 | 82 | 2,746,298 | 2,777,443 | 40 | Larger |
| 1 | ENSG00000283259 | 2,775,073 | 2,775,154 | 82 | 2,746,298 | 2,777,443 | 40 | Larger |
| 1 | ENSG00000283259 | 2,775,275 | 2,775,356 | 82 | 2,746,298 | 2,777,443 | 40 | Larger |
| 1 | ENSG00000283259 | 2,775,438 | 2,775,518 | 81 | 2,746,298 | 2,777,443 | 40 | Larger |
| 1 | ENSG00000283259 | 2,775,635 | 2,776,048 | 414 | 2,746,298 | 2,777,443 | 40 | Larger |
| 1 | ENSG00000283259 | 2,776,130 | 2,776,369 | 240 | 2,746,298 | 2,777,443 | 40 | Larger |
| 1 | ENSG00000283259 | 2,776,130 | 2,776,369 | 240 | 2,776,006 | 2,777,443 | 81 | Larger |
| 1 | NBPF3 | 21,480,224 | 21,480,929 | 706 | 21,479,861 | 21,483,215 | 1,541 | Smaller |
| 1 | NBPF3 | 21,480,982 | 21,481,596 | 615 | 21,479,861 | 21,483,215 | 1,541 | Smaller |
| 1 | NBPF3 | 21,481,770 | 21,482,483 | 714 | 21,479,861 | 21,483,215 | 1,541 | Smaller |
| 1 | NBPF3 | 21,482,536 | 21,483,142 | 607 | 21,479,861 | 21,483,215 | 1,541 | Smaller |
| 1 | OBSCN | 228,243,459 | 228,244,295 | 837 | 228,243,180 | 228,246,793 | 1,110 | Smaller |
| 1 | OBSCN | 228,244,572 | 228,245,412 | 841 | 228,243,180 | 228,246,793 | 1,110 | Smaller |
| 1 | OBSCN | 228,245,689 | 228,246,527 | 839 | 228,243,180 | 228,246,793 | 1,110 | Smaller |
| 1 | ENSG00000238224 | 245,614,884 | 245,614,985 | 102 | 245,614,858 | 245,615,030 | 34 | Larger |
| 1 | TP73-AS1 | 3,739,228 | 3,739,623 | 396 | 3,739,214 | 3,740,189 | 36 | Larger |
| 1 | TP73-AS1 | 3,739,336 | 3,739,835 | 500 | 3,739,214 | 3,740,189 | 36 | Larger |
| 1 | TP73-AS1 | 3,739,408 | 3,739,695 | 288 | 3,739,214 | 3,740,189 | 36 | Larger |
| 1 | TP73-AS1 | 3,739,552 | 3,740,123 | 572 | 3,739,214 | 3,740,189 | 36 | Larger |
| 1 | ENSG00000281133 | 45,580,928 | 45,580,932 | 5 | 45,580,696 | 45,581,903 | 310 | Smaller |
| 1 | ENSG00000281133 | 45,580,966 | 45,580,969 | 4 | 45,580,696 | 45,581,903 | 310 | Smaller |
| 1 | ENSG00000280836 | 45,581,279 | 45,581,282 | 4 | 45,580,696 | 45,581,903 | 310 | Smaller |
| 1 | ENSG00000281825 | 45,605,693 | 45,605,697 | 5 | 45,605,469 | 45,606,060 | 312 | Smaller |
| 1 | ENSG00000284744 | 6,769,777 | 6,769,910 | 134 | 6,769,688 | 6,770,002 | 133 | Larger |
| 1 | PER3 | 7,829,952 | 7,830,005 | 54 | 7,829,888 | 7,830,126 | 54 | Equal |
| 1 | ENSG00000239945 | 90,051 | 90,286 | 236 | 90,048 | 90,430 | 59 | Larger |
| 2 | DPP10-AS2 | 114,834,039 | 114,835,173 | 1,135 | 114,834,033 | 114,835,457 | 29 | Larger |
| 2 | NEB | 151,496,369 | 151,496,940 | 572 | 151,496,110 | 151,498,125 | 673 | Smaller |
| 2 | NEB | 151,497,034 | 151,497,625 | 592 | 151,496,110 | 151,498,125 | 673 | Smaller |
| 2 | TTN | 178,652,348 | 178,652,457 | 110 | 178,652,272 | 178,653,523 | 194 | Smaller |
| 2 | TTN | 178,652,542 | 178,652,652 | 111 | 178,652,272 | 178,653,523 | 194 | Smaller |
| 2 | TTN | 178,652,737 | 178,652,847 | 111 | 178,652,272 | 178,653,523 | 194 | Smaller |
| 2 | TTN | 178,652,932 | 178,653,040 | 109 | 178,652,272 | 178,653,523 | 194 | Smaller |
| 2 | TTN | 178,653,125 | 178,653,237 | 113 | 178,652,272 | 178,653,523 | 194 | Smaller |
| 2 | TTN | 178,653,322 | 178,653,426 | 105 | 178,652,272 | 178,653,523 | 194 | Smaller |
| 2 | TTN | 178,653,508 | 178,653,620 | 113 | 178,653,496 | 178,653,988 | 193 | Smaller |
| 2 | TTN | 178,653,508 | 178,653,620 | 113 | 178,653,498 | 178,654,120 | 195 | Smaller |
| 2 | TTN | 178,653,702 | 178,653,816 | 115 | 178,653,496 | 178,653,988 | 193 | Smaller |
| 2 | TTN | 178,653,702 | 178,653,816 | 115 | 178,653,498 | 178,654,120 | 195 | Smaller |
| 2 | TTN | 178,653,898 | 178,654,011 | 114 | 178,653,498 | 178,654,120 | 195 | Smaller |
| 2 | TTN | 178,657,768 | 178,657,880 | 113 | 178,657,756 | 178,658,248 | 193 | Smaller |
| 2 | TTN | 178,657,768 | 178,657,880 | 113 | 178,657,758 | 178,658,380 | 195 | Smaller |
| 2 | TTN | 178,657,962 | 178,658,076 | 115 | 178,657,756 | 178,658,248 | 193 | Smaller |
| 2 | TTN | 178,657,962 | 178,658,076 | 115 | 178,657,758 | 178,658,380 | 195 | Smaller |
| 2 | TTN | 178,658,158 | 178,658,271 | 114 | 178,657,758 | 178,658,380 | 195 | Smaller |
| 2 | TTN | 178,662,029 | 178,662,141 | 113 | 178,662,017 | 178,662,509 | 193 | Smaller |
| 2 | TTN | 178,662,029 | 178,662,141 | 113 | 178,662,019 | 178,662,641 | 195 | Smaller |
| 2 | TTN | 178,662,223 | 178,662,337 | 115 | 178,662,017 | 178,662,509 | 193 | Smaller |
| 2 | TTN | 178,662,223 | 178,662,337 | 115 | 178,662,019 | 178,662,641 | 195 | Smaller |
| 2 | TTN | 178,662,419 | 178,662,532 | 114 | 178,662,019 | 178,662,641 | 195 | Smaller |
| 2 | TTN | 178,663,517 | 178,663,626 | 110 | 178,663,441 | 178,664,114 | 192 | Smaller |
| 2 | TTN | 178,663,711 | 178,663,818 | 108 | 178,663,441 | 178,664,114 | 192 | Smaller |
| 2 | TTN | 178,663,903 | 178,664,014 | 112 | 178,663,441 | 178,664,114 | 192 | Smaller |
| 2 | ENSG00000286588 | 241,326,511 | 241,326,659 | 149 | 241,326,401 | 241,326,699 | 149 | Equal |
| 2 | ENSG00000277997 | 307,745 | 308,938 | 1,194 | 306,343 | 308,994 | 35 | Larger |
| 2 | ANKRD36BP2 | 88,782,763 | 88,782,852 | 90 | 88,782,531 | 88,788,312 | 1,828 | Smaller |
| 2 | ANKRD36BP2 | 88,782,926 | 88,784,597 | 1,672 | 88,782,531 | 88,788,312 | 1,828 | Smaller |
| 2 | ANKRD36BP2 | 88,784,627 | 88,784,720 | 94 | 88,782,531 | 88,788,312 | 1,828 | Smaller |
| 2 | ANKRD36BP2 | 88,784,794 | 88,786,469 | 1,676 | 88,782,531 | 88,788,312 | 1,828 | Smaller |
| 2 | ANKRD36BP2 | 88,784,927 | 88,786,469 | 1,543 | 88,782,531 | 88,788,312 | 1,828 | Smaller |
| 2 | ANKRD36BP2 | 88,786,499 | 88,786,590 | 92 | 88,782,531 | 88,788,312 | 1,828 | Smaller |
| 2 | ANKRD36C | 95,882,375 | 95,882,468 | 94 | 95,880,875 | 95,930,413 | 1,868 | Smaller |
| 2 | ANKRD36C | 95,882,498 | 95,884,172 | 1,675 | 95,880,875 | 95,930,413 | 1,868 | Smaller |
| 2 | ANKRD36C | 95,884,246 | 95,884,339 | 94 | 95,880,875 | 95,930,413 | 1,868 | Smaller |
| 2 | ANKRD36C | 95,884,369 | 95,886,047 | 1,679 | 95,880,875 | 95,930,413 | 1,868 | Smaller |
| 2 | ANKRD36C | 95,886,121 | 95,886,215 | 95 | 95,880,875 | 95,930,413 | 1,868 | Smaller |
| 2 | ANKRD36C | 95,886,245 | 95,887,924 | 1,680 | 95,880,875 | 95,930,413 | 1,868 | Smaller |
| 2 | ANKRD36C | 95,887,998 | 95,888,091 | 94 | 95,880,875 | 95,930,413 | 1,868 | Smaller |
| 2 | ANKRD36C | 95,888,121 | 95,889,798 | 1,678 | 95,880,875 | 95,930,413 | 1,868 | Smaller |
| 2 | ANKRD36C | 95,889,872 | 95,889,965 | 94 | 95,880,875 | 95,930,413 | 1,868 | Smaller |
| 2 | ANKRD36C | 95,889,995 | 95,891,664 | 1,670 | 95,880,875 | 95,930,413 | 1,868 | Smaller |
| 2 | ANKRD36C | 95,891,738 | 95,891,831 | 94 | 95,880,875 | 95,930,413 | 1,868 | Smaller |
| 2 | ANKRD36C | 95,891,861 | 95,893,532 | 1,672 | 95,880,875 | 95,930,413 | 1,868 | Smaller |
| 2 | ANKRD36C | 95,891,861 | 95,899,144 | 7,284 | 95,880,875 | 95,930,413 | 1,868 | Larger |
| 2 | ANKRD36C | 95,893,606 | 95,893,695 | 90 | 95,880,875 | 95,930,413 | 1,868 | Smaller |
| 2 | ANKRD36C | 95,893,725 | 95,894,212 | 488 | 95,880,875 | 95,930,413 | 1,868 | Smaller |
| 2 | ANKRD36C | 95,893,725 | 95,895,398 | 1,674 | 95,880,875 | 95,930,413 | 1,868 | Smaller |
| 2 | ANKRD36C | 95,894,276 | 95,895,398 | 1,123 | 95,880,875 | 95,930,413 | 1,868 | Smaller |
| 2 | ANKRD36C | 95,895,472 | 95,895,561 | 90 | 95,880,875 | 95,930,413 | 1,868 | Smaller |
| 2 | ANKRD36C | 95,895,591 | 95,897,269 | 1,679 | 95,880,875 | 95,930,413 | 1,868 | Smaller |
| 2 | ANKRD36C | 95,895,591 | 95,902,885 | 7,295 | 95,880,875 | 95,930,413 | 1,868 | Larger |
| 2 | ANKRD36C | 95,897,343 | 95,897,436 | 94 | 95,880,875 | 95,930,413 | 1,868 | Smaller |
| 2 | ANKRD36C | 95,897,466 | 95,899,144 | 1,679 | 95,880,875 | 95,930,413 | 1,868 | Smaller |
| 2 | ANKRD36C | 95,899,218 | 95,899,307 | 90 | 95,880,875 | 95,930,413 | 1,868 | Smaller |
| 2 | ANKRD36C | 95,899,337 | 95,901,014 | 1,678 | 95,880,875 | 95,930,413 | 1,868 | Smaller |
| 2 | ANKRD36C | 95,899,337 | 95,912,243 | 12,907 | 95,880,875 | 95,930,413 | 1,868 | Larger |
| 2 | ANKRD36C | 95,901,088 | 95,901,177 | 90 | 95,880,875 | 95,930,413 | 1,868 | Smaller |
| 2 | ANKRD36C | 95,901,207 | 95,902,885 | 1,679 | 95,880,875 | 95,930,413 | 1,868 | Smaller |
| 2 | ANKRD36C | 95,902,959 | 95,903,052 | 94 | 95,880,875 | 95,930,413 | 1,868 | Smaller |
| 2 | ANKRD36C | 95,903,082 | 95,904,760 | 1,679 | 95,880,875 | 95,930,413 | 1,868 | Smaller |
| 2 | ANKRD36C | 95,904,834 | 95,904,923 | 90 | 95,880,875 | 95,930,413 | 1,868 | Smaller |
| 2 | ANKRD36C | 95,904,953 | 95,905,440 | 488 | 95,880,875 | 95,930,413 | 1,868 | Smaller |
| 2 | ANKRD36C | 95,904,953 | 95,906,630 | 1,678 | 95,880,875 | 95,930,413 | 1,868 | Smaller |
| 2 | ANKRD36C | 95,905,504 | 95,906,630 | 1,127 | 95,880,875 | 95,930,413 | 1,868 | Smaller |
| 2 | ANKRD36C | 95,906,704 | 95,906,793 | 90 | 95,880,875 | 95,930,413 | 1,868 | Smaller |
| 2 | ANKRD36C | 95,906,823 | 95,908,501 | 1,679 | 95,880,875 | 95,930,413 | 1,868 | Smaller |
| 2 | ANKRD36C | 95,908,575 | 95,908,668 | 94 | 95,880,875 | 95,930,413 | 1,868 | Smaller |
| 2 | ANKRD36C | 95,908,698 | 95,910,372 | 1,675 | 95,880,875 | 95,930,413 | 1,868 | Smaller |
| 2 | ANKRD36C | 95,910,446 | 95,910,539 | 94 | 95,880,875 | 95,930,413 | 1,868 | Smaller |
| 2 | ANKRD36C | 95,910,569 | 95,912,243 | 1,675 | 95,880,875 | 95,930,413 | 1,868 | Smaller |
| 2 | ANKRD36C | 95,912,317 | 95,912,406 | 90 | 95,880,875 | 95,930,413 | 1,868 | Smaller |
| 2 | ANKRD36C | 95,912,436 | 95,914,107 | 1,672 | 95,880,875 | 95,930,413 | 1,868 | Smaller |
| 2 | ANKRD36C | 95,914,181 | 95,914,274 | 94 | 95,880,875 | 95,930,413 | 1,868 | Smaller |
| 2 | ANKRD36C | 95,914,304 | 95,915,979 | 1,676 | 95,880,875 | 95,930,413 | 1,868 | Smaller |
| 2 | ANKRD36C | 95,916,053 | 95,916,142 | 90 | 95,880,875 | 95,930,413 | 1,868 | Smaller |
| 2 | ANKRD36C | 95,916,172 | 95,917,854 | 1,683 | 95,880,875 | 95,930,413 | 1,868 | Smaller |
| 2 | ANKRD36C | 95,917,928 | 95,918,013 | 86 | 95,880,875 | 95,930,413 | 1,868 | Smaller |
| 2 | ANKRD36C | 95,918,043 | 95,919,732 | 1,690 | 95,880,875 | 95,930,413 | 1,868 | Smaller |
| 2 | ANKRD36C | 95,918,043 | 95,921,606 | 3,564 | 95,880,875 | 95,930,413 | 1,868 | Larger |
| 2 | ANKRD36C | 95,919,806 | 95,919,891 | 86 | 95,880,875 | 95,930,413 | 1,868 | Smaller |
| 2 | ANKRD36C | 95,919,921 | 95,921,606 | 1,686 | 95,880,875 | 95,930,413 | 1,868 | Smaller |
| 2 | ANKRD36C | 95,921,680 | 95,921,781 | 102 | 95,880,875 | 95,930,413 | 1,868 | Smaller |
| 2 | ANKRD36C | 95,921,811 | 95,923,494 | 1,684 | 95,880,875 | 95,930,413 | 1,868 | Smaller |
| 2 | ANKRD36C | 95,923,568 | 95,923,660 | 93 | 95,880,875 | 95,930,413 | 1,868 | Smaller |
| 2 | ANKRD36C | 95,923,690 | 95,925,351 | 1,662 | 95,880,875 | 95,930,413 | 1,868 | Smaller |
| 2 | ANKRD36C | 95,925,425 | 95,925,518 | 94 | 95,880,875 | 95,930,413 | 1,868 | Smaller |
| 2 | ANKRD36C | 95,925,548 | 95,927,213 | 1,666 | 95,880,875 | 95,930,413 | 1,868 | Smaller |
| 2 | ANKRD36C | 95,927,287 | 95,927,380 | 94 | 95,880,875 | 95,930,413 | 1,868 | Smaller |
| 2 | ANKRD36C | 95,927,410 | 95,929,071 | 1,662 | 95,880,875 | 95,930,413 | 1,868 | Smaller |
| 2 | ANKRD36C | 95,929,145 | 95,929,238 | 94 | 95,880,875 | 95,930,413 | 1,868 | Smaller |
| 2 | ANKRD36C | 95,958,664 | 95,958,756 | 93 | 95,957,271 | 95,963,698 | 1,879 | Smaller |
| 2 | ANKRD36C | 95,958,786 | 95,960,472 | 1,687 | 95,957,271 | 95,963,698 | 1,879 | Smaller |
| 2 | ANKRD36C | 95,960,546 | 95,960,638 | 93 | 95,957,271 | 95,963,698 | 1,879 | Smaller |
| 2 | ANKRD36C | 95,960,668 | 95,962,351 | 1,684 | 95,957,271 | 95,963,698 | 1,879 | Smaller |
| 2 | ANKRD36C | 95,962,425 | 95,962,518 | 94 | 95,957,271 | 95,963,698 | 1,879 | Smaller |
| 2 | ANKRD36 | 97,142,669 | 97,142,762 | 94 | 97,141,489 | 97,146,014 | 1,868 | Smaller |
| 2 | ANKRD36 | 97,142,836 | 97,144,517 | 1,682 | 97,141,489 | 97,146,014 | 1,868 | Smaller |
| 2 | ANKRD36 | 97,144,547 | 97,144,639 | 93 | 97,141,489 | 97,146,014 | 1,868 | Smaller |
| 2 | ANKRD36 | 97,179,767 | 97,179,860 | 94 | 97,178,842 | 97,218,766 | 1,871 | Smaller |
| 2 | ANKRD36 | 97,179,934 | 97,181,597 | 1,664 | 97,178,842 | 97,218,766 | 1,871 | Smaller |
| 2 | ANKRD36 | 97,181,627 | 97,181,720 | 94 | 97,178,842 | 97,218,766 | 1,871 | Smaller |
| 2 | ANKRD36 | 97,181,794 | 97,183,458 | 1,665 | 97,178,842 | 97,218,766 | 1,871 | Smaller |
| 2 | ANKRD36 | 97,183,488 | 97,183,581 | 94 | 97,178,842 | 97,218,766 | 1,871 | Smaller |
| 2 | ANKRD36 | 97,183,655 | 97,185,315 | 1,661 | 97,178,842 | 97,218,766 | 1,871 | Smaller |
| 2 | ANKRD36 | 97,185,345 | 97,185,437 | 93 | 97,178,842 | 97,218,766 | 1,871 | Smaller |
| 2 | ANKRD36 | 97,185,511 | 97,187,197 | 1,687 | 97,178,842 | 97,218,766 | 1,871 | Smaller |
| 2 | ANKRD36 | 97,187,227 | 97,187,328 | 102 | 97,178,842 | 97,218,766 | 1,871 | Smaller |
| 2 | ANKRD36 | 97,187,402 | 97,189,086 | 1,685 | 97,178,842 | 97,218,766 | 1,871 | Smaller |
| 2 | ANKRD36 | 97,189,116 | 97,189,217 | 102 | 97,178,842 | 97,218,766 | 1,871 | Smaller |
| 2 | ANKRD36 | 97,189,291 | 97,190,977 | 1,687 | 97,178,842 | 97,218,766 | 1,871 | Smaller |
| 2 | ANKRD36 | 97,191,007 | 97,191,108 | 102 | 97,178,842 | 97,218,766 | 1,871 | Smaller |
| 2 | ANKRD36 | 97,191,182 | 97,192,857 | 1,676 | 97,178,842 | 97,218,766 | 1,871 | Smaller |
| 2 | ANKRD36 | 97,192,887 | 97,192,980 | 94 | 97,178,842 | 97,218,766 | 1,871 | Smaller |
| 2 | ANKRD36 | 97,193,054 | 97,194,725 | 1,672 | 97,178,842 | 97,218,766 | 1,871 | Smaller |
| 2 | ANKRD36 | 97,194,755 | 97,194,844 | 90 | 97,178,842 | 97,218,766 | 1,871 | Smaller |
| 2 | ANKRD36 | 97,194,918 | 97,196,592 | 1,675 | 97,178,842 | 97,218,766 | 1,871 | Smaller |
| 2 | ANKRD36 | 97,196,622 | 97,196,715 | 94 | 97,178,842 | 97,218,766 | 1,871 | Smaller |
| 2 | ANKRD36 | 97,196,789 | 97,198,462 | 1,674 | 97,178,842 | 97,218,766 | 1,871 | Smaller |
| 2 | ANKRD36 | 97,198,492 | 97,198,585 | 94 | 97,178,842 | 97,218,766 | 1,871 | Smaller |
| 2 | ANKRD36 | 97,198,659 | 97,200,333 | 1,675 | 97,178,842 | 97,218,766 | 1,871 | Smaller |
| 2 | ANKRD36 | 97,200,363 | 97,200,452 | 90 | 97,178,842 | 97,218,766 | 1,871 | Smaller |
| 2 | ANKRD36 | 97,200,526 | 97,202,201 | 1,676 | 97,178,842 | 97,218,766 | 1,871 | Smaller |
| 2 | ANKRD36 | 97,202,231 | 97,202,320 | 90 | 97,178,842 | 97,218,766 | 1,871 | Smaller |
| 2 | ANKRD36 | 97,202,394 | 97,204,067 | 1,674 | 97,178,842 | 97,218,766 | 1,871 | Smaller |
| 2 | ANKRD36 | 97,204,097 | 97,204,190 | 94 | 97,178,842 | 97,218,766 | 1,871 | Smaller |
| 2 | ANKRD36 | 97,204,264 | 97,205,939 | 1,676 | 97,178,842 | 97,218,766 | 1,871 | Smaller |
| 2 | ANKRD36 | 97,205,969 | 97,206,062 | 94 | 97,178,842 | 97,218,766 | 1,871 | Smaller |
| 2 | ANKRD36 | 97,206,136 | 97,207,810 | 1,675 | 97,178,842 | 97,218,766 | 1,871 | Smaller |
| 2 | ANKRD36 | 97,207,840 | 97,207,933 | 94 | 97,178,842 | 97,218,766 | 1,871 | Smaller |
| 2 | ANKRD36 | 97,208,007 | 97,209,680 | 1,674 | 97,178,842 | 97,218,766 | 1,871 | Smaller |
| 2 | ANKRD36 | 97,209,710 | 97,209,799 | 90 | 97,178,842 | 97,218,766 | 1,871 | Smaller |
| 2 | ANKRD36 | 97,209,873 | 97,211,545 | 1,673 | 97,178,842 | 97,218,766 | 1,871 | Smaller |
| 2 | ANKRD36 | 97,211,575 | 97,211,668 | 94 | 97,178,842 | 97,218,766 | 1,871 | Smaller |
| 2 | ANKRD36 | 97,211,742 | 97,213,418 | 1,677 | 97,178,842 | 97,218,766 | 1,871 | Smaller |
| 2 | ANKRD36 | 97,213,448 | 97,213,541 | 94 | 97,178,842 | 97,218,766 | 1,871 | Smaller |
| 2 | ANKRD36 | 97,213,615 | 97,215,300 | 1,686 | 97,178,842 | 97,218,766 | 1,871 | Smaller |
| 2 | ANKRD36 | 97,215,330 | 97,215,424 | 95 | 97,178,842 | 97,218,766 | 1,871 | Smaller |
| 2 | ANKRD36 | 97,215,498 | 97,217,176 | 1,679 | 97,178,842 | 97,218,766 | 1,871 | Smaller |
| 2 | ANKRD36 | 97,217,206 | 97,217,299 | 94 | 97,178,842 | 97,218,766 | 1,871 | Smaller |
| 2 | ANKRD36B | 97,538,241 | 97,538,334 | 94 | 97,536,521 | 97,562,006 | 1,872 | Smaller |
| 2 | ANKRD36B | 97,538,364 | 97,540,033 | 1,670 | 97,536,521 | 97,562,006 | 1,872 | Smaller |
| 2 | ANKRD36B | 97,540,107 | 97,540,200 | 94 | 97,536,521 | 97,562,006 | 1,872 | Smaller |
| 2 | ANKRD36B | 97,540,230 | 97,541,910 | 1,681 | 97,536,521 | 97,562,006 | 1,872 | Smaller |
| 2 | ANKRD36B | 97,541,984 | 97,542,078 | 95 | 97,536,521 | 97,562,006 | 1,872 | Smaller |
| 2 | ANKRD36B | 97,542,108 | 97,543,789 | 1,682 | 97,536,521 | 97,562,006 | 1,872 | Smaller |
| 2 | ANKRD36B | 97,543,863 | 97,543,956 | 94 | 97,536,521 | 97,562,006 | 1,872 | Smaller |
| 2 | ANKRD36B | 97,543,986 | 97,545,665 | 1,680 | 97,536,521 | 97,562,006 | 1,872 | Smaller |
| 2 | ANKRD36B | 97,545,739 | 97,545,832 | 94 | 97,536,521 | 97,562,006 | 1,872 | Smaller |
| 2 | ANKRD36B | 97,545,862 | 97,547,535 | 1,674 | 97,536,521 | 97,562,006 | 1,872 | Smaller |
| 2 | ANKRD36B | 97,547,609 | 97,547,702 | 94 | 97,536,521 | 97,562,006 | 1,872 | Smaller |
| 2 | ANKRD36B | 97,547,732 | 97,549,418 | 1,687 | 97,536,521 | 97,562,006 | 1,872 | Smaller |
| 2 | ANKRD36B | 97,549,492 | 97,549,585 | 94 | 97,536,521 | 97,562,006 | 1,872 | Smaller |
| 2 | ANKRD36B | 97,549,615 | 97,551,288 | 1,674 | 97,536,521 | 97,562,006 | 1,872 | Smaller |
| 2 | ANKRD36B | 97,551,362 | 97,551,451 | 90 | 97,536,521 | 97,562,006 | 1,872 | Smaller |
| 2 | ANKRD36B | 97,551,481 | 97,553,167 | 1,687 | 97,536,521 | 97,562,006 | 1,872 | Smaller |
| 2 | ANKRD36B | 97,553,241 | 97,553,342 | 102 | 97,536,521 | 97,562,006 | 1,872 | Smaller |
| 2 | ANKRD36B | 97,553,372 | 97,555,059 | 1,688 | 97,536,521 | 97,562,006 | 1,872 | Smaller |
| 2 | ANKRD36B | 97,555,133 | 97,555,225 | 93 | 97,536,521 | 97,562,006 | 1,872 | Smaller |
| 2 | ANKRD36B | 97,555,255 | 97,556,936 | 1,682 | 97,536,521 | 97,562,006 | 1,872 | Smaller |
| 2 | ANKRD36B | 97,557,010 | 97,557,103 | 94 | 97,536,521 | 97,562,006 | 1,872 | Smaller |
| 2 | ANKRD36B | 97,557,133 | 97,558,798 | 1,666 | 97,536,521 | 97,562,006 | 1,872 | Smaller |
| 2 | ANKRD36B | 97,558,872 | 97,558,965 | 94 | 97,536,521 | 97,562,006 | 1,872 | Smaller |
| 2 | ANKRD36B | 97,558,995 | 97,560,664 | 1,670 | 97,536,521 | 97,562,006 | 1,872 | Smaller |
| 2 | ANKRD36B | 97,560,738 | 97,560,831 | 94 | 97,536,521 | 97,562,006 | 1,872 | Smaller |
| 3 | RPL23AP93 | 194,563,071 | 194,563,201 | 131 | 194,563,042 | 194,563,236 | 65 | Larger |
| 3 | ENSG00000228028 | 196,250,663 | 196,251,373 | 711 | 196,249,758 | 196,251,390 | 27 | Larger |
| 3 | ENSG00000228028 | 196,250,690 | 196,251,373 | 684 | 196,249,758 | 196,251,390 | 27 | Larger |
| 3 | LMLN-AS1 | 198,038,859 | 198,039,146 | 288 | 198,038,838 | 198,039,152 | 62 | Larger |
| 3 | LMLN-AS1 | 198,038,859 | 198,039,146 | 288 | 198,038,840 | 198,039,152 | 21 | Larger |
| 3 | PFKFB4 | 48,561,669 | 48,561,759 | 91 | 48,561,634 | 48,561,816 | 91 | Equal |
| 4 | CTBP1 | 1,237,265 | 1,237,474 | 210 | 1,236,521 | 1,238,041 | 70 | Larger |
| 4 | CTBP1 | 1,237,265 | 1,237,474 | 210 | 1,237,062 | 1,238,041 | 35 | Larger |
| 4 | TMEM129 | 1,720,946 | 1,721,029 | 84 | 1,720,931 | 1,721,142 | 42 | Larger |
| 4 | ENSG00000250658 | 187,304,468 | 187,304,637 | 170 | 187,304,399 | 187,305,149 | 34 | Larger |
| 4 | AMBN | 70,602,663 | 70,602,797 | 135 | 70,602,572 | 70,603,036 | 174 | Smaller |
| 4 | AMBN | 70,602,837 | 70,602,971 | 135 | 70,602,572 | 70,603,036 | 174 | Smaller |
| 4 | ART3 | 76,100,825 | 76,100,989 | 165 | 76,100,646 | 76,101,084 | 194 | Smaller |
| 5 | ENSG00000273957 | 100,389,148 | 100,389,471 | 324 | 100,388,958 | 100,389,600 | 324 | Equal |
| 5 | FAM153A | 177,718,637 | 177,718,725 | 89 | 177,718,608 | 177,718,750 | 47 | Larger |
| 5 | FAM153A | 177,718,637 | 177,718,725 | 89 | 177,718,608 | 177,718,838 | 90 | Smaller |
| 5 | FAM153A | 177,718,637 | 177,718,725 | 89 | 177,718,624 | 177,718,765 | 45 | Larger |
| 5 | PLCXD3 | 41,312,539 | 41,312,728 | 190 | 41,312,535 | 41,312,736 | 4 | Larger |
| 5 | BRD9 | 882,546 | 882,955 | 410 | 882,230 | 883,137 | 31 | Larger |
| 5 | BRD9 | 882,546 | 882,955 | 410 | 882,232 | 883,133 | 117 | Larger |
| 5 | BRD9 | 882,546 | 882,955 | 410 | 882,273 | 883,153 | 96 | Larger |
| 5 | BRD9 | 882,546 | 882,955 | 410 | 882,279 | 883,137 | 61 | Larger |
| 5 | BRD9 | 882,546 | 882,955 | 410 | 882,390 | 882,996 | 186 | Larger |
| 6 | WDR27 | 169,665,011 | 169,665,134 | 124 | 169,664,851 | 169,665,154 | 62 | Larger |
| 6 | ENSG00000236173 | 169,809,494 | 169,809,584 | 91 | 169,808,137 | 169,809,681 | 14 | Larger |
| 6 | ENSG00000225532 | 170,737,552 | 170,737,611 | 60 | 170,737,542 | 170,737,696 | 59 | Larger |
| 6 | HCG22 | 31,056,517 | 31,056,520 | 4 | 31,056,326 | 31,056,747 | 33 | Smaller |
| 6 | TAPBP | 33,300,416 | 33,300,720 | 305 | 33,300,381 | 33,300,974 | 304 | Larger |
| 6 | ENSG00000225096 | 58,436,705 | 58,437,243 | 539 | 58,432,485 | 58,444,700 | 171 | Larger |
| 6 | ENSG00000225096 | 58,436,705 | 58,437,243 | 539 | 58,432,488 | 58,444,696 | 511 | Larger |
| 7 | SPDYE3 | 100,309,193 | 100,310,359 | 1,167 | 100,308,214 | 100,315,233 | 1,390 | Smaller |
| 7 | SPDYE3 | 100,310,579 | 100,311,749 | 1,171 | 100,308,214 | 100,315,233 | 1,390 | Smaller |
| 7 | SPDYE3 | 100,311,969 | 100,313,139 | 1,171 | 100,308,214 | 100,315,233 | 1,390 | Smaller |
| 7 | SPDYE3 | 100,313,359 | 100,314,531 | 1,173 | 100,308,214 | 100,315,233 | 1,390 | Smaller |
| 7 | ENSG00000241449 | 150,040,655 | 150,041,319 | 665 | 150,040,010 | 150,041,613 | 743 | Smaller |
| 7 | ENSG00000241449 | 150,040,655 | 150,041,362 | 708 | 150,040,010 | 150,041,613 | 743 | Smaller |
| 7 | ENSG00000241449 | 150,040,716 | 150,041,362 | 647 | 150,040,010 | 150,041,613 | 743 | Smaller |
| 7 | ENSG00000241449 | 150,040,911 | 150,041,362 | 452 | 150,040,010 | 150,041,613 | 743 | Smaller |
| 7 | ENSG00000203335 | 154,059,470 | 154,060,509 | 1,040 | 154,059,003 | 154,062,545 | 1,595 | Smaller |
| 7 | ENSG00000203335 | 154,059,470 | 154,060,976 | 1,507 | 154,059,003 | 154,062,545 | 1,595 | Smaller |
| 7 | ENSG00000203335 | 154,059,513 | 154,060,976 | 1,464 | 154,059,003 | 154,062,545 | 1,595 | Smaller |
| 7 | DPY19L2P3 | 29,650,408 | 29,650,717 | 310 | 29,650,330 | 29,650,726 | 99 | Larger |
| 7 | ENSG00000236510 | 3,643,352 | 3,643,465 | 114 | 3,643,041 | 3,643,804 | 57 | Larger |
| 7 | ENSG00000279072 | 56,813,629 | 56,815,466 | 1,838 | 56,811,890 | 56,817,296 | 1,742 | Larger |
| 7 | ENSG00000279072 | 56,813,629 | 56,815,466 | 1,838 | 56,813,581 | 56,827,943 | 1,838 | Equal |
| 7 | ENSG00000279072 | 56,815,589 | 56,815,591 | 3 | 56,811,890 | 56,817,296 | 1,742 | Smaller |
| 7 | ENSG00000279072 | 56,815,589 | 56,815,591 | 3 | 56,813,581 | 56,827,943 | 1,838 | Smaller |
| 7 | ENSG00000279072 | 56,817,161 | 56,817,352 | 192 | 56,813,581 | 56,827,943 | 1,838 | Smaller |
| 7 | ENSG00000279072 | 56,817,161 | 56,817,352 | 192 | 56,817,113 | 56,817,440 | 48 | Larger |
| 7 | ENSG00000279072 | 56,817,475 | 56,817,477 | 3 | 56,813,581 | 56,827,943 | 1,838 | Smaller |
| 7 | ENSG00000279072 | 56,819,047 | 56,819,190 | 144 | 56,813,581 | 56,827,943 | 1,838 | Smaller |
| 7 | ENSG00000279072 | 56,819,047 | 56,819,190 | 144 | 56,818,999 | 56,819,278 | 48 | Larger |
| 7 | ENSG00000279072 | 56,819,047 | 56,819,190 | 144 | 56,818,999 | 56,823,090 | 1,982 | Smaller |
| 7 | ENSG00000279072 | 56,819,313 | 56,819,315 | 3 | 56,813,581 | 56,827,943 | 1,838 | Smaller |
| 7 | ENSG00000279072 | 56,819,313 | 56,819,315 | 3 | 56,818,999 | 56,823,090 | 1,982 | Smaller |
| 7 | ENSG00000279072 | 56,820,885 | 56,821,172 | 288 | 56,813,581 | 56,827,943 | 1,838 | Smaller |
| 7 | ENSG00000279072 | 56,820,885 | 56,821,172 | 288 | 56,818,999 | 56,823,090 | 1,982 | Smaller |
| 7 | ENSG00000279072 | 56,820,885 | 56,821,172 | 288 | 56,820,837 | 56,821,260 | 48 | Larger |
| 7 | ENSG00000279072 | 56,821,295 | 56,821,297 | 3 | 56,813,581 | 56,827,943 | 1,838 | Smaller |
| 7 | ENSG00000279072 | 56,821,295 | 56,821,297 | 3 | 56,818,999 | 56,823,090 | 1,982 | Smaller |
| 7 | SPDYE5 | 75,495,375 | 75,496,673 | 1,299 | 75,494,516 | 75,497,318 | 1,505 | Smaller |
| 7 | MAGI2-AS3 | 79,459,454 | 79,459,535 | 82 | 79,459,307 | 79,459,550 | 129 | Smaller |
| 8 | ENSG00000254160 | 1,246,859 | 1,247,005 | 147 | 1,246,781 | 1,248,651 | 49 | Larger |
| 8 | ENSG00000254160 | 1,247,104 | 1,248,482 | 1,379 | 1,246,781 | 1,248,651 | 49 | Larger |
| 8 | ENSG00000253307 | 141,252,571 | 141,253,180 | 610 | 141,252,356 | 141,253,384 | 36 | Larger |
| 8 | ENSG00000271959 | 141,437,066 | 141,437,325 | 260 | 141,436,990 | 141,437,330 | 37 | Larger |
| 8 | BOP1 | 144,262,789 | 144,262,852 | 64 | 144,262,708 | 144,262,865 | 35 | Larger |
| 8 | BOP1 | 144,262,789 | 144,262,852 | 64 | 144,262,743 | 144,262,870 | 66 | Smaller |
| 8 | BOP1 | 144,262,789 | 144,262,852 | 64 | 144,262,775 | 144,262,869 | 29 | Larger |
| 8 | NEFM | 24,917,732 | 24,917,848 | 117 | 24,917,689 | 24,917,922 | 39 | Larger |
| 8 | ZBTB10 | 80,518,439 | 80,518,510 | 72 | 80,518,401 | 80,518,538 | 36 | Larger |
| 9 | PMPCA | 136,418,196 | 136,418,437 | 242 | 136,418,151 | 136,418,551 | 61 | Larger |
| 9 | PMPCA | 136,418,196 | 136,418,437 | 242 | 136,418,157 | 136,418,551 | 183 | Larger |
| 9 | CSNK1G2P1 | 5,041,592 | 5,041,632 | 41 | 5,041,572 | 5,041,660 | 39 | Larger |
| 9 | LINC03025 | 68,322,947 | 68,323,026 | 80 | 68,322,082 | 68,323,229 | 79 | Larger |
| 9 | LINC03025 | 68,322,947 | 68,323,026 | 80 | 68,322,098 | 68,323,229 | 236 | Smaller |
| 10 | ENSG00000229206 | 11,030,489 | 11,030,590 | 102 | 11,030,372 | 11,030,643 | 34 | Larger |
| 10 | ENSG00000229206 | 11,030,489 | 11,030,590 | 102 | 11,030,374 | 11,030,640 | 102 | Equal |
| 10 | ENSG00000228302 | 12,247,109 | 12,247,412 | 304 | 12,246,846 | 12,247,542 | 34 | Larger |
| 10 | RPL19P16 | 121,133,355 | 121,133,400 | 46 | 121,133,317 | 121,133,409 | 45 | Larger |
| 10 | DMBT1 | 122,570,938 | 122,572,313 | 1,376 | 122,570,305 | 122,573,866 | 1,407 | Smaller |
| 10 | DMBT1 | 122,572,362 | 122,573,714 | 1,353 | 122,570,305 | 122,573,866 | 1,407 | Smaller |
| 10 | NPM1P31 | 124,868,003 | 124,868,055 | 53 | 124,867,988 | 124,868,101 | 18 | Larger |
| 10 | NPM1P31 | 124,868,003 | 124,868,055 | 53 | 124,867,988 | 124,868,107 | 3 | Larger |
| 10 | ENSG00000276662 | 148,228 | 148,443 | 216 | 148,212 | 148,468 | 28 | Larger |
| 10 | LINC02645 | 2,501,369 | 2,501,494 | 126 | 2,501,302 | 2,501,932 | 63 | Larger |
| 10 | LINC02645 | 2,501,558 | 2,501,746 | 189 | 2,501,302 | 2,501,932 | 63 | Larger |
| 10 | LINC02645 | 2,501,684 | 2,501,809 | 126 | 2,501,302 | 2,501,932 | 63 | Larger |
| 10 | LINC02645 | 2,501,810 | 2,501,872 | 63 | 2,501,302 | 2,501,932 | 63 | Equal |
| 10 | CCDC7 | 32,845,327 | 32,845,542 | 216 | 32,844,905 | 32,845,787 | 298 | Smaller |
| 10 | ANTXRL | 46,329,745 | 46,329,814 | 70 | 46,329,640 | 46,329,875 | 60 | Larger |
| 10 | ANTXRL | 46,329,745 | 46,329,814 | 70 | 46,329,733 | 46,329,869 | 69 | Larger |
| 10 | C10orf53 | 49,709,967 | 49,710,006 | 40 | 49,709,944 | 49,710,022 | 14 | Larger |
| 10 | C10orf53 | 49,709,967 | 49,710,006 | 40 | 49,709,946 | 49,710,022 | 2 | Larger |
| 10 | SDCBPP1 | 6,293,946 | 6,293,968 | 23 | 6,293,928 | 6,293,989 | 31 | Smaller |
| 11 | MRPL23 | 1,956,449 | 1,956,541 | 93 | 1,956,359 | 1,956,563 | 93 | Equal |
| 11 | ZPR1 | 116,775,334 | 116,775,502 | 169 | 116,775,334 | 116,775,649 | 168 | Larger |
| 11 | GLB1L2 | 134,375,885 | 134,376,094 | 210 | 134,375,741 | 134,376,161 | 70 | Larger |
| 11 | ENSG00000285562 | 18,143,672 | 18,143,758 | 87 | 18,143,652 | 18,143,762 | 29 | Larger |
| 11 | SIRT3 | 223,850 | 224,065 | 216 | 223,787 | 224,074 | 72 | Larger |
| 11 | IFITM2 | 309,185 | 309,252 | 68 | 309,182 | 309,317 | 68 | Equal |
| 11 | IFITM3 | 321,387 | 322,595 | 1,209 | 321,339 | 322,621 | 39 | Larger |
| 11 | ENSG00000255089 | 322,224 | 322,379 | 156 | 321,339 | 322,621 | 39 | Larger |
| 11 | IFITM3 | 322,401 | 322,556 | 156 | 321,339 | 322,621 | 39 | Larger |
| 11 | IGHMBP2 | 68,929,844 | 68,929,891 | 48 | 68,929,807 | 68,929,955 | 48 | Equal |
| 11 | ENSG00000250397 | 858,692 | 859,546 | 855 | 858,477 | 859,654 | 16 | Larger |
| 11 | CCDC82 | 96,384,294 | 96,384,362 | 69 | 96,384,235 | 96,384,375 | 69 | Equal |
| 12 | SELPLG | 108,623,888 | 108,623,917 | 30 | 108,623,526 | 108,623,945 | 30 | Equal |
| 12 | PRB3 | 11,267,519 | 11,267,644 | 126 | 11,267,299 | 11,268,161 | 126 | Equal |
| 12 | PRB3 | 11,267,519 | 11,267,644 | 126 | 11,267,302 | 11,268,161 | 188 | Smaller |
| 12 | PRB3 | 11,267,519 | 11,267,644 | 126 | 11,267,400 | 11,268,161 | 63 | Larger |
| 12 | PRB4 | 11,308,442 | 11,308,648 | 207 | 11,308,351 | 11,308,900 | 63 | Larger |
| 12 | PRB1 | 11,353,331 | 11,353,789 | 459 | 11,353,208 | 11,354,010 | 123 | Larger |
| 12 | PRB1 | 11,353,331 | 11,353,789 | 459 | 11,353,210 | 11,354,010 | 182 | Larger |
| 12 | PRB1 | 11,353,391 | 11,353,789 | 399 | 11,353,208 | 11,354,010 | 123 | Larger |
| 12 | PRB1 | 11,353,391 | 11,353,789 | 399 | 11,353,210 | 11,354,010 | 182 | Larger |
| 12 | RBM19 | 113,817,926 | 113,818,102 | 177 | 113,817,817 | 113,818,123 | 134 | Larger |
| 12 | UBC | 124,912,181 | 124,913,320 | 1,140 | 124,911,739 | 124,913,771 | 228 | Larger |
| 12 | UBC | 124,913,268 | 124,913,495 | 228 | 124,911,739 | 124,913,771 | 228 | Equal |
| 12 | ENSG00000278356 | 2,885,898 | 2,885,952 | 55 | 2,885,867 | 2,885,980 | 55 | Equal |
| 12 | TROAP | 49,330,574 | 49,330,660 | 87 | 49,330,378 | 49,330,710 | 99 | Smaller |
| 12 | ENSG00000257740 | 56,309,175 | 56,309,319 | 145 | 56,309,132 | 56,309,406 | 144 | Larger |
| 12 | ENSG00000255825 | 590,773 | 591,020 | 248 | 590,659 | 591,094 | 62 | Larger |
| 12 | ENSG00000287577 | 9,349,162 | 9,349,241 | 80 | 9,349,001 | 9,349,425 | 40 | Larger |
| 12 | ENSG00000284634 | 9,567,568 | 9,569,057 | 1,490 | 9,566,366 | 9,569,494 | 1,540 | Smaller |
| 13 | ZDHHC20-IT1 | 21,377,209 | 21,377,666 | 458 | 21,377,192 | 21,377,695 | 42 | Larger |
| 13 | TPT1-AS1 | 45,378,672 | 45,379,194 | 523 | 45,378,031 | 45,380,043 | 663 | Smaller |
| 13 | TPT1-AS1 | 45,378,672 | 45,379,925 | 1,254 | 45,378,031 | 45,380,043 | 663 | Larger |
| 13 | TPT1-AS1 | 45,379,336 | 45,379,898 | 563 | 45,378,031 | 45,380,043 | 663 | Smaller |
| 13 | TPT1-AS1 | 45,379,336 | 45,379,925 | 590 | 45,378,031 | 45,380,043 | 663 | Smaller |
| 13 | TPT1-AS1 | 45,383,170 | 45,383,759 | 590 | 45,382,591 | 45,383,975 | 668 | Smaller |
| 13 | TPT1-AS1 | 45,383,236 | 45,383,759 | 524 | 45,382,591 | 45,383,975 | 668 | Smaller |
| 14 | IGHG3 | 105,770,150 | 105,770,292 | 143 | 105,769,997 | 105,770,744 | 188 | Smaller |
| 14 | IGHG3 | 105,770,338 | 105,770,480 | 143 | 105,769,997 | 105,770,744 | 188 | Smaller |
| 14 | IGHG3 | 105,770,526 | 105,770,668 | 143 | 105,769,997 | 105,770,744 | 188 | Smaller |
| 14 | RBM23 | 22,913,822 | 22,913,914 | 93 | 22,913,686 | 22,914,033 | 175 | Smaller |
| 14 | CGRRF1 | 54,530,227 | 54,530,612 | 386 | 54,530,139 | 54,530,849 | 179 | Larger |
| 14 | PAPLN | 73,263,783 | 73,263,941 | 159 | 73,263,771 | 73,264,064 | 100 | Larger |
| 14 | PAPLN | 73,263,783 | 73,263,941 | 159 | 73,263,771 | 73,264,065 | 34 | Larger |
| 14 | PAPLN | 73,263,783 | 73,264,009 | 227 | 73,263,771 | 73,264,064 | 100 | Larger |
| 14 | PAPLN | 73,263,783 | 73,264,009 | 227 | 73,263,771 | 73,264,065 | 34 | Larger |
| 14 | ISM2 | 77,484,566 | 77,484,676 | 111 | 77,484,293 | 77,485,006 | 354 | Smaller |
| 15 | PRKXP1 | 100,554,321 | 100,558,624 | 4,304 | 100,554,293 | 100,558,659 | 36 | Larger |
| 15 | SNHG14 | 25,086,465 | 25,087,466 | 1,002 | 25,086,078 | 25,090,066 | 1,130 | Smaller |
| 15 | SNHG14 | 25,086,615 | 25,087,466 | 852 | 25,086,078 | 25,090,066 | 1,130 | Smaller |
| 15 | SNHG14 | 25,087,600 | 25,089,613 | 2,014 | 25,086,078 | 25,090,066 | 1,130 | Larger |
| 15 | SNHG14 | 25,087,600 | 25,089,911 | 2,312 | 25,086,078 | 25,090,066 | 1,130 | Larger |
| 15 | SNHG14 | 25,181,896 | 25,182,018 | 123 | 25,180,980 | 25,184,975 | 1,913 | Smaller |
| 15 | SNHG14 | 25,182,106 | 25,182,196 | 91 | 25,180,980 | 25,184,975 | 1,913 | Smaller |
| 15 | SNHG14 | 25,182,329 | 25,182,711 | 383 | 25,180,980 | 25,184,975 | 1,913 | Smaller |
| 15 | SNHG14 | 25,182,755 | 25,183,940 | 1,186 | 25,180,980 | 25,184,975 | 1,913 | Smaller |
| 15 | SNHG14 | 25,236,454 | 25,239,649 | 3,196 | 25,235,042 | 25,250,054 | 1,874 | Larger |
| 15 | SNHG14 | 25,236,454 | 25,240,167 | 3,714 | 25,235,042 | 25,250,054 | 1,874 | Larger |
| 15 | SNHG14 | 25,239,782 | 25,240,167 | 386 | 25,235,042 | 25,250,054 | 1,874 | Smaller |
| 15 | SNHG14 | 25,240,213 | 25,241,399 | 1,187 | 25,235,042 | 25,250,054 | 1,874 | Smaller |
| 15 | SNHG14 | 25,240,213 | 25,241,558 | 1,346 | 25,235,042 | 25,250,054 | 1,874 | Smaller |
| 15 | SNHG14 | 25,241,691 | 25,242,071 | 381 | 25,235,042 | 25,250,054 | 1,874 | Smaller |
| 15 | SNHG14 | 25,242,117 | 25,243,426 | 1,310 | 25,235,042 | 25,250,054 | 1,874 | Smaller |
| 15 | SNHG14 | 25,243,558 | 25,243,939 | 382 | 25,235,042 | 25,250,054 | 1,874 | Smaller |
| 15 | SNHG14 | 25,243,985 | 25,244,134 | 150 | 25,235,042 | 25,250,054 | 1,874 | Smaller |
| 15 | SNHG14 | 25,243,985 | 25,245,137 | 1,153 | 25,235,042 | 25,250,054 | 1,874 | Smaller |
| 15 | SNHG14 | 25,244,272 | 25,245,137 | 866 | 25,235,042 | 25,250,054 | 1,874 | Smaller |
| 15 | SNHG14 | 25,245,210 | 25,245,288 | 79 | 25,235,042 | 25,250,054 | 1,874 | Smaller |
| 15 | SNHG14 | 25,245,422 | 25,245,805 | 384 | 25,235,042 | 25,250,054 | 1,874 | Smaller |
| 15 | SNHG14 | 25,245,851 | 25,247,156 | 1,306 | 25,235,042 | 25,250,054 | 1,874 | Smaller |
| 15 | SNHG14 | 25,247,289 | 25,247,671 | 383 | 25,235,042 | 25,250,054 | 1,874 | Smaller |
| 15 | SNHG14 | 25,247,290 | 25,247,671 | 382 | 25,235,042 | 25,250,054 | 1,874 | Smaller |
| 15 | SNHG14 | 25,247,717 | 25,247,866 | 150 | 25,235,042 | 25,250,054 | 1,874 | Smaller |
| 15 | SNHG14 | 25,247,717 | 25,249,013 | 1,297 | 25,235,042 | 25,250,054 | 1,874 | Smaller |
| 15 | GOLGA6L10 | 82,344,904 | 82,345,152 | 249 | 82,344,888 | 82,345,272 | 21 | Larger |
| 16 | NPIPB4 | 21,835,518 | 21,835,630 | 113 | 21,835,087 | 21,836,521 | 126 | Smaller |
| 16 | NPIPB4 | 21,835,518 | 21,835,630 | 113 | 21,835,087 | 21,837,565 | 252 | Smaller |
| 16 | NPIPB4 | 21,835,866 | 21,836,004 | 139 | 21,835,087 | 21,836,521 | 126 | Larger |
| 16 | NPIPB4 | 21,835,866 | 21,836,004 | 139 | 21,835,087 | 21,837,565 | 252 | Smaller |
| 16 | NPIPB4 | 21,835,866 | 21,836,004 | 139 | 21,835,833 | 21,836,287 | 69 | Larger |
| 16 | ENSG00000263326 | 25,071,910 | 25,072,471 | 562 | 25,071,868 | 25,072,471 | 89 | Larger |
| 16 | ENSG00000261766 | 28,862,531 | 28,863,161 | 631 | 28,862,303 | 28,863,239 | 314 | Larger |
| 16 | ZNF213 | 3,142,354 | 3,142,463 | 110 | 3,142,294 | 3,142,686 | 22 | Larger |
| 16 | ZNF213 | 3,142,354 | 3,142,463 | 110 | 3,142,295 | 3,142,686 | 44 | Larger |
| 16 | ENSG00000282924 | 33,741,854 | 33,741,947 | 94 | 33,740,453 | 33,746,882 | 1,879 | Smaller |
| 16 | ENSG00000282924 | 33,741,977 | 33,743,654 | 1,678 | 33,740,453 | 33,746,882 | 1,879 | Smaller |
| 16 | ENSG00000282924 | 33,743,728 | 33,743,821 | 94 | 33,740,453 | 33,746,882 | 1,879 | Smaller |
| 16 | ENSG00000282924 | 33,743,851 | 33,745,535 | 1,685 | 33,740,453 | 33,746,882 | 1,879 | Smaller |
| 16 | ENSG00000282924 | 33,745,609 | 33,745,702 | 94 | 33,740,453 | 33,746,882 | 1,879 | Smaller |
| 16 | DECR2 | 406,778 | 406,833 | 56 | 406,746 | 406,917 | 56 | Equal |
| 16 | DECR2 | 406,834 | 406,889 | 56 | 406,746 | 406,917 | 56 | Equal |
| 16 | CKBP1 | 46,842,830 | 46,843,730 | 901 | 46,842,824 | 46,843,744 | 304 | Larger |
| 16 | ENSG00000261630 | 53,981,513 | 53,981,649 | 137 | 53,981,501 | 53,981,750 | 137 | Equal |
| 16 | DNAAF1 | 84,170,054 | 84,170,241 | 188 | 84,170,026 | 84,170,319 | 78 | Larger |
| 16 | DNAAF1 | 84,170,054 | 84,170,241 | 188 | 84,170,026 | 84,170,344 | 155 | Larger |
| 16 | ENSG00000260617 | 88,741,734 | 88,741,912 | 179 | 88,741,657 | 88,741,961 | 30 | Larger |
| 16 | SPG7 | 89,540,978 | 89,541,152 | 175 | 89,540,914 | 89,541,308 | 49 | Larger |
| 16 | SPG7 | 89,541,028 | 89,541,152 | 125 | 89,540,914 | 89,541,308 | 49 | Larger |
| 16 | SPG7 | 89,541,028 | 89,541,201 | 174 | 89,540,914 | 89,541,308 | 49 | Larger |
| 16 | ENSG00000260316 | 902,579 | 903,228 | 650 | 902,571 | 905,416 | 46 | Larger |
| 16 | ENSG00000260316 | 904,039 | 904,918 | 880 | 902,571 | 905,416 | 46 | Larger |
| 17 | UBB | 16,382,131 | 16,382,358 | 228 | 16,381,908 | 16,382,590 | 227 | Larger |
| 17 | ENSG00000265401 | 16,382,511 | 16,382,567 | 57 | 16,381,908 | 16,382,590 | 227 | Smaller |
| 17 | LINC02002 | 22,241,163 | 22,241,265 | 103 | 22,240,019 | 22,262,436 | 170 | Smaller |
| 17 | LINC02002 | 22,241,506 | 22,244,558 | 3,053 | 22,240,019 | 22,262,436 | 170 | Larger |
| 17 | LINC02002 | 22,241,509 | 22,244,558 | 3,050 | 22,240,019 | 22,262,436 | 170 | Larger |
| 17 | LINC02002 | 22,244,734 | 22,245,248 | 515 | 22,240,019 | 22,262,436 | 170 | Larger |
| 17 | LINC02002 | 22,245,400 | 22,245,825 | 426 | 22,240,019 | 22,262,436 | 170 | Larger |
| 17 | LINC02002 | 22,245,400 | 22,254,345 | 8,946 | 22,240,019 | 22,262,436 | 170 | Larger |
| 17 | LINC02002 | 22,245,931 | 22,254,345 | 8,415 | 22,240,019 | 22,262,436 | 170 | Larger |
| 17 | ENSG00000285822 | 27,169,373 | 27,169,714 | 342 | 27,167,425 | 27,171,390 | 169 | Larger |
| 17 | ENSG00000285822 | 27,169,373 | 27,169,714 | 342 | 27,169,061 | 27,169,722 | 342 | Equal |
| 17 | RSKR | 28,604,051 | 28,604,275 | 225 | 28,604,043 | 28,604,396 | 45 | Larger |
| 17 | LRRC37A11P | 39,029,056 | 39,029,449 | 394 | 39,028,976 | 39,029,867 | 372 | Larger |
| 17 | ENSG00000263164 | 5,240,587 | 5,240,959 | 373 | 5,240,003 | 5,241,339 | 41 | Larger |
| 17 | ENSG00000263164 | 5,241,041 | 5,241,205 | 165 | 5,240,003 | 5,241,339 | 41 | Larger |
| 17 | ENSG00000262905 | 552,917 | 553,176 | 260 | 552,865 | 553,383 | 65 | Larger |
| 17 | MTMR4 | 58,510,632 | 58,510,734 | 103 | 58,510,620 | 58,510,825 | 103 | Equal |
| 17 | POLR2A | 7,513,773 | 7,513,774 | 2 | 7,513,720 | 7,513,776 | 21 | Smaller |
| 17 | SENP3 | 7,571,871 | 7,571,908 | 38 | 7,571,838 | 7,571,919 | 2 | Larger |
| 17 | ENSG00000267543 | 76,550,234 | 76,550,539 | 306 | 76,549,960 | 76,550,575 | 306 | Equal |
| 17 | MYH10 | 8,481,078 | 8,481,146 | 69 | 8,481,044 | 8,481,181 | 69 | Equal |
| 17 | EIF4A3 | 80,147,023 | 80,147,060 | 38 | 80,146,993 | 80,147,139 | 20 | Larger |
| 17 | ENSG00000262979 | 80,315,724 | 80,316,053 | 330 | 80,313,411 | 80,316,058 | 9 | Larger |
| 17 | ENSG00000266654 | 82,160,339 | 82,160,390 | 52 | 82,160,328 | 82,160,642 | 51 | Larger |
| 17 | CCDC57 | 82,196,799 | 82,196,891 | 93 | 82,196,728 | 82,197,326 | 32 | Larger |
| 17 | CCDC57 | 82,196,799 | 82,196,891 | 93 | 82,196,736 | 82,197,273 | 60 | Larger |
| 17 | CCDC57 | 82,196,799 | 82,196,891 | 93 | 82,196,796 | 82,197,294 | 145 | Smaller |
| 18 | ENSG00000267356 | 14,104,866 | 14,105,033 | 168 | 14,104,830 | 14,105,744 | 84 | Larger |
| 18 | NPM1P2 | 26,205,155 | 26,205,243 | 89 | 26,205,138 | 26,205,266 | 44 | Larger |
| 18 | NPM1P2 | 26,205,437 | 26,205,516 | 80 | 26,205,435 | 26,205,563 | 6 | Larger |
| 18 | NPM1P2 | 26,205,437 | 26,205,516 | 80 | 26,205,435 | 26,205,569 | 30 | Larger |
| 18 | LINC01925 | 514,756 | 515,094 | 339 | 514,517 | 515,287 | 31 | Larger |
| 18 | TXNL1 | 56,600,366 | 56,600,637 | 272 | 56,600,267 | 56,600,743 | 68 | Larger |
| 18 | TXNL1 | 56,600,434 | 56,600,501 | 68 | 56,600,267 | 56,600,743 | 68 | Equal |
| 18 | ENSG00000267675 | 58,416,922 | 58,417,139 | 218 | 58,416,786 | 58,417,228 | 219 | Smaller |
| 18 | TXNDC2 | 9,887,009 | 9,887,412 | 404 | 9,886,747 | 9,887,782 | 45 | Larger |
| 19 | ENSG00000267373 | 16,103,974 | 16,104,100 | 127 | 16,103,969 | 16,104,260 | 126 | Larger |
| 19 | ZNF101 | 19,680,555 | 19,680,855 | 301 | 19,680,345 | 19,680,896 | 304 | Smaller |
| 19 | ZNF93 | 19,934,025 | 19,934,102 | 78 | 19,933,483 | 19,934,844 | 83 | Smaller |
| 19 | LINC01002 | 198,596 | 200,390 | 1,795 | 198,332 | 200,432 | 97 | Larger |
| 19 | LINC01002 | 198,596 | 200,390 | 1,795 | 198,332 | 200,434 | 49 | Larger |
| 19 | LINC01002 | 199,612 | 200,390 | 779 | 198,332 | 200,432 | 97 | Larger |
| 19 | LINC01002 | 199,612 | 200,390 | 779 | 198,332 | 200,434 | 49 | Larger |
| 19 | LINC01002 | 200,050 | 200,390 | 341 | 198,332 | 200,432 | 97 | Larger |
| 19 | LINC01002 | 200,050 | 200,390 | 341 | 198,332 | 200,434 | 49 | Larger |
| 19 | ZNF430 | 21,057,905 | 21,058,070 | 166 | 21,057,009 | 21,058,161 | 83 | Larger |
| 19 | LINC00664 | 21,491,103 | 21,491,213 | 111 | 21,491,099 | 21,491,254 | 71 | Larger |
| 19 | LINC00662 | 27,681,356 | 27,684,505 | 3,150 | 27,680,728 | 27,689,494 | 171 | Larger |
| 19 | LINC00662 | 27,683,584 | 27,684,505 | 922 | 27,680,728 | 27,689,494 | 171 | Larger |
| 19 | LINC00662 | 27,684,608 | 27,684,694 | 87 | 27,680,728 | 27,689,494 | 171 | Smaller |
| 19 | LINC00662 | 27,684,608 | 27,685,056 | 449 | 27,680,728 | 27,689,494 | 171 | Larger |
| 19 | LINC00662 | 27,684,608 | 27,686,871 | 2,264 | 27,680,728 | 27,689,494 | 171 | Larger |
| 19 | LINC00662 | 27,732,448 | 27,732,795 | 348 | 27,732,306 | 27,733,128 | 343 | Larger |
| 19 | LINC00662 | 27,732,448 | 27,732,795 | 348 | 27,732,391 | 27,733,140 | 172 | Larger |
| 19 | TSHZ3 | 31,242,663 | 31,242,782 | 120 | 31,242,464 | 31,242,888 | 60 | Larger |
| 19 | U2AF1L4 | 35,743,206 | 35,743,295 | 90 | 35,743,102 | 35,743,708 | 318 | Smaller |
| 19 | U2AF1L4 | 35,743,209 | 35,743,295 | 87 | 35,743,102 | 35,743,708 | 318 | Smaller |
| 19 | ZNF571 | 37,563,782 | 37,563,823 | 42 | 37,563,752 | 37,563,859 | 34 | Larger |
| 19 | FBXO17 | 38,945,371 | 38,945,751 | 381 | 38,945,370 | 38,945,858 | 64 | Larger |
| 19 | KLK4 | 50,906,553 | 50,906,761 | 209 | 50,906,475 | 50,906,862 | 38 | Larger |
| 19 | ZNF649 | 51,891,399 | 51,891,482 | 84 | 51,891,297 | 51,891,533 | 84 | Equal |
| 19 | ZNF649 | 51,891,399 | 51,891,482 | 84 | 51,891,354 | 51,891,589 | 84 | Equal |
| 19 | ZNF841 | 52,065,566 | 52,066,489 | 924 | 52,065,402 | 52,066,876 | 83 | Larger |
| 19 | ENSG00000269349 | 52,511,994 | 52,512,174 | 181 | 52,511,231 | 52,512,280 | 167 | Larger |
| 19 | NLRP13 | 55,924,659 | 55,924,966 | 308 | 55,924,423 | 55,925,135 | 378 | Smaller |
| 19 | ZNF135 | 58,067,568 | 58,068,407 | 840 | 58,067,342 | 58,068,454 | 84 | Larger |
| 19 | ZNF584-DT | 58,404,826 | 58,405,006 | 181 | 58,404,770 | 58,405,433 | 127 | Larger |
| 19 | ZNF584-DT | 58,404,826 | 58,405,006 | 181 | 58,404,825 | 58,405,269 | 176 | Larger |
| 19 | ENSG00000269371 | 7,520,055 | 7,520,306 | 252 | 7,519,999 | 7,520,341 | 84 | Larger |
| 19 | ENSG00000269371 | 7,520,055 | 7,520,306 | 252 | 7,520,044 | 7,520,400 | 83 | Larger |
| 19 | ENSG00000268120 | 7,870,963 | 7,871,031 | 69 | 7,870,881 | 7,871,133 | 69 | Equal |
| 19 | ENSG00000268120 | 7,870,963 | 7,871,031 | 69 | 7,870,899 | 7,871,088 | 36 | Larger |
| 19 | ENSG00000268120 | 7,870,963 | 7,871,031 | 69 | 7,870,899 | 7,871,173 | 69 | Equal |
| 19 | ENSG00000268120 | 7,870,963 | 7,871,031 | 69 | 7,870,900 | 7,871,119 | 69 | Equal |
| 19 | ENSG00000268120 | 7,870,963 | 7,871,031 | 69 | 7,870,901 | 7,871,199 | 138 | Smaller |
| 19 | ENSG00000268120 | 7,870,963 | 7,871,031 | 69 | 7,870,940 | 7,871,047 | 33 | Larger |
| 19 | PRAM1 | 8,499,475 | 8,499,546 | 72 | 8,499,418 | 8,499,593 | 72 | Equal |
| 19 | PRAM1 | 8,499,475 | 8,499,546 | 72 | 8,499,452 | 8,499,601 | 36 | Larger |
| 19 | PRAM1 | 8,499,475 | 8,499,546 | 72 | 8,499,452 | 8,499,648 | 108 | Smaller |
| 19 | PRAM1 | 8,499,475 | 8,499,546 | 72 | 8,499,454 | 8,499,675 | 108 | Smaller |
| 19 | PRAM1 | 8,499,475 | 8,499,546 | 72 | 8,499,475 | 8,499,643 | 72 | Equal |
| 19 | MYO1F | 8,529,998 | 8,530,074 | 77 | 8,529,803 | 8,530,148 | 77 | Equal |
| 20 | ENSG00000232900 | 10,908,251 | 10,908,359 | 109 | 10,908,213 | 10,908,488 | 151 | Smaller |
| 21 | KRTAP10-12 | 44,697,553 | 44,697,603 | 51 | 44,697,451 | 44,697,719 | 141 | Smaller |
| 21 | ENSG00000280604 | 45,918,648 | 45,918,840 | 193 | 45,918,285 | 45,918,948 | 48 | Larger |
| 21 | PCNT | 46,334,536 | 46,334,610 | 75 | 46,334,453 | 46,334,765 | 39 | Larger |
| 22 | C22orf42 | 32,150,480 | 32,150,991 | 512 | 32,150,480 | 32,152,852 | 1,074 | Smaller |
| 22 | C22orf42 | 32,151,020 | 32,151,486 | 467 | 32,150,480 | 32,152,852 | 1,074 | Smaller |
| 22 | C22orf42 | 32,151,552 | 32,152,066 | 515 | 32,150,480 | 32,152,852 | 1,074 | Smaller |
| 22 | C22orf42 | 32,152,095 | 32,152,561 | 467 | 32,150,480 | 32,152,852 | 1,074 | Smaller |
| 22 | FAM118A | 45,328,064 | 45,328,391 | 328 | 45,328,000 | 45,328,411 | 82 | Larger |
| 22 | MIR3667HG | 49,624,254 | 49,624,446 | 193 | 49,624,047 | 49,624,466 | 38 | Larger |
| X | ENSG00000236491 | 135,194,392 | 135,194,616 | 225 | 135,194,371 | 135,194,621 | 15 | Larger |
| X | SAGE4P | 135,606,917 | 135,607,223 | 307 | 135,606,803 | 135,611,273 | 386 | Smaller |
| X | SAGE4P | 135,607,359 | 135,607,608 | 250 | 135,606,803 | 135,611,273 | 386 | Smaller |
| X | SAGE4P | 135,607,752 | 135,607,998 | 247 | 135,606,803 | 135,611,273 | 386 | Smaller |
| X | SAGE4P | 135,607,752 | 135,607,998 | 247 | 135,607,430 | 135,611,273 | 1,536 | Smaller |
| X | SAGE4P | 135,608,134 | 135,608,381 | 248 | 135,606,803 | 135,611,273 | 386 | Smaller |
| X | SAGE4P | 135,608,134 | 135,608,381 | 248 | 135,607,430 | 135,611,273 | 1,536 | Smaller |
| X | SAGE4P | 135,608,523 | 135,608,768 | 246 | 135,606,803 | 135,611,273 | 386 | Smaller |
| X | SAGE4P | 135,608,523 | 135,608,768 | 246 | 135,607,430 | 135,611,273 | 1,536 | Smaller |
| X | SAGE4P | 135,608,910 | 135,609,157 | 248 | 135,606,803 | 135,611,273 | 386 | Smaller |
| X | SAGE4P | 135,608,910 | 135,609,157 | 248 | 135,607,430 | 135,611,273 | 1,536 | Smaller |
| X | SAGE4P | 135,609,299 | 135,609,545 | 247 | 135,606,803 | 135,611,273 | 386 | Smaller |
| X | SAGE4P | 135,609,299 | 135,609,545 | 247 | 135,607,430 | 135,611,273 | 1,536 | Smaller |
| X | SAGE4P | 135,609,686 | 135,609,930 | 245 | 135,606,803 | 135,611,273 | 386 | Smaller |
| X | SAGE4P | 135,609,686 | 135,609,930 | 245 | 135,607,430 | 135,611,273 | 1,536 | Smaller |
| X | SAGE4P | 135,610,072 | 135,610,317 | 246 | 135,606,803 | 135,611,273 | 386 | Smaller |
| X | SAGE4P | 135,610,072 | 135,610,317 | 246 | 135,607,430 | 135,611,273 | 1,536 | Smaller |
| X | SAGE4P | 135,610,459 | 135,610,707 | 249 | 135,606,803 | 135,611,273 | 386 | Smaller |
| X | SAGE4P | 135,610,459 | 135,610,707 | 249 | 135,607,430 | 135,611,273 | 1,536 | Smaller |
| X | SAGE4P | 135,610,849 | 135,611,093 | 245 | 135,606,803 | 135,611,273 | 386 | Smaller |
| X | SAGE4P | 135,610,849 | 135,611,093 | 245 | 135,607,430 | 135,611,273 | 1,536 | Smaller |
| X | SAGE4P | 135,611,935 | 135,612,179 | 245 | 135,611,669 | 135,614,096 | 378 | Smaller |
| X | SAGE4P | 135,612,321 | 135,612,566 | 246 | 135,611,669 | 135,614,096 | 378 | Smaller |
| X | SAGE4P | 135,612,706 | 135,612,958 | 253 | 135,611,669 | 135,614,096 | 378 | Smaller |
| X | SAGE4P | 135,613,100 | 135,613,351 | 252 | 135,611,669 | 135,614,096 | 378 | Smaller |
| X | SAGE4P | 135,613,493 | 135,613,737 | 245 | 135,611,669 | 135,614,096 | 378 | Smaller |
| X | SAGE3P | 135,638,935 | 135,639,177 | 243 | 135,638,802 | 135,642,377 | 384 | Smaller |
| X | SAGE3P | 135,639,319 | 135,639,557 | 239 | 135,638,802 | 135,642,377 | 384 | Smaller |
| X | SAGE3P | 135,639,699 | 135,640,329 | 631 | 135,638,802 | 135,642,377 | 384 | Larger |
| X | SAGE3P | 135,640,468 | 135,640,717 | 250 | 135,638,802 | 135,642,377 | 384 | Smaller |
| X | SAGE3P | 135,640,859 | 135,641,104 | 246 | 135,638,802 | 135,642,377 | 384 | Smaller |
| X | SAGE3P | 135,641,245 | 135,641,489 | 245 | 135,638,802 | 135,642,377 | 384 | Smaller |
| X | SAGE3P | 135,641,631 | 135,641,877 | 247 | 135,638,802 | 135,642,377 | 384 | Smaller |
| X | SAGE3P | 135,642,019 | 135,642,266 | 248 | 135,638,802 | 135,642,377 | 384 | Smaller |
| X | SAGE2P | 135,674,898 | 135,675,142 | 245 | 135,674,854 | 135,678,801 | 388 | Smaller |
| X | SAGE2P | 135,675,277 | 135,675,519 | 243 | 135,674,854 | 135,678,801 | 388 | Smaller |
| X | SAGE2P | 135,675,663 | 135,675,905 | 243 | 135,674,854 | 135,678,801 | 388 | Smaller |
| X | SAGE2P | 135,676,047 | 135,676,294 | 248 | 135,674,854 | 135,678,801 | 388 | Smaller |
| X | SAGE2P | 135,676,436 | 135,676,683 | 248 | 135,674,854 | 135,678,801 | 388 | Smaller |
| X | SAGE2P | 135,676,825 | 135,677,077 | 253 | 135,674,854 | 135,678,801 | 388 | Smaller |
| X | SAGE2P | 135,677,219 | 135,677,467 | 249 | 135,674,854 | 135,678,801 | 388 | Smaller |
| X | SAGE2P | 135,677,609 | 135,677,854 | 246 | 135,674,854 | 135,678,801 | 388 | Smaller |
| X | SAGE2P | 135,677,996 | 135,678,237 | 242 | 135,674,854 | 135,678,801 | 388 | Smaller |
| X | SAGE2P | 135,678,379 | 135,678,685 | 307 | 135,674,854 | 135,678,801 | 388 | Smaller |
| X | SAGE1 | 135,904,570 | 135,905,251 | 682 | 135,904,277 | 135,906,207 | 770 | Smaller |
| X | SAGE1 | 135,905,393 | 135,906,023 | 631 | 135,904,277 | 135,906,207 | 770 | Smaller |
| X | SAGE1 | 135,906,165 | 135,906,410 | 246 | 135,905,847 | 135,906,607 | 386 | Smaller |
| X | SAGE1 | 135,907,067 | 135,907,312 | 246 | 135,906,647 | 135,911,585 | 388 | Smaller |
| X | SAGE1 | 135,907,454 | 135,907,700 | 247 | 135,906,647 | 135,911,585 | 388 | Smaller |
| X | SAGE1 | 135,907,454 | 135,907,700 | 247 | 135,907,163 | 135,911,026 | 1,155 | Smaller |
| X | SAGE1 | 135,907,842 | 135,908,088 | 247 | 135,906,647 | 135,911,585 | 388 | Smaller |
| X | SAGE1 | 135,907,842 | 135,908,088 | 247 | 135,907,163 | 135,911,026 | 1,155 | Smaller |
| X | SAGE1 | 135,908,230 | 135,908,476 | 247 | 135,906,647 | 135,911,585 | 388 | Smaller |
| X | SAGE1 | 135,908,230 | 135,908,476 | 247 | 135,907,163 | 135,911,026 | 1,155 | Smaller |
| X | SAGE1 | 135,908,618 | 135,908,863 | 246 | 135,906,647 | 135,911,585 | 388 | Smaller |
| X | SAGE1 | 135,908,618 | 135,908,863 | 246 | 135,907,163 | 135,911,026 | 1,155 | Smaller |
| X | SAGE1 | 135,909,005 | 135,909,638 | 634 | 135,906,647 | 135,911,585 | 388 | Larger |
| X | SAGE1 | 135,909,005 | 135,909,638 | 634 | 135,907,163 | 135,911,026 | 1,155 | Smaller |
| X | SAGE1 | 135,909,780 | 135,910,029 | 250 | 135,906,647 | 135,911,585 | 388 | Smaller |
| X | SAGE1 | 135,909,780 | 135,910,029 | 250 | 135,907,163 | 135,911,026 | 1,155 | Smaller |
| X | SAGE1 | 135,910,171 | 135,910,414 | 244 | 135,906,647 | 135,911,585 | 388 | Smaller |
| X | SAGE1 | 135,910,171 | 135,910,414 | 244 | 135,907,163 | 135,911,026 | 1,155 | Smaller |
| X | SAGE1 | 135,910,556 | 135,911,191 | 636 | 135,906,647 | 135,911,585 | 388 | Larger |
| X | SAGE1 | 135,911,333 | 135,911,578 | 246 | 135,906,647 | 135,911,585 | 388 | Smaller |
| X | FMR1-IT1 | 147,947,180 | 147,947,479 | 300 | 147,947,130 | 147,947,685 | 299 | Larger |
| X | AFF2-IT1 | 148,547,167 | 148,547,243 | 77 | 148,547,099 | 148,547,337 | 77 | Equal |
| X | LINC00685 | 321,249 | 321,682 | 434 | 321,093 | 322,609 | 141 | Larger |
| X | TPMTP3 | 51,979,676 | 51,979,730 | 55 | 51,979,674 | 51,979,757 | 23 | Larger |
| X | VCX3A | 6,533,889 | 6,533,948 | 60 | 6,533,759 | 6,533,996 | 30 | Larger |
| X | VCX | 7,843,753 | 7,843,812 | 60 | 7,843,705 | 7,844,002 | 30 | Larger |
| X | VCX | 7,843,873 | 7,843,992 | 120 | 7,843,705 | 7,844,002 | 30 | Larger |
| X | VCX | 7,843,903 | 7,843,962 | 60 | 7,843,705 | 7,844,002 | 30 | Larger |
| Y | RBMY1HP | 21,498,954 | 21,499,394 | 441 | 21,498,453 | 21,500,727 | 547 | Smaller |
| Y | RBMY1HP | 21,499,506 | 21,499,939 | 434 | 21,498,453 | 21,500,727 | 547 | Smaller |
| Y | RBMY1HP | 21,500,051 | 21,500,483 | 433 | 21,498,453 | 21,500,727 | 547 | Smaller |
| Y | RBMY1B | 21,522,494 | 21,522,934 | 441 | 21,521,993 | 21,524,267 | 547 | Smaller |
| Y | RBMY1B | 21,523,046 | 21,523,479 | 434 | 21,521,993 | 21,524,267 | 547 | Smaller |
| Y | RBMY1A1 | 21,546,035 | 21,546,475 | 441 | 21,545,534 | 21,547,808 | 547 | Smaller |
| Y | RBMY1A1 | 21,546,587 | 21,547,020 | 434 | 21,545,534 | 21,547,808 | 547 | Smaller |
| Y | RBMY1D | 21,882,383 | 21,882,816 | 434 | 21,881,574 | 21,883,869 | 547 | Smaller |
| Y | RBMY1D | 21,882,928 | 21,883,368 | 441 | 21,881,574 | 21,883,869 | 547 | Smaller |
| Y | RBMY1E | 21,905,924 | 21,906,357 | 434 | 21,905,115 | 21,907,410 | 547 | Smaller |
| Y | RBMY1E | 21,906,469 | 21,906,909 | 441 | 21,905,115 | 21,907,410 | 547 | Smaller |
| Y | RBMY1F | 22,170,850 | 22,171,283 | 434 | 22,170,046 | 22,172,335 | 548 | Smaller |
| Y | RBMY1F | 22,171,395 | 22,171,834 | 440 | 22,170,046 | 22,172,335 | 548 | Smaller |
| Y | RBMY1J | 22,414,589 | 22,415,028 | 440 | 22,414,088 | 22,416,348 | 547 | Smaller |
| Y | RBMY1J | 22,415,140 | 22,415,573 | 434 | 22,414,088 | 22,416,348 | 547 | Smaller |
| Y | LINC00685 | 321,249 | 321,682 | 434 | 321,093 | 322,609 | 141 | Larger |
| Y | RBMY1A3P | 9,312,736 | 9,313,172 | 437 | 9,312,379 | 9,314,185 | 540 | Smaller |
| Y | RBMY1A3P | 9,313,283 | 9,313,709 | 427 | 9,312,379 | 9,314,185 | 540 | Smaller |
| Y | TSPY6P | 9,507,815 | 9,507,832 | 18 | 9,507,807 | 9,507,851 | 9 | Larger |
| Y | TSPY6P | 9,507,815 | 9,507,832 | 18 | 9,507,807 | 9,507,857 | 18 | Equal |
