# Supplementary material for: The unusual gene architecture of polyubiquitin is created by dual-specific splice sites
Source: Genome Biol. 2024 Jan 24;25:33. doi: 10.1186/s13059-023-03157-8 (PMC10809524; doi:10.1186/s13059-023-03157-8)
Supplement: Supplementary file 2 — Additional file 2: Review history. [file 13059_2023_3157_MOESM2_ESM.docx]

**1st round**

**Reviewer 1**

This manuscript by Duan et al explores the utilization of Dual Specific Splice Sites (DSSs) across the human genome and considers their roles in gene evolution. Understanding perceived accuracy of pre-mRNA splicing and the faithful recognition of exon-intron boundaries has a long standing challenge in gene expression. This problem is exacerbated in metazoan genomes where splice site sequences are degenerate and cryptic splice sites are abundant. In this study, Duan and co-workers search more than 20,000 datasets in the SRA for splice junction reads. Surprisingly, they discover a diverse collection of junction reads dwarfing the annotated junctions found in human gene models. This observation suggests the intriguing hypothesis that many cryptic sites are used, but not represented in the mature transcripts. To test this hypothesis, the authors develop a massively parallel reporter assay in which every 150 bp segment at 20 base pair increments from three genes are tested for their ability to function as 5' or 3' splice sites. This scheme revealed an enrichment in sequences with both 5' and 3'ss activity, many of which where also present in the SRA, supporting their authenticity. Although rare, potential DSS sequences were discovered in the MPRA. Likewise, although DSS were detectable in the SRA at a rate of 3.5% only 0.7% of annotated splice sites had the potential to function as DSSs, thus cryptic sites are more likely to function as DSSs than canonical splice sites. The authors speculate on the function and regulation of splicing at DSS and how these cryptic sites may influence gene evolution. Overall this is an interesting analysis, but is light on mechanistic insights about how DSS regulate gene expression.

The strengths of the manuscript include the significant question being addressed by the authors. Specifically, how is splice site selection so accurate? The answer seems to be that infact, many cryptic sites are indeed used but ultimately do no impact the mature transcript. An additional strength is the combination of the MPRA with a deep dive into publically available datasets in the SRA.

Weakness of the manuscript include clarity of the writing. For example, it is still unclear to me how DSS differ from recursive splice sites. Are the DSS reads identified in the SRA intermediates in the recursive splicing process? Is there any bias towards long introns in the cryptic sites and the DSS identified in this analysis.

The authors show that in general, the DSS 5' and 3'ss sequences are strong, but in specific examples the 3'ss is weak, suggesting some kind of regulatory mechanism to promote utilization of the weak 3'ss. Could this be tested in a reporter assay? The authors also should include citations to papers demonstrating that downstream 5'ss enhance upstream 3'ss. Additionally, the plot shown if Figure 2A is virtually identical to Figure 2 of Zhang et al PNAS 2007. Although this paper is cited, it is probably good to point in the discussion that the authors findings are similar to those reported previously.

The authors should also attempt to clarify the premise and interpretation of the UBC results. Looking at the UBC locus in the genome browser it is not apparent that is widespread usage of DSS. What is the major isoform detected in Figure 4? From the annotations in Figure 4 it is not clear how these isoforms arise from DSS usage - as the 5'ss of exon 2 seems to be paired with the 3'ss of exon 8 or 9.

**Reviewer 2**

In this manuscript by Duan et al., the authors use both multiple reporter assays and datamining of existing RNA-seq data sets to reveal a preponderance of a single sequence element which functions as two different types of splice sites (abbreviated DSS). In other words, a DSS (basically AGGU in the primary transcript) can be either a 5' or 3' ss, speaking to the potential ambiguity or dual purpose role of this sequence. One important finding is that ~50% of the cryptic sites from the multiple reporter assay were also present in the publicly accessible sequenced read archive (SRA), indicating that the spliceosome is much more active than suggested only by the annotated mRNA sequence. This analysis further revealed an interesting feature of the UBC gene. The authors observe variation in splicing within tandem repeats of the coding regions resulting in either retained or spliced 'introns' which code for polypeptides. This feature - introns which reside precisely within the tandem repeat register of a protein - appears in other genes as well, suggesting a mechanism where splicing might control the multimerization of repeated monomers in such proteins. Overall, I found the paper to be well written and persuasive. It's also timely, given several recent reports of extensive recursive splicing and/or variation in branchpoint selection. I have a few comments:

1. Details of the sequencing analysis are a bit light (quality control, thresholds, etc.). They cite ref 23, but it is unclear if they are using a methodology in that paper or just acknowledging a similar analysis. Also, one thing I was wondering: have the authors ever looked at nascent sequencing?
2. The MPRA seems a little underdeveloped, leading me to think that perhaps it is not very quantitative (which is fine), but the authors might consider several comments about analyzing this data in greater depth since it is a clever assay.
- I was left wanting a plot of the measured enrichment values for each gene. Can the authors provide insight into the difference between the splice sites detected in both MPRA and SRA and those detected in only MPRA? For example, do the splice sites present in both datasets have higher enrichment values?
- Furthermore, I was left wanting a plot of measured enrichment values for individual splice sites. In some ways, it would be satisfying to see that enrichment depends on the splice site sequence and not its position in the reporter. Since each splice site is tested multiple times in different positions in the reporter, the authors should be able to determine how position in reporter impacts measured enrichment.
- How is enrichment of each site related to the MaxEnt scores in figure 2? Naively, I would expect a strong correlation between these two metrics.
4. Additionally the figure itself has some smaller issues that need to be fixed:
- Figure 1B should show the location of splice site identified on all three genes. It would not take very much more space.
- In figure 1D, not surprisingly, the authors find that -AG dominates at 3' splice site. However, it's interesting that there are many examples where splicing occurs at non-AG 3' SSs. The authors should address why this could be.
- In figure 1E, the gel needs to be labeled better. For example, it's not clear what each lane represents. Also, the sentence, "we performed RT-PCR on the BRCA2 gene and were able to detect low levels of transcript isoforms that used the unannotated splice sites identified by our MPRA" needs clarification. I observe a few faint bands at higher mw but I have no idea whether this is indeed inclusion of a cryptic exon or PCR side products. The authors need to make a more convincing case on this point.
5. Is it accurate to say the cryptic 3' motif (Fig. 1D) shows less preference at the -3 position (i.e. 'C' in 'CAG).
6. Figure 2A: The authors claim that DSSs (AGGT used as 5'ss and 3'ss) have high MaxEnt scores for 5'ss and 3'ss usage. However, it seems to the reviewer that there are large proportions of cryptic 5'ss and cryptic 3'ss groups that also have high MaxEnt scores for both 5'ss and 3'ss (scores higher than gray dashed lines). Since the n is small for DSSs (n=24) found in their MPRA, can the authors include statistical significance between the three groups to support their claim? Is the same trend also observed for their SRA junction read analysis?
7. Figure 2B: The authors argue that DSSs are underrepresented in annotated splice sites, yet their definition of DSS seems to be inconsistent in the two datasets, MPRA and SRA. For MPRA, only AGGT-containing cryptic splice sites used as 5'ss or 3'ss are considered as DSSs. However, for SRA, all sites containing 5'ss and 3'ss junctions identified with a read number threshold are defined as DSSs. The fair comparison would be to consider AGGT-containing annotated splice sites. The authors found that 14% of cryptic 3' splice sites have AGGT (441,107 / 3,156,210), which may be different for annotated 3' splice sites. It would be helpful if the authors can clarify this. Also, can the authors also present the frequency of DSSs in annotated 5'ss and all 5'ss in Figure 2B?
8. Figure 5 shows that many introns are embedded in tandem repeats with the intron size being larger, equal to, or shorter than the repeat size. In the text, the authors state: "If an intron's size is a multiple of a repeat's size, it splices via a DSS because different occurrences of the same sequence function as a 5'ss and 3'ss (see Fig. 5b and 5c)." However, the figure does not show any evidence that these sites are AGGT-containing DSS or that they are splice sites. To support their statement, the authors should at least include information (either in the figure or supplemental table) showing the location of AGGT and whether they were identified as a DSS in their analysis (similar to Figure 3).
9. Finally, they argue that DSS in tandem repeat genes plays a role in evolution of repeat domains. The motivation for this is simply that these regions are not highly conserved. This argument is a bit weak. Could the authors provide additional evidence to strengthen their hypothesis?

**Authors’ response**

Reviewer 1

This manuscript by Duan et al explores the utilization of Dual Specific Splice Sites (DSSs) across the human genome and considers their roles in gene evolution. Understanding perceived accuracy of pre-mRNA splicing and the faithful recognition of exon-intron boundaries has a long standing challenge in gene expression. This problem is exacerbated in metazoan genomes where splice site sequences are degenerate and cryptic splice sites are abundant. In this study, Duan and co-workers search more than 20,000 datasets in the SRA for splice junction reads. Surprisingly, they discover a diverse collection of junction reads dwarfing the annotated junctions found in human gene models. This observation suggests the intriguing hypothesis that many cryptic sites are used, but not represented in the mature transcripts. To test this hypothesis, the authors develop a massively parallel reporter assay in which every 150 bp segment at 20 base pair increments from three genes are tested for their ability to function as 5' or 3' splice sites. This scheme revealed an enrichment in sequences with both 5' and 3'ss activity, many of which where also present in the SRA, supporting their authenticity. Although rare, potential DSS sequences were discovered in the MPRA. Likewise, although DSS were detectable in the SRA at a rate of 3.5% only 0.7% of annotated splice sites had the potential to function as DSSs, thus cryptic sites are more likely to function as DSSs than canonical splice sites. The authors speculate on the function and regulation of splicing at DSS and how these cryptic sites may influence gene evolution. Overall this is an interesting analysis, but is light on mechanistic insights about how DSS regulate gene expression. The strengths of the manuscript include the significant question being addressed by the authors. Specifically, how is splice site selection so accurate? The answer seems to be that in fact, many cryptic sites are indeed used but ultimately do no impact the mature transcript. An additional strength is the combination of the MPRA with a deep dive into publicly available datasets in the SRA.

Weakness of the manuscript include clarity of the writing. For example, it is still unclear to me how DSS differ from recursive splice sites.

Thanks - we apologize for our difficulty describing the differences between recursive sites (RSSs) and dual-specific splice sites (DSSs). We have tried to improve clarity by making an additional figure in the supplement that explicitly compares the two classes of site and adding text to introduction.

The basic idea in boolean terms would be DSSs functioning as a 5'ss OR a 3'ss and the recursive functioning as a 3'ss AND THEN a 5'ss or in reverse order (i.e. 5'ss AND THEN a 3'ss).

Part of the problem is these labels are not mutually exclusive - a site could be a DSS and a recursive site. Both elements contain the core ‘AG’ of the 3'ss immediately upstream of the core ‘GT’ of the 5'ss. While a DSS is a sequence that can function as a 5’ss or a 3'ss, this is not necessarily true for a RSS. The sequence of a recursive site changes between the first and second splice so it is possible for a recursive splice located in an intron to only function as a 3'ss, initially, but then gain 5'ss function after the introduction of new upstream context.

We modified the text and included it in supplementary Figure 1 to provide a comparison between DSSs and RSSs.

Are the DSS reads identified in the SRA intermediates in the recursive splicing process?

No, the DSS reads cannot technically be intermediates of recursion because the DSS has reads that could not arise from recursive splicing. The SRA infers splicing events from a gapped alignment of exon/exon junctions to the genome. A DSS is defined by two distinct exon/exon junctions. However in recursive splicing, the first splice transfers the exonic context of the original substrate to the intermediate so the second splicing event creates an exon/exon junction that is indistinguishable from a process that skipped over the recursive site. The second splicing event also destroys the junction created by the first splicing event so it should be found at lower levels than a DSS. Having said that, I would not be surprised if many DSSs could also be RSSs in some situations. In some ways, this is an issue of language. We felt it was important to use the terms from the original Zhang paper but, for us, the larger biological question was “Can a DSS go on to complete another splicing event?” There is a clear underrepresentation of DSSs in annotated sites - we reasoned that the few DSSs found in annotated sites could be prone to exon skipping as this would be the consequence of a second splicing event. However we didn’t find an enrichment of skipping nor lariats that would prove this mechanism. So if a DSS can induce further splicing events, this additional activity is clearly selected against in DSSs that encode annotated splice sites.

Is there any bias towards long introns in the cryptic sites and the DSS identified in this analysis?

No. We divided the annotated introns into four bins for MPRA and ten bins for SRA based on their sizes. Within each bin, we calculated the frequency of cryptic splice sites and DSSs, which was determined by dividing the total count of cryptic splice sites or DSSs by the length of the intron within which these cryptic splice sites or DSSs are located. Our results suggest that there is no evident bias towards longer introns.

The authors show that in general, the DSS 5' and 3'ss sequences are strong, but in specific examples the 3'ss is weak, suggesting some kind of regulatory mechanism to promote utilization of the weak 3'ss. Could this be tested in a reporter assay?

We tried testing multiple candidate weak 3’ss in a reporter assay but did not get strong enough basal splicing from the constructs to test this experimentally. We then tried to address your concern by comparing Maxent scores for DSSs, distinguishing between 5' splice sites and 3' splice sites. This analysis has been added to an updated Figure 2. For both the MPRA (Fig. 2a) and SRA (Fig. 2b) DSSs, we find that they have weaker splice sites relative to AGGT that are only used as either 3’ss or 5’ss. This weakness can be observed qualitatively in the sequences from the SRA sites as DSSs have GC rich sequences with more poorly-defined polypyrimidine tracts and 5’ss motifs (Fig 2c). This result may suggest that DSSs have a lower splicing activity than single splice sites. However, even when controlling for SRA junction read counts as a proxy for usage, we find that at similar levels of splicing activity DSSs are weaker than single splice sites (Additional file 1: Fig. S2). Thus, DSSs are able to support splicing with weaker motifs. We point out this ability of a second element to rescue non consensus splice sites is computational definition of an enhancer (i.e. RESCUE approach) and further supports a synergy between 5’ss and 3’ss recognition.

The authors also should include citations to papers demonstrating that downstream 5'ss enhance upstream 3'ss.

This is a good point and ties the finding to the exon definition data of the ‘90s. Zhang et al originally described DSS as zero length exons but did not report the enhancement properties. We modified the text in the discussion to place it in this context.

Additionally, the plot shown if Figure 2A is virtually identical to Figure 2 of Zhang et al PNAS 2007. Although this paper is cited, it is probably good to point in the discussion that the authors findings are similar to those reported previously.

This is a good suggestion. As you say, we cited Zhang et al and we also included their experiment from figure 2 in our original discussion, but we modified the text to ensure their contribution was appropriately recognized. The difference between the original and our experiment is we used examples of DSSs outside of the annotation whereas Zhang only uses annotated splice sites. In our work we find that DSS activity in AG/GT sequences in annotated 3'ss is about 4.7-fold underrepresented, implying selection against dual use in annotated sites. We find a synergy between 5'ss and 3'ss at AG/GT sequences whereas Zhang proposed a competition in the annotated sites. We suggest the difference between our results may reflect the purifying selection on Zhang’s annotated sites.

The authors should also attempt to clarify the premise and interpretation of the UBC results. Looking at the UBC locus in the genome browser it is not apparent that is widespread usage of DSS. What is the major isoform detected in Figure 4? From the annotations in Figure 4 it is not clear how these isoforms arise from DSS usage - as the 5'ss of exon 2 seems to be paired with the 3'ss of exon 8 or 9.

The major isoform detected in figure 4 is the transcript that contains all 9 ubiquitin units.

The yellow boxes in Figure 4 represent the ubiquitin units rather than exons. In actuality, ubiquitin units 1, 2, 8, and 9 span across two exons. The DSS of ubiquitin unit 2 (acting as a 5'ss) pairs with the DSS of ubiquitin unit 7 (acting as a 3'ss) which results in the upper isoform shown in Figure 4. Similarly, the DSS of ubiquitin unit 2 pairs with the DSS of ubiquitin unit 8, which leads to the formation of the lower isoform depicted in Figure 4. We made modifications to Figure 4 by incorporating the exon isoforms that encompassed the ubiquitin units. Additionally, we have included the sequences of these two isoforms in the supplementary file.

Reviewer 2

In this manuscript by Duan et al., the authors use both multiple reporter assays and datamining of existing RNA-seq data sets to reveal a preponderance of a single sequence element which functions as two different types of splice sites (abbreviated DSS). In other words, a DSS (basically AGGU in the primary transcript) can be either a 5' or 3' ss, speaking to the potential ambiguity or dual purpose role of this sequence. One important finding is that ~50% of the cryptic sites from the multiple reporter assay were also present in the publicly accessible sequenced read archive (SRA), indicating that the spliceosome is much more active than suggested only by the annotated mRNA sequence. This analysis further revealed an interesting feature of the UBC gene. The authors observe variation in splicing within tandem repeats of the coding regions resulting in either retained or spliced 'introns' which code for polypeptides. This feature - introns which reside precisely within the tandem repeat register of a protein - appears in other genes as well, suggesting a mechanism where splicing might control the multimerization of repeated monomers in such proteins. Overall, I found the paper to be well written and persuasive. It's also timely, given several recent reports of extensive recursive splicing and/or variation in branchpoint selection. I have a few comments:

1. Details of the sequencing analysis are a bit light (quality control, thresholds, etc.). They cite ref 23, but it is unclear if they are using a methodology in that paper or just acknowledging a similar analysis.

Thank you for this comment. We are using the methodology and dataset (i.e. Intropolis) created by ref 23. We have added the following text to Methods to describe how the intropolis data is used.

“We extracted the 4-bp sequences for the 5' splice sites (2bp upstream of the 5'ss and the 5'ss itself) and 3’ splice sites (the 3'ss itself and 2bp downstream of the 3'ss) listed in intropolis. Our focus was solely on dual-specific splice sites (DSSs) with an AGGT motif. A site that includes the AGGT motif and demonstrates the capability to function as both a 5'ss and a 3'ss, each with a minimum read count of 10, is identified as a DSS.”

Also, one thing I was wondering: have the authors ever looked at nascent sequencing?

No, we didn’t look at nascent sequencing. But this is a good suggestion. Certain alternative transcripts serve as intermediates and do not persist in the final RNA product.

2. The MPRA seems a little underdeveloped, leading me to think that perhaps it is not very quantitative (which is fine), but the authors might consider several comments about analyzing this data in greater depth since it is a clever assay.

- I was left wanting a plot of the measured enrichment values for each gene. Can the authors provide insight into the difference between the splice sites detected in both MPRA and SRA and those detected in only MPRA? For example, do the splice sites present in both datasets have higher enrichment values?

Thanks for your insightful suggestion. We performed the comparison you suggested which revealed splice sites detected in both our MPRA and the SRA exhibit notably higher enrichment scores as you predicted. We added the outcome of your suggestion to Figure 1.

- Furthermore, I was left wanting a plot of measured enrichment values for individual splice sites. In some ways, it would be satisfying to see that enrichment depends on the splice site sequence and not its position in the reporter. Since each splice site is tested multiple times in different positions in the reporter, the authors should be able to determine how position in reporter impacts measured enrichment.

Our approach was to average usage over the 6 or 7 registries. The possibility of using the different registers to map positive and negative elements across the tiling array was something we want to do in the future, but it seemed beyond the scope of this paper. However, we have included this information in the supplementary data in a genome browser track so others can use it as a resource.

- How is enrichment of each site related to the MaxEnt scores in figure 2? Naively, I would expect a strong correlation between these two metrics.

I think this is a reasonable expectation but we observed only a moderate correlation between MaxEnt scores and enrichment scores. We have seen a similarly modest relationship in other studies. The lack of a stronger correlation probably comes down to the role of surrounding context (outside the window of PWM) in splice site recognition and maybe the inability of the 3'ss PWM to handle large gaps.

4. Additionally the figure itself has some smaller issues that need to be fixed:

- Figure 1B should show the location of splice site identified on all three genes. It would not take very much more space.

We added two other genes into Figure 1B.

- In figure 1D, not surprisingly, the authors find that -AG dominates at 3' splice site. However, it's interesting that there are many examples where splicing occurs at non- AG 3' SSs. The authors should address why this could be.

The utilization of a few non-AG 3' splice sites could stem from the presence of specific auxiliary sequences or regulatory elements in close proximity. These elements could interact with the spliceosome machinery, facilitating effective splicing at these unconventional splice sites.

An alternate possibility is non-canonical sites could be minor spliceosome introns. From a sequence standpoint, this hypothesis would predict AC 3’ss. However, the data indicates non canonical sites are more likely to be GG. It has been observed that U12 spliceosome can process introns with a mixture of U2 and U12 cis-elements so it is possible that there is more flexibility in U12 sites than is appreciated. To address this possibility, we searched events from the SRA for U12 introns. We found AT/AC introns to be more prevalent in the SRA detected introns than in the annotated splice sites. Prior work from the Sharp lab (Burge et al 1998) suggested evolutionary pressure was transitioning U12 introns to U2 introns at the annotated sites. It is possible that U12 splicing is occurring at a higher level in rare SRA sites. In our opinion, this was interesting but fairly speculative and somewhat off topic. Until a more in-depth analysis is needed to prove these differences and a study of U12 splicing is a topic outside the scope of the paper.

A larger issue, in my opinion, is non-canonical sites are an indicator of high error rates in the SRA. In the initial submission we reported overlap in MPRA and SRA detected DSSs corroborating about 40% of the sites, so we wanted to further address this by looking at orthogonal measures of function. Here we analyzed the lariats recovered from lariat-seq (a method to map lariat-derived cDNAs) looking at the subset of DSS that had 5’ss which could splice to annotated 3’ss. We report lariat read data for splicing events to these DSSs that corroborates the exon/exon junctions from the SRA (Additional file 1: Table S1). We also modified the discussion section to include the lariat data in the supplement. Our inclination was to not include the U12 data because it would distract from the main focus of the paper.

- In figure 1E, the gel needs to be labeled better. For example, it's not clear what each lane represents. Also, the sentence, "we performed RT-PCR on the BRCA2 gene and were able to detect low levels of transcript isoforms that used the unannotated splice sites identified by our MPRA" needs clarification. I observe a few faint bands at higher mw but I have no idea whether this is indeed inclusion of a cryptic exon or PCR side products. The authors need to make a more convincing case on this point.

The products were confirmed by Sanger sequencing. We have modified the text and replotted Figure 1E to make it clearer. Each lane represents one of three pseudo- exons created by cryptic sites identified in our MPRA. The faint bands at higher molecular weight indeed represented the inclusion of a cryptic exon, confirmed by Sanger sequencing.

5. Is it accurate to say the cryptic 3' motif (Fig. 1D) shows less preference at the -3 position (i.e. 'C' in 'CAG).

Yes, it is correct. We agree that the cryptic 3'ss shows less preference at the -3 position. We have added your comment to the text.

6. Figure 2A: The authors claim that DSSs (AGGT used as 5'ss and 3'ss) have high MaxEnt scores for 5'ss and 3'ss usage. However, it seems to the reviewer that there are large proportions of cryptic 5'ss and cryptic 3'ss groups that also have high MaxEnt scores for both 5'ss and 3'ss (scores higher than gray dashed lines). Since the n is small for DSSs (n=24) found in their MPRA, can the authors include statistical significance between the three groups to support their claim? Is the same trend also observed for their SRA junction read analysis?

When comparing the MaxEnt scores for 5'ss, we observed that the MaxEnt score for 5'ss within DSSs is notably lower than that of AGGT which only function as 5’ss. This same pattern can also be seen when examining the MaxEnt scores for 3'ss with DSSs having weaker splice sites than AGGT which only function as 3’ss. This analysis has been added to an updated Figure 2. We performed the same analysis on DSSs from the SRA data and found that they also have weaker splice sites relative to AGGT that are only used as either 3’ss or 5’ss (Fig. 2b). This weakness can be observed qualitatively in the sequences from the SRA sites as DSSs have GC rich sequences with more poorly-defined polypyrimidine tracts and 5’ss motifs (Fig 2c). This result may suggest that DSSs would have a lower splicing activity than single splice sites. However, even when controlling for SRA junction read counts as a proxy for usage, we find that at similar levels of splicing activity DSSs are weaker than single splice sites (Additional file 1: Fig. S2). Thus, DSSs are able to support splicing with weaker substrate sequences, indicating a synergy between 5’ss and 3’ss recognition.

7. Figure 2B: The authors argue that DSSs are underrepresented in annotated splice sites, yet their definition of DSS seems to be inconsistent in the two datasets, MPRA and SRA. For MPRA, only AGGT-containing cryptic splice sites used as 5'ss or 3'ss are considered as DSSs. However, for SRA, all sites containing 5'ss and 3'ss junctions identified with a read number threshold are defined as DSSs. The fair comparison would be to consider AGGT-containing annotated splice sites. The authors found that 14% of cryptic 3' splice sites have AGGT (441,107 / 3,156,210), which may be different for annotated 3' splice sites. It would be helpful if the authors can clarify this.

I think we may have failed to make clear that we used the same definition to identify DSSs in both our MPRA analysis and SRA analysis. We have added the following sentence to the Methods to clarify this:

“We extracted the 4-bp sequences for the 5'ss (2bp upstream of 5'ss and the 5'ss itself) and 3'ss (the 3'ss itself and 2bp downstream of 3'ss) listed in the Sequence Read Archive. Our focus was solely on AGGT splicing events. A site that includes the AGGT motif and demonstrates the capability to function as both a 5'ss and a 3'ss, each with a minimum read count of 10, is identified as a DSS.”

Also, can the authors also present the frequency of DSSs in annotated 5'ss and all 5'ss in Figure 2B?

We have included in the updated Figure 2 the frequencies of DSSs within the sets of annotated 5'ss and all 5'ss from the SRA.

8. Figure 5 shows that many introns are embedded in tandem repeats with the intron size being larger, equal to, or shorter than the repeat size. In the text, the authors state:

"If an intron's size is a multiple of a repeat's size, it splices via a DSS because different occurrences of the same sequence function as a 5'ss and 3'ss (see Fig. 5b and 5c)."

However, the figure does not show any evidence that these sites are AGGT-containing DSS or that they are splice sites. To support their statement, the authors should at least include information (either in the figure or supplemental table) showing the location of AGGT and whether they were identified as a DSS in their analysis (similar to Figure 3).

We have included this information in supplementary figure 7. While we restricted our analysis to DSSs with an AGGT motif in the MPRA and SRA sections of the study for simplicity, we did not apply this restriction in the section addressing introns in tandem repeats. We did so because of our observation that, given an intron within a tandem repeat, if the intron’s length is a multiple of the repeat unit’s length then its 5’ss and 3’ss would be DSSs regardless of whether they used the predominant AGGT motif.

9. Finally, they argue that DSS in tandem repeat genes plays a role in evolution of repeat domains. The motivation for this is simply that these regions are not highly conserved. This argument is a bit weak. Could the authors provide additional evidence to strengthen their hypothesis?

I’m sorry for the misunderstanding. We clearly failed to explain our hypothesis and the evidence that supported it. We have altered Figure 5 and re-written the accompanying text.

Our observation from looking at existing introns in repeat regions is that introns that are a multiple of the repeat unit length are 8.4-fold enriched relative to expectation. For these ‘special cases’ where an intron is a multiple of the repeat unit, by definition uses the same sequence at the 5’ss and 3’ss. In considering this as a potential mechanism for de novo intron creation, we were struck by the following facts:

a) Mutational likelihood

An intron requires a 5’ss and a 3’ss. Cryptic sites are usually activated by single point mutations. A mutation that creates a DSS is one sequence that can provide both splice sites through repeat expansions that is at least 10^3^ times more likely than point mutations.

b) Selection

Expansions (and any other type of mutation) are more likely to be deleterious than beneficial. The enrichment of introns that are a multiple of repeat length means an expansion could be spliced out and therefore not affect the message.

We added this to a model of de novo intro creation in Figure 5c.

**2^nd^ round**

**Reviewer 1**

The author's made all of the recommended suggestions from my initial review. I think the manuscript more clearly articulates the interesting evolutionary aspects of dual specific splice sites in the UBC gene (and beyond).

Nice job on the revision!

**Reviewer 2**

In this revision from Duan et al., the role of dual sites seems much clearer, either through changes in the text or perhaps just the passage of time. I will re-iterate that the MPRA assay is a nice complement to the analysis of SRA, and the UBC case is fascinating. Overall, it is a nice study and likely to be an impactful contribution. One is left with the feeling that there is still a fundamental layer in final splice site selection the field is still missing.
One minor suggestion:
1. A zoomed region of a single intron in Fig. 1 B would be a nice addition.
